# Structural Hierarchy and Maturation of Amyloid Fibrils Revealed by Interface Descriptor Analysis

**DOI:** 10.1021/acs.jcim.6c01376

**Published:** 2026-08-21

**Authors:** Máté Sulyok-Eiler, Veronika Harmat, András Perczel

**Affiliations:** † Medicinal Chemistry Research Group, 280964 HUN-REN Research Centre for Natural Sciences, Magyar Tudósok Körútja 2, H-1117 Budapest, Hungary; ‡ Laboratory of Structural Chemistry and Biology, Institute of Chemistry, ELTE Eötvös Loránd University, Pázmány P. stny. 1/A, H-1117 Budapest, Hungary; § Hevesy György PhD School of Chemistry, Institute of Chemistry, Eötvös Loránd University, Pázmány P. stny. 1/A, H-1117 Budapest, Hungary; ∥ HUN-REN-ELTE Protein Modeling Research Group, Hungarian Research Network, Pázmány P. stny. 1/A, H-1117 Budapest, Hungary

## Abstract

Here, we present a comprehensive, topology-driven analysis of 543 amyloid structures (all cryo-EM-determined parallel amyloid fibril structures available in the PDB and the Amyloid Atlas, covering multiple protein types, notably Tau and α-synuclein) utilizing a novel automated protocol, ACWF. By calculating per-residue interface descriptors—shape complementarity (Sc), buried surface area (Ab), and surface detail index (SDi)—via a sliding window approach, we quantify structural packing and interdigitation across ∼30,000 local interfaces. We introduce a sequence-overlap-modified RMSD (RMSDmod) metric for hierarchical clustering to robustly classify polymorphs and quantify structural diversity. Our results reveal that mature amyloid fibrils contain a mixture of tightly and loosely packed regions, with distinct interaction hot-spots characteristic to polymorph families. Clustering successfully distinguishes disease-specific topologies and tracks maturation pathways, demonstrating that ex vivo fibrils rearrange to more compact structures indicated by more buried side chains (larger Ab) and typically lowered Sc values compared to that seen in the case of in vitro produced fibrils formed over shorter time scales. This work establishes a topology- and interface-based framework that links residue-level interactions to polymorphic fibril evolution, exploring the most compacted as well as accessible and attackable regions.

## Graphical abstract

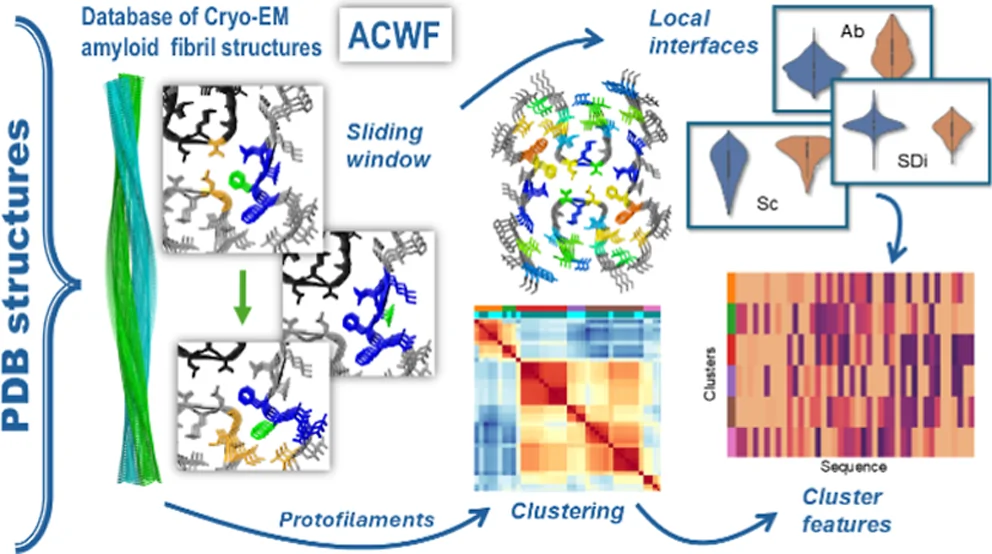

## 1 Introduction

Amyloid fibrils are insoluble misfolded protein aggregates accumulating in organs and tissues.^1^ Pathogenic amyloids are implicated in more than 30 diseases, most of which are age-related and manifest as characteristic organ-specific deposits.^2,3^ While neurodegenerative diseases such as Alzheimer’s disease (AD) and chronic traumatic encephalopathy (CTE) are associated with Tau proteins,^4^ and Parkinson’s disease (PD) with α-synuclein^5^ (α-syn), all share the common feature of the coaccumulation of amyloid-β-peptide (Aβ) deposits. Amyloid formation is not restricted to the nervous system. For example, islet amyloid polypeptide (IAPP) accumulates in the pancreatic islets, while systemic amyloidosis affects multiple organs through the aggregation of proteins such as transthyretin^6^ (TTR) and immunoglobulin light chains^7^ (LC). TTR amyloidosis may present predominantly with cardiomyopathy or peripheral and autonomic neuropathy, whereas LC amyloidosis can involve several organs, including the heart, kidneys, and eyes. Certain amyloids also serve biological functions, such as reversible, vesicular storage of peptide hormones in the form of compact amyloid aggregates.^8,9^

The defining structural hallmark of all amyloid fibrils is the cross-β diffraction pattern,^10^ characteristic of the long-range association of mated β-sheets. Fibril architectures can be resolved by solid-state NMR^11^ or cryo-EM,^12^ with the latter increasingly prevalent due to recent technological advances. As illustrated in Figure 1, protein chains adopt a pseudo-2D-fold, typically sampling extended β-strand segments, with turn and loop conformations in between (Figure 1d). Repeated stacking of this pseudo-2D motif produces β-sheets with H-bonds oriented along the fibril axis. Naturally occurring amyloids so far have proven to be overwhelmingly of parallel topology, alongside a few examples showing an antiparallel β-sheet core.^13,14^ Side-chain interactions between mated β-sheets form steric zippers, stabilizing the fibrillar 3D structure.^12^ Protofilaments consist of these repeating chains, and multiple protofilaments can assemble into a single fibril. Slight rotations of stacked chains produce a gently helical twist, characterized by rise (around 4.8 Å) and twisting angle (typically 1–2° per chain). The crossover distance, representing half the helical pitch where twisting filaments cross over, can be calculated from these two (Figure 1e,f).

**Figure 1:**
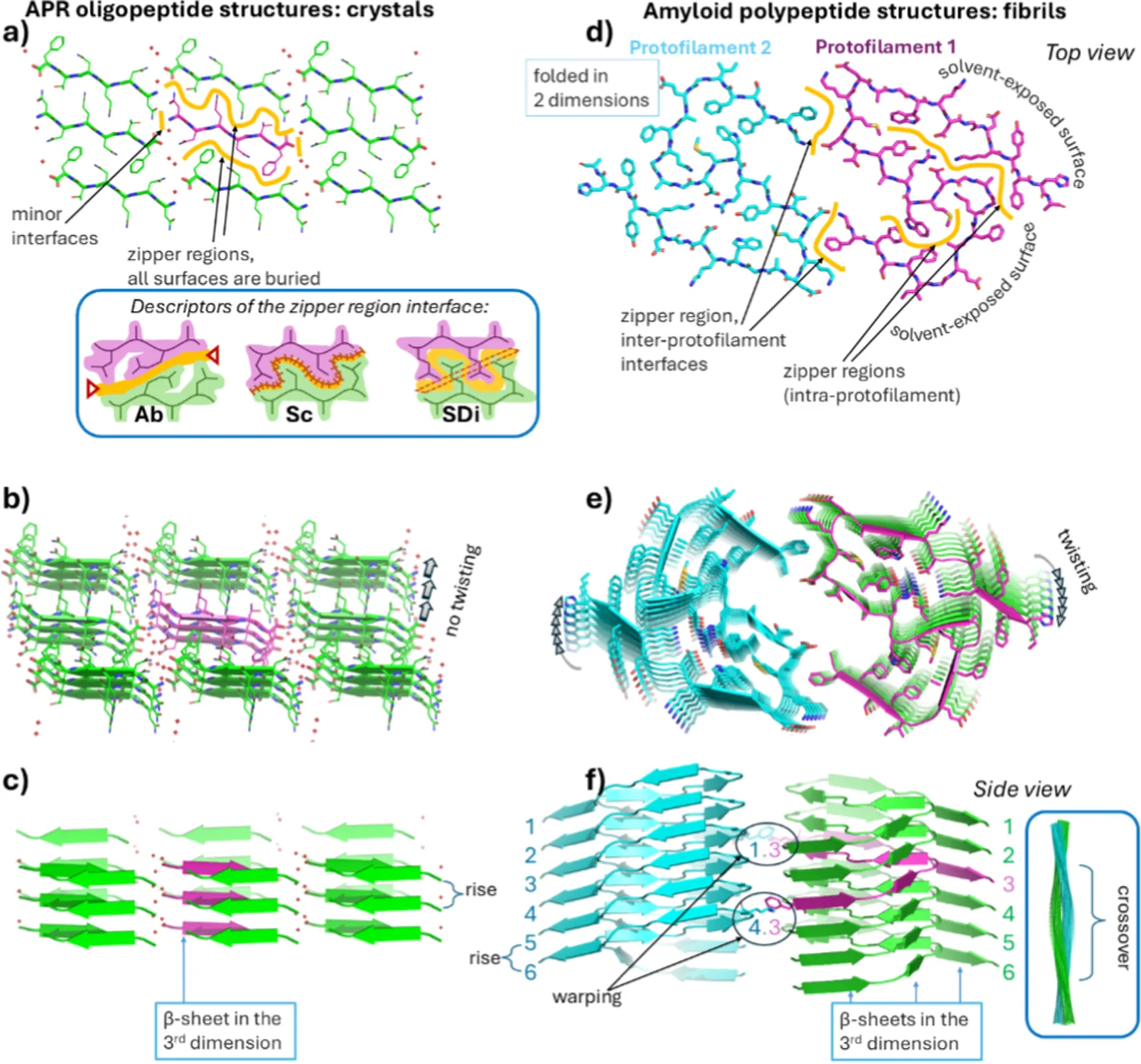
Structural and topological features of APR oligopeptide crystals (a–c) and amyloid polypeptide fibrils determined by cryo-EM (d–f) (represented by 2 examples: PDB 3FVA and 6ZCF, respectively). (a,d) Assembly of 2D packing in APRs and 2D-folds in polypeptide chains occurs through contacting molecular surfaces. Their complementarity and topology are quantified by the 3 interface descriptors: Ab, Sc, and SDi (inset in a,b,e) Structural repetition arises from translational stacking in APR crystals and from helical twisting of polypeptide chains in fibrils. (c,f) Primary organizing force in the third dimension is β-sheet formation. In some fibril structures, the 2D-folded polypeptide chain is significantly bent along the fibril axis, resulting in a warping effect, where different regions of a chain interact with multiple neighboring chains along the axis. The mesoscopically observable crossover distance originates from fibril twisting (inset in f).

A critical unresolved issue in amyloid research concerns the relationship between aggregation-prone regions (APRs) and the global fibril architecture. APRs are short peptide segments capable of self-assembling into amyloid-like fibrils or crystals^15–18^ (Figure 1a). While APRs have been extensively characterized, the formation of full-length fibrils and especially the role of the incorporated non-APR sequences remain poorly understood. (Fibril-like structures of APRs from here on will be referred to as APRs, while the term fibril will be reserved for fibrils of physiologically relevant longer segments.) Fibrillar 2D-folds involve multiple β-sheets connected by β-arcs,^19^ creating complex interfaces that can differ from the simpler, largely planar interfaces observed in APR crystals^20^ (Figure 1b,e). Warping^12^ along the fibril axis allows contacts beyond immediate neighbors (Figure 1f), resulting in topological diversity, which contributes to amyloid polymorphism.

In our recent work,^21^ standardized interface descriptors, including shape complementarity (Sc), buried surface area (Ab), and a novel surface detail index (SDi), were used to analyze the APR crystal structures available in the PDB,^22^ revealing six interface classes among them, with characteristic secondary interaction patterns. Interestingly, in amyloid fibrils, the same APR segments participate in multiple zipper interfaces, forming varied interaction networks that are not yet fully rationalized.^23^

It is curious that in amyloid polypeptide fibrils the same APR region was found to be involved in different zipper regions in various sets of interactions, various other segments also forming interactions. However, the roles of these different segments in forming the 2D-folds is not fully understood. Recent studies aimed to analyze the energetic contributions of different segments to the amyloid fold; the results showed that the stabilization energy is similar in different polymorphs of α-syn and Tau; the same stabilizing segments formed different interaction networks.^23^

Here, we extend our topological and complementarity-based analyses to full-length amyloid fibrils. By identifying and characterizing sterically optimal interfaces—which also proved to be interaction hot-spots—within the 2D-fold, our approach quantifies individual residue contributions and allows comparison of structure-based clusters, reflecting polymorphic variations. We created the ACWF plugin that automates the workflow: it consists of a database of amyloid structures from the PDB, calculates the 3 descriptors, and visualizes their values. This strategy offers a topology-driven perspective on fibril assembly, enabling rational interpretation of the significance of both APR and non-APR segments and the structural impact of point mutations, while providing a framework for understanding maturation and polymorphism in amyloid fibrils.

## 2 Results and Discussion

### 2.1 Workflow for Structural Analysis

Our goal was to develop a tool capable of analyzing the wide range of amyloid fibril structures available in the PDB^22^ and Amyloid Atlas^12^ (Scheme 1). First, relevant (see chapter 2.2) fibril structures were selected from these databases (**1**). Protofilaments were then extended by repeating their chains to generate fibrils of sufficient length for accurate buried interface calculations, avoiding edge effects at protofilament termini (**2**). The fibril axis was defined, and key geometric parameters, including radius and warping angle, were computed (**3**). Local interfaces around each side chain were determined using a sliding window along the 2D-folded polypeptide chain (**4**). Interface descriptors, shape complementarity (Sc), buried surface area (Ab), and surface detail index (SDi) were calculated for each local interface (**5**). Hierarchical clustering based on pairwise sequence overlap-modified RMSD values enabled assessment of structural variability between polymorphs and allowed calculation of cluster-averaged local descriptors (**6–7**). Clustering allows for comparison of interface descriptors across different folds and localization of local cores within the 2D-folds (**8**). Finally, the ACWF plugin provides color schemes to visualize Sc, Ab, and SDi values and facilitates the traversing of the database by selecting protein type, protofilament number, and clustering. New structures can be analyzed by repeating interface calculations **(2–5)**.

**Scheme 1:**
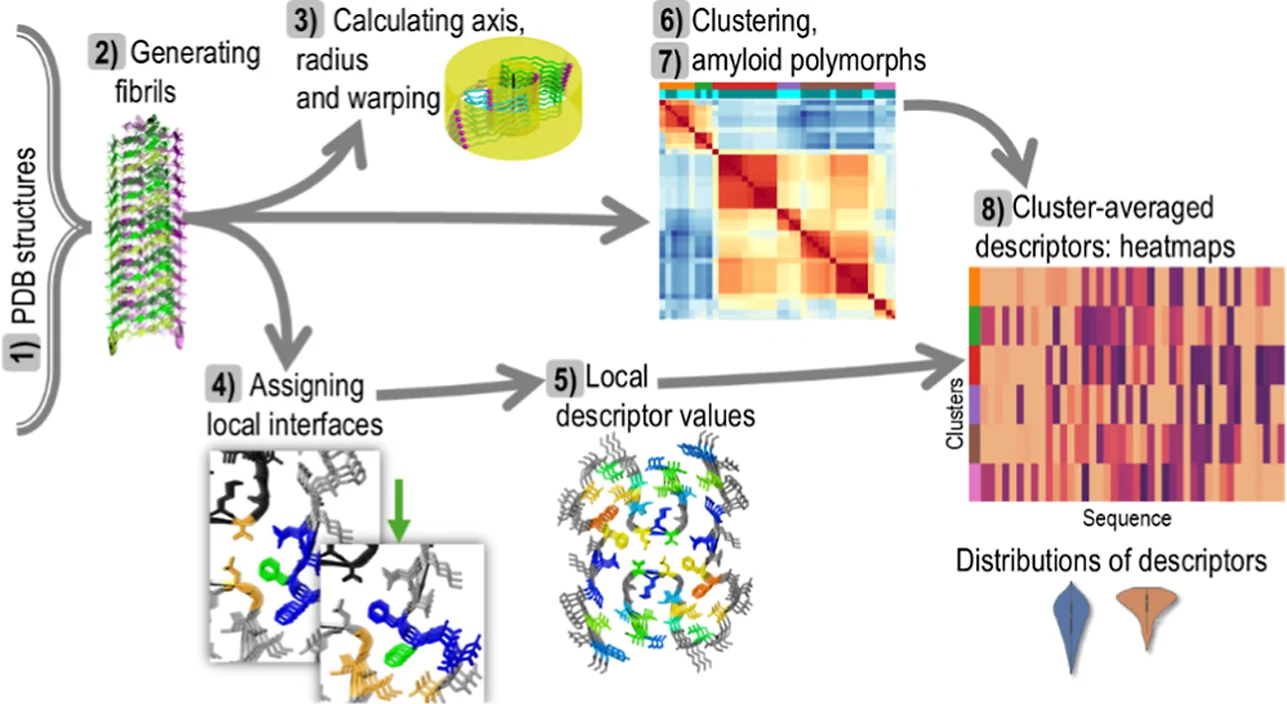
Steps of Structural Analysis of Amyloid Fibril Structures

### 2.2 Data Set for Analysis of Amyloid Fibril Structures

Rapid advances in cryo-EM have enabled high-resolution determination of amyloid fibrils from both in vitro and ex vivo sources. We based our analysis on all available experimental structures from the PDB^22^ and Amyloid Atlas^12^ of parallel topology, excluding only those where (1) protofilaments were poorly defined (no fibril could be built) and (2) molecular surfaces could not be calculated (missing side chains). Although our previous work^21^ highlighted significant differences between parallel and antiparallel amyloid interfaces, the scarcity of antiparallel fibril structures deposited to date currently prevents their comparative analysis. Thus, our final data set comprises 543 parallel fibrils (1064 protofilaments), all determined by cryo-EM (Table S1). The data set is diverse, spanning over 20 proteins, though Tau and α-synuclein dominate with 32% and 26% of structures, respectively (Figure 2a and Table S1).

**Figure 2:**
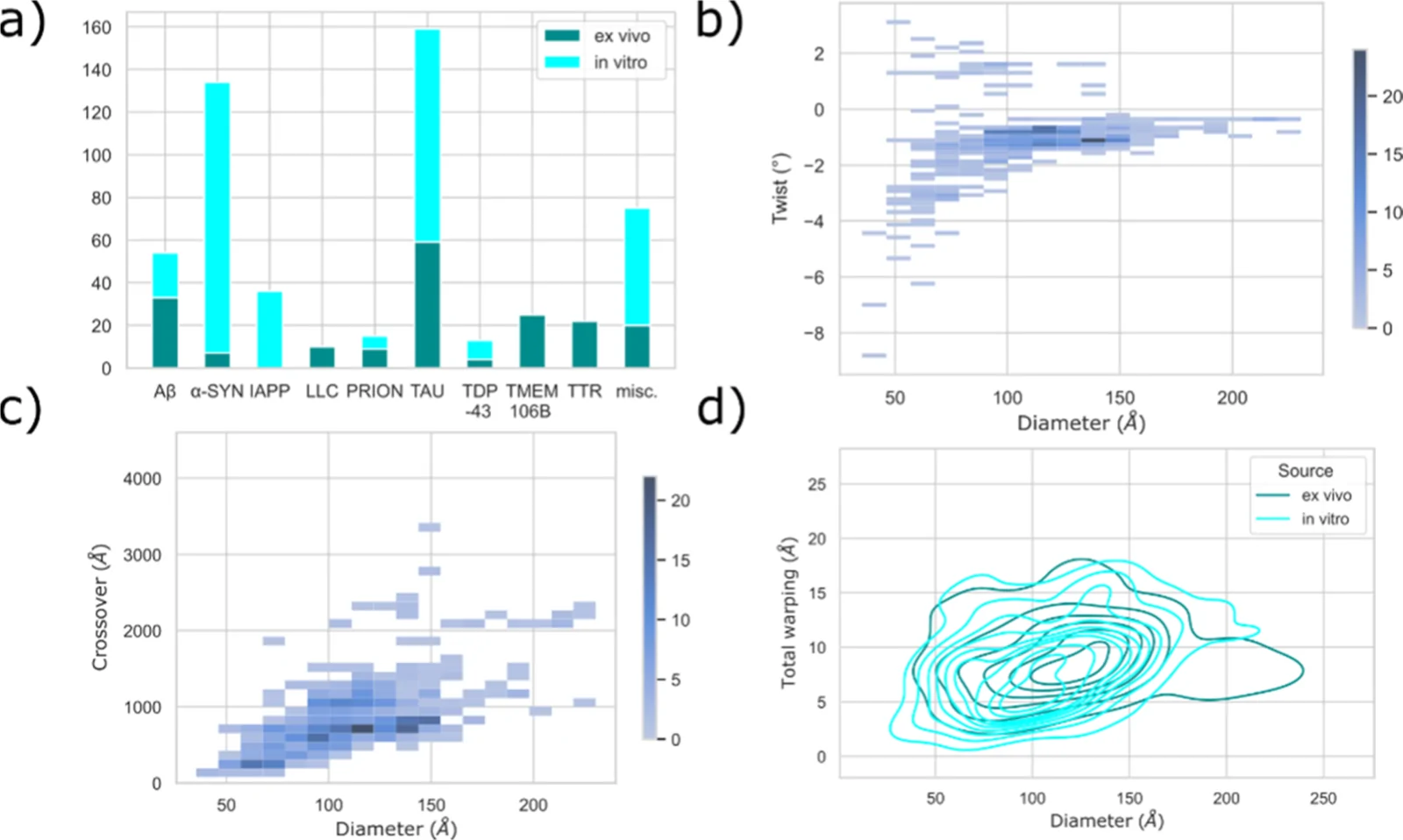
Global characteristics of the amyloid fibril structures (ACWF database). (a) Distribution of fibrils from in vitro and ex vivo sources across different proteins; nine most populated groups are shown separately, while the remaining structures are in the last column. Joint 2D density plots showing the relationship between fibril diameter and twisting angle (b) and diameter and crossover distance (c). (d) Kernel density estimate of crossover distance and total warping for in vitro and ex vivo fibrils.

The majority of the structures are in vitro preparations, while only 37% are from ex vivo sources (Figure 2a). Notably, ex vivo α-syn fibril structures are rare (only 5% of all α-syn structures), while Transmembrane protein 106B (TMEM106B) and TTR structures are all from ex vivo sources. Most fibrils exhibitleft-handed twisting (negative angles), with only 31 right-handed examples (Figure 2b). Wider fibrils tend to show smaller twisting angles and correspondingly larger crossover distances (Figure 2c). The largest absolute twist values occur in shorter β-sheet segments with simple 2D-folds (e.g., PDB 6QJM). Twist and crossover distributions for in vitro and ex vivo fibrils are generally similar, except for extremely long crossover distances, observed only ex vivo (Figure 2d). Total warping was measured as the distance between the lowest and highest Cα atoms along the fibril axis (parallel to axis *z*; Figure 1f). We found that this value varies between 2–20 Å and shows no correlation with diameter or crossover distance, although ex vivo fibrils tend to have a slightly higher total warping value (∼5 Å; Figure 2d).

In summary, simpler folds exhibit greater twisting and smaller crossover distances. Meanwhile, the diameter correlates weakly with crossover values. Ex vivo structures (which can be considered to be more mature fibrils) often have the largest crossover distances. Total warping is independent of the other global parameters.

### 2.3 Structural Descriptors Characterizing the Zipper Region Interfaces Redefined for Per-Residue Environments

Our previous study^21^ analyzing inter-β-sheet interfaces within APR structures using steric fit (Sc), interdigitation (SDi), and buried area (Ab) demonstrated that these complementary descriptors distinguish parallel from antiparallel structures and categorize short interfaces into characteristic groups. Since APRs represent tightly packed amyloid seeds, it is informative to compare their interfaces with those of polypeptide fibrils using the same descriptors (steric fit (Sc), interdigitation via the surface detail index (SDi), and buried area (Ab)) while accounting for fibril-specific structural features. Amyloid fibril 2D-folds are not always flat: warping of the fold can bring residues up to 20 Å apart into contact. To capture all relevant interactions, we applied a structural elongation algorithm that aligns, copies, and shifts H-bonded protofilament chains. (Fibril axis position and direction were not standardized in structural databases and thus were not used.)

Amyloid fibril 2D-fold also features large, complex, and meandering buried surfaces in contrast to the open-ended, straight interfaces in APR crystals. These surfaces often contain bends, turns, junctions, and dead ends. To handle this complexity, we defined local interfaces per residue using a sliding window approach: for each side chain, contacting residues on the opposing surface were identified and assigned to a local interface based on sequence proximity (see Methods). This method samples every side chain contributing to an extended buried surface, yielding 29,361 local interfaces, typically spanning 3–6 residues. To ensure comparability, we also reanalyzed APR crystal structures using this sliding window method. In our previous work,^21^ we treated each APR β-sheet zipper surface as a single interface. Sliding-window segmentation of these surfaces produces consistently smaller Ab values, while the Sc and SDi distributions obtained using the two methods are very similar (orange vs green violin plots in Figure 3a,c,e), validating the per-residue approach for both fibrils and APRs. It should be noted that the over-representation of Tau and α-syn structures in our database possibly biases the overall distributions of descriptors toward the characteristics of these two proteins, so we calculated the distributions for each protein and performed a detailed analysis of per-residue features as discussed in the following sections.

**Figure 3:**
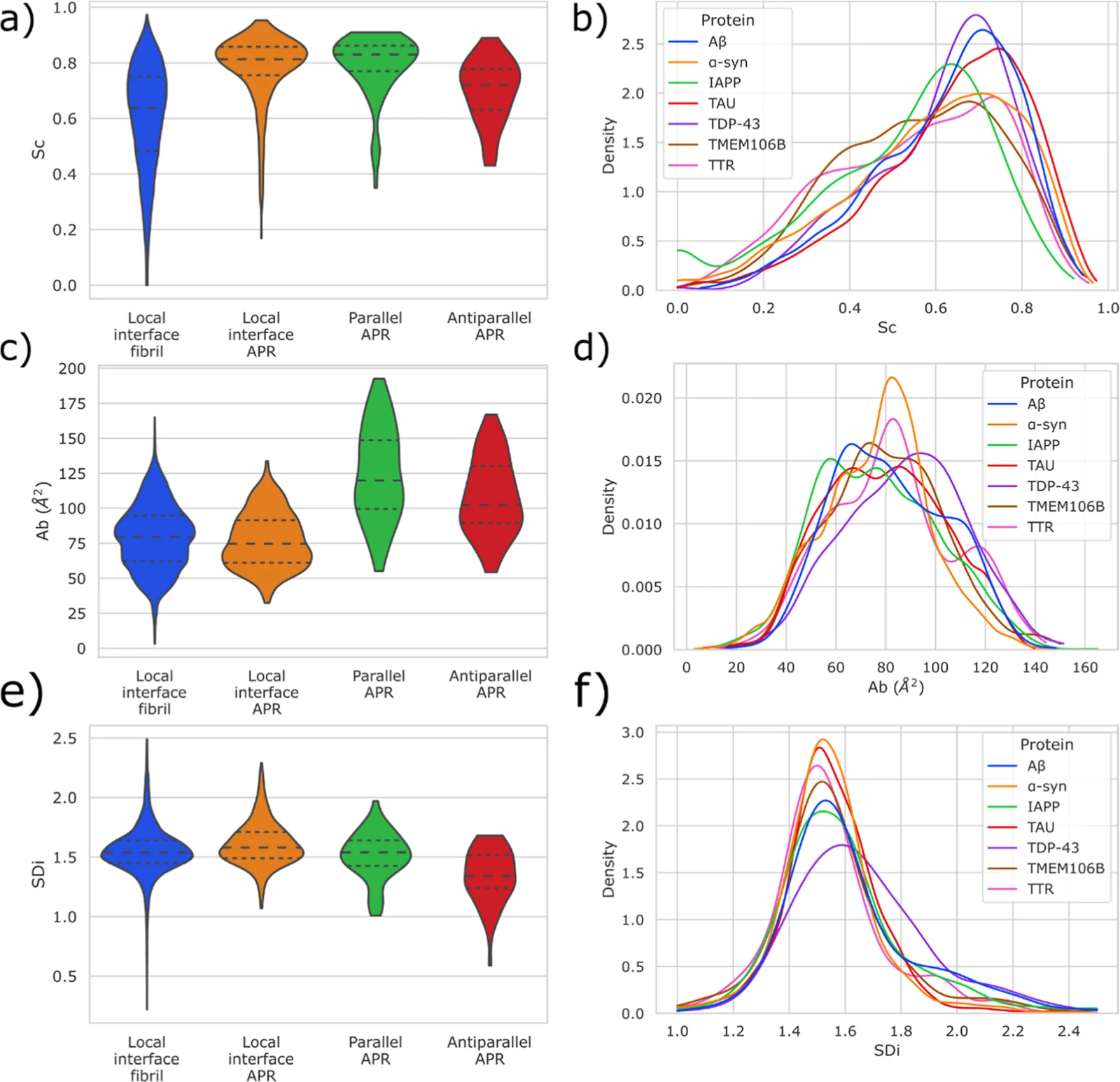
Distributions of descriptor values (Sc, Ab, and SDi) for local interfaces. (a,c,e) Comparison of distributions in fibrils (blue) and APR crystals (orange) for parallel structures, shown as violin plots with median and quartiles (dashed lines). Reference APR oligopeptide distributions from our previous study^21^ are shown in green and red. (b,d,f) Protein-specific distributions of local descriptors for seven proteins with more than 15 protofilament structures in the database (Aβ: blue, α-syn: orange, IAPP: green, Tau: red, TDP-43: purple, TMEM106B: brown, TTR: pink). Kernel density plots highlight sequence-specific packing characteristics.

### 2.4 Comprehensive Analysis of Ab, Sc, and SDi Descriptors for Oligopeptides and Polypeptide Fibrils

Distributions of Ab, Sc, and SDi for local interfaces were calculated to characterize global zipper properties in mature fibrils and amyloid-prone regions (APRs) as protofilament seeds (Figure 3a,c,e). Fibrils display broader distributions for all 3 descriptors compared to APRs, reflecting the presence of both loosely and tightly packed interface regions. In contrast, APRs tend to be tightly packed, with solvent channels mainly at β-sheet edges. To identify sequence-specific characteristics, fibrils from proteins represented by more than 15 protofilament structures in the database were analyzed separately (Figure 3b,d,f).

1) Sc, reflecting the ratio of loosely versus tightly packed regions, shows a right-skewed distribution in polypeptide fibrils (Figure 3a) with an average of 0.5 and a median of 0.7, lower than the APR average of 0.75. High shape complementarity is typical of APRs;^21^ however, in mature fibrils, these high Sc regions are rare. The distribution exhibits mostly medium Sc regions, meaning high Sc values are essential for protofilament seed formation but have a lesser role in mature filaments. Protein-specific Sc distributions (Figure 3b) reveal that Aβ, α-syn, and IAPP fibrils retain higher shape complementarity, resembling APRs in this respect.
2) Ab values are multimodal, reflecting the number and size of residue involvement. The distribution is very similar, proving the validity of the method (Figure 3c), albeit showing rare extreme cases. The difference of the two modes can be explained by the excess number of termini of the peptide chains, as these side chains are expected to have smaller local buried interfaces. Protein-specific Ab comparisons (Figure 3d) indicate that high Ab values are not solely dictated by side-chain abundance. For instance, TDP-43 displays large Ab values, although amino acid side chains of large buried surface areas are more frequent in the case of Tau, TTR, and TMEM106B (Figure S1).
3) SDi distribution for fibrils is narrow, left-skewed, and unimodal, averaging 1.5, similar to APRs (Figure 3e,f), indicating comparable interdigitation levels. Most proteins show very similar distributions (Figure 3f)—A notable exception is TDP-43, which has highly interdigitated (SDi > 1.8) local interfaces, often surface β-sheet pairs with long side chains forming embedded patterns. The most interdigitated fibril interfaces (SDi > 2) occur independently of protein type, associated with side chains buried in 2D-folded elements such as sharp backbone kinks (α-syn, PDB structure 6SST, Val74) or T-junctions (serum amyloid A, PDB structure 6MST, Phe3).

A comparison of intra- and interprotofilament interfaces reveals a strong sequence dependence—appearing markedly different across different proteins. Interprotofilament interactions are frequent and sometimes predominant in Aβ fibrils, whereas intra- and interprotofilament ratios are low in Tau and α-syn fibrils. Two common types of interprotofilament interfaces were identified: double salt bridges involving paired charged residues (e.g., α-syn, PDB id: 7XO1 Lys45···Glu46) and hydrophobic interfaces extending over 3–15 residue segments that form zippers. The latter generally exhibit higher Sc and indicate efficient solvent exclusion (e.g., α-syn, PDB id: 7XJX residues 50–57; and Aβ, PDB id: 8OT1 residues 15–40). Solvent channels may split these segments, as in the 6XYQ structure of α-syn residues 43–50. The local Ab and SDi distributions resemble intraprotofilament interactions, Sc bimodality, as observed for α-syn, highlights the differences between hydrophobic and salt-bridge interprotofilament interfaces (Figure S2).

Overall, the distributions of Sc and Ab indicate that fibrils contain both tightly and loosely packed regions, the most highly packed regions being even more compacted than those of APR oligopeptides; however, these are rare. The ratio is protein-dependent and may serve as an indicator of packing efficiency. The overarching descriptor distributions are inherently skewed by the heavy representation of Tau and α-synuclein structures in current databases. Indeed, the distribution of the descriptors is protein-dependent (Figure 3b,d,f). To mitigate this compositional bias and prevent overgeneralization, we implemented a sequence-overlap-modified RMSD clustering approach to systematically categorize structural folds independent of their raw abundance.

### 2.5 Different Levels of Polymorphism

Amyloid fibrils have a remarkable characteristic, as even a single protein sequence can form multiple β-sheet 2D-folds, sometimes coexisting within the same sample. Our approach allowed us to explore the structural hierarchy of polymorphism across sequence-related fibril structures. Some proteins, like TTR,^24^ form only a single 2D-fold with minor backbone variations focused to a well-defined, short segment (residues 54–70 in the case of TTR). In contrast, other proteins adopt multiple distinct folds, occasionally involving different regions of the polypeptide, with certain folds linked to specific pathologies, such as C-shaped Tau folds in AD and CTE patients.^25^ Fibrils can be (and are) categorized by source, morphologies, or implicated pathology. However, since polymorphism is inherently structural, spanning levels of both the 2D-folds and protofilament organization, more robust classification could be achieved based on pairwise 3D comparisons. Connor et al.^23^ recently applied such an approach to α-syn and selected Tau structures, emphasizing the energetic contributions of highly overlapping sequence segments. While energetics offers important insight into stabilization, its quantitative evaluation for large, heterogeneous fibrillar systems remains model-dependent. Here, we adopt a complementary perspectiveby focusing on the topology of buried interfaces within diverse 2D-folds. Topology provides a more direct and well-defined descriptor of fibril organization, while energetics offers an interpretative layer. Together, the two approaches outline different but mutually informative aspects of amyloid polymorphism. Here, we modified the result of the pairwise fitting to accommodate the sequence overlaps, resulting in RMSD_mod_ (eq 1). Using this allowed us to avoid false positives from short but well-fitting segments and to cluster protofilaments from different parts of the sequence. Proteins with highly diverse folds (α-syn, Tau) form more distant clusters, whereas TMEM106B and TTR show closely related ones. These structure-based clusters encompass previously identified polymorphs and allow exploration of subclasses, quantification of diversity, selection of representative 2D-folds (medoid structures of clusters), and identification of distant polymorph families.

Tau fibrils are the most populated group and show the greatest structural variability, reflected in high RMSD_mod_ variance between protofilament pairs. Major 2D-fold differences often correspond to the presence/absence of sequence repeats. J- and C-shaped Tau folds dominate clusters ***C4*** and ***C5***, respectively, containing R3:R4:Nter segments, which are more closely related to each other than to other folds (Figure 4, dendrogram). Clusters may include structures that were characterized as different polymorphs based on the patients they were extracted from; e.g., cluster ***C5*** contains AD- and CTE-related folds^25^ indicating structural similarity, while ***C4*** contains precursors of the C-shaped fold. Highly divergent folds, such as Pick, in vitro globular glial tauopathy (GGT), and heparin-induced folds,^26–28^ form a loosely related cluster ***C2*** (Figure 4 inset and Table 2), suggesting a common core topology.

**Figure 4:**
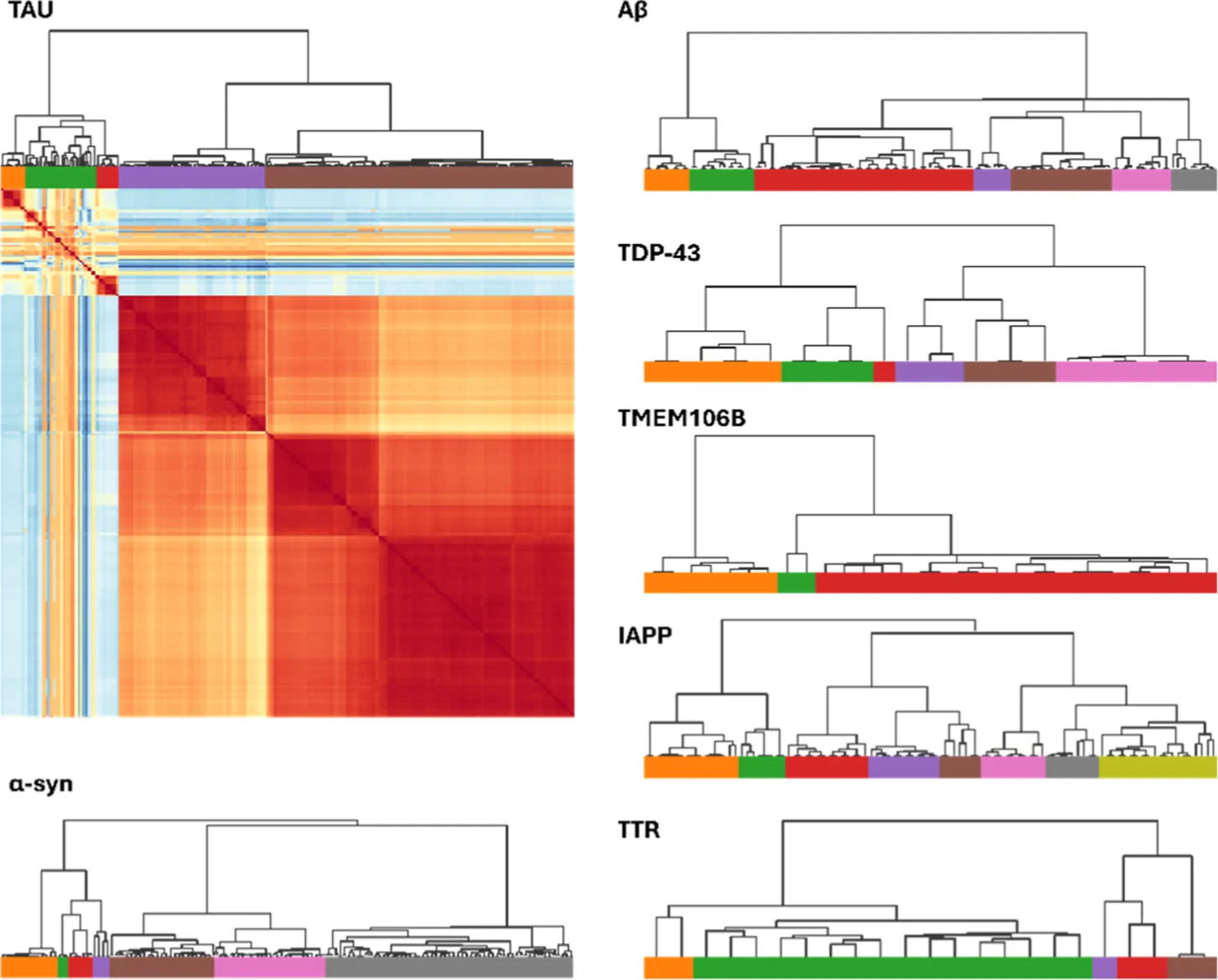
Clustering of amyloid protofilament structures based on pairwise RMSD_mod_ values corrected for sequence overlap. Dendrograms show hierarchical relationships on a relative scale; the number of clustered protofilaments and cluster RMSD_mod_ variance are summarized in Table 1. Clusters were defined using a 30% variance limit for each polypeptide type and color-coded as ***C1***: orange, ***C2***: green, ***C3***: red, ***C4***: purple, ***C5***: brown, ***C6***: pink, ***C7***: gray, and ***C8***: olive. Inset: Tau dendrogram with RMSD_mod_ heatmap (red: small, blue: large) as an example. High-resolution dendrograms with heatmaps and PDB codes are provided in Supporting Information Figures S3–S9.

α-syn structures also exhibit high variability. The most populated cluster, ***C7***, shows low internal variance, indicating similar 2D-fold chains form polymorphs such as rod and twister,^29^ MSA types I and II, and minor-late-PD^30^ (Table 2). Three in vitro α-syn folds dominate: Type II and III (pH 5.8–7.4) in ***C5*** and Type I (pH > 7)^31^ in ***C6***, with additional clusters representing closely related 2D-folds (***C5***: Löwestam Type 1A/2A,^32^ PD Fan 1A/1B;^30^ ***C6***: polymorphs 2a/2b^33^). Other α-syn clusters represent divergent, post-translational modification (PTM) specific or mutation-specific structures.

Aβ structures show moderately large RMSD_mod_ variance (Figure S3). Some folds investigated by solid-state NMR (e.g., U-shaped Aβ40, S-shaped Aβ42)^34^ are not represented in cryo-EM structures; these probably would form separate clusters. Current clustering groups partially overlapping structures, where shared zipper interfaces define 2D-fold cores: hydrophilic cores (cluster ***C1***: Glu11, His13, Lys16, and His6) or hydrophobic cores (cluster ***C5***: Phe19, Phe20, Val24, Asn27, Ile31 plus Met35, Ile32, and Val40). The largest and most diverse cluster, ***C3***, organizes a zipper around Met35 with various lengths of the 2D-folded chain; further Aβ analyses are in Section 2.8.

IAPP shows moderate structural variability with eight clusters (***C1***–***C8***), which can be sorted to four groups of loose similarity (Figures 4 and S6). The structures within the clusters are less diverse: one or two 2D-folds are represented in each of them. Typically, IAPP filaments contain protofilaments of different 2D-folds belonging to different classes (Table 2). TDP-43, TMEM106B, and TTR have fewer PDB structures; thus, several of their clusters contain only one or two protofilaments (Figures S7–S9). TDP-43 clusters remain isolated, whereas TMEM106B clusters ***C2*** (Singlet IIIa/IIIb^35^) and ***C3*** (Singlet I, Doublet) show more similarity. TTR fibrils are structurally least variable (RMSD_mod_ variance 6.56 Å^2^, Table 1), with clusters differing only in backbone conformation of residues 57–70 (open gate, closed gate, and absent/broken gate), including the cardiac wild-type fold^24^ (Table 2).

**Table 1:** Structure-based Clustering of Amyloid Fibril Protofilaments^a^

	structures	protofilaments	clusters	average *RMSD*_*mod*_ for all protofilament pairs [Å]	variance of *RMSD*_*mod*_ for all protofilament-pairs [Å^2^]
Tau	159	301	5	12.28	220.23
α-syn	134	263	7	11.30	142.51
Aβ	54	125	7	9.12	90.18
TDP-43	13	25	6	9.76	50.45
IAPP	36	97	8	8.66	57.85
TMEM 106B	25	30	3	3.86	22.34
TTR	22	23	5	1.96	6.71

a Clustering was performed using Ward’s method^36,37^ on RMSD_mod_ values of pairwise comparisons of protofilament middle chains. Cluster variance was limited to 30% of the total RMSD_mod_ variance within each dataset.

**Table 2:** Signature Residues of Structural Clusters in Seven Polypeptide Protofilament**s** ^a^

polypeptide, cluster id	variance of RMSD_mod_ [Å^2^]	medoid structure of the cluster	no. of residues with >90% of the highest value of		residues with top 5 highest Sc values (bold), and SDi values (underlined)	notes
			Sc	SDi		
**Tau**						
C1	20.93	7P67/1	6	3	**Q307** D314 **C322** **L357** **V363** N368 T373 **E380**	GGT types 1,2,3; GPT types 1a, 1b and 2;^25^ PHF1 phosphomimetic tau^38^
C2	63.56	8PPO/2	16	14	K395 **V398** **L408** **N410** I417 L428 **A437 Q439** L441	diverse group of structures (e.g., heparin-induced;^26^ new folds from in vitro experiments (including in vitro GGT);^28^ Pick fold^27^)
C3	17.04	6VH7/2	3	4	D295 **H299 P301 S305** V306 **Q307 I308** V309 Y310	AGD types 1 and 2;^25^ CBD types I and II^39^
C4	58.10	8Q9C/2	9	3	Y310 **S320** K321 L325 **P332 I354** **L357** **V363** N368	J-shaped folds; Intermediate amyloid structures of AD and CTE types, sharing similar folds^40^
C5	27.66	9CZL/2	12	21	Y310 V318 C322 **L325** I328 **H330 P332 V337** V350 **V363**	C-shaped folds of AD and CTE types^40–42^
α-syn						
C1	8.13	8 × 7B/1	7	9	**V52** T54 E61 **V70** **V74** **A76** **A78** K80 S87	mutant forms, E46K and G51D
C2	1.14	6L1U/2	2	10	**M1** T22 Y39 **S42** E46 **Q62 A69** V74 V82	Y39-phosphorylated^43^
C3	26.69	8ZMY/1	11	5	V15 **A17** T22 Q24 **A27 A29** V52 **Q62 A91**	polymorph 5A;^31^ lipid-complexed polymorphs 1A, 1B and 1C^44^
C4	24.83	9D5C/2	1	3	F4 **M5** **A19** **A53** **V74** **A76** A91	S84-modified^45,46^
C5	21.86	7 V48/1	3	2	L38 **V40** T44 **V49** **V71** **T72** V74 **T75** V77 V82	Löwestam type1A, 2A;^32^ PD Fan type 1A, 1B^30^; most PD-seeded filaments^30^
C6	28.30	8A4L/2	7	6	**A17 A19** T44 **V49** V52 V71 **T72** V74 **V77**	polymorphs 2a and 2b^33^ Löwestam type 1B, 2B
C7	30.74	8Y2Q/1	8	5	**A27 A29** T33 **V49** H50 **A69** I88 **Q109**	polymorph 1a (rod polymorph) and polymorph 1b (twister polymorph);^29^ MSA-Type I and II; PD Fan Type3; minor-late-PD;^30^ C-truncated^47^
**Aβ**						
C1	8.09	8QN6/2	2	3	**E11 Q15 K16** V18 **F20** E22 N27 I32 **M35**	Aβ1–40; morphology I^34^; type 1 filament^48^
C2	11.48	8OT1/1	1	1	**V24** N27 **K28 I31** **L34** **V36**	Aβ1–40 β-strand-pair, unseeded morphology I and II;^49^ lipidic Aβ1–40 polymorphs L2 ^50^
C3	26.06	7Q4M/1	0	1	**Y10** N27 **I31 I32 L34** M35 **V40**	various folds, including AD with down types IIIa and IIIb;^51^ brain-extracted ab42 fibril type II; type A (ν-shaped);^52^ arctic mutation fold A^53^
C4	10.30	8OWD/1	3	2	E11 **D23** A30 **I32** **V36** **V39** **V40**	lipidic Aβ1–40 polymorphs L2 and L3 ^50^
C5	9.77	8KF4/1	6	3	**L17** F19 **F20** N27 **A30** I31 I32 L34 **V39 A42**	brain-extracted Aβ1–42 fibril type I;^54^ murine Arctic mutation^55^
C6	20.11	9K0E/4	4	5	V24 N27 **K28** I32 **V39 V40 I41 A42**	Aβ1–42–4b polymorphs;^56^ Type B (υ-shaped)^52^
C7	18.92	8BFA/2	2	3	**Q15 D23** V24 **S26 N27** K28 **I31** M35	murine, arctic mutation fold B; Dutch + Iowa mutation polymorphs (DI1, DI2, DI3)
**TDP-43**						
C1	11.09	6N37/1	2	3	M311 **Q331** M336 L340 **N353 Q354 Q356 N358** Q360	dagger-shaped fold, SegA-sym and SegA-asym polymorphs^57^
C2	5.60	6N3C/3	0	0	F289 **A297 N301** M307 **N312 F313** F316 **I318**	R-shaped fold SegB-polymorph^57^
C3	-	7KWZ/1	14	10	F289 **A321** M323 Q331 **Q346** Y374 **S377 W385 S393**	a single in vitro structure of the entire low complexity region^58^
C4	12.90	8CGH/1	1	8	N302 **Q303 N306** M323 Q327 A328 **Q331 S332** Q344 **M359**	type A FTLD-TDP fold^59^
C5	15.03	6N3A/4	5	3	**N285** L299 N301 M307 **I318 P320 A324** Q331 M337 **W334**	dagger-shaped fold, SegA-slow polymorph;^57^ and ALS type B FTLD-TDP fold^60^
C6	1.97	8QXB/3	4	1	**M307 F316** P320 Q331 **M322** **M336** M337 **M339**	morphologies 1a, 1b and 2 ^61^
**IAPP**						
C1	13.98	8AZ3/2	3	1	**A8** ***F*23** **I26** L27 N31 **V32 N35**	C-fold (S20A mutant),^62^ present in polymorphs TW1, TW4 ^63^
C2	11.20	8QJ1/1	1	5	C2 **L12 F15** *F* **23** **I26 T36** Y37	polymorph TW2, symmetric fibril;^63^ A25P mutant single 3-fold rotation symmetry^64^
C3	8.81	7YL7/2	1	1	T6 **A8 L16 S20 N22 I26** N35 Y37	ζ-shaped protofilaments: all polymorphs of SF TF-types 1–3 ^65^
C4	3.96	9GZX/1	10	2	**S20 I26 S28 T36** T30 **Y37**	L-shaped chains in both control and doxazosin-bound polymorphs^66^
C5	13.36	8AZ7/4	0	0	L16 **N22 I26** **V32** **N35** **Y37**	J-fold (S20A mutant)^62^
C6	10.13	8AZ4/2	3	4	**F15 V17** S19 **I26 L27** N22 **T30**	P-fold and L-fold (S20A mutant);^62^ present in polymorph TW1 ^63^
C7	11.04	8AZ3/3	2	2	L12 **N22 A25 I26 L27 Y37**	U-fold (S20A mutant);^62^ plateau-phase fibril polymorph 4PF-LJ
C8	14.16	8R4I/1	0	5	C2 **Q10** S19 **N22** A25 L2**7** **Y37**	polymorph TW3; double S-shaped symmetric fibril present in polymorph TW4;^63^ polymorph PM1 ^67^
**TMEM106B**						
C1	2.31	8 × 5H/1	9	7	Y125 **Y132** T141 A179 L190 **I195 V199 H222** Q242 **Q245**	singlet polymorph II^35^
C2	3.00	7QWG/1	15	12	**V123 Y132** T141 **N154** I165 S218 **T235** Q242 **I243**	singlet polymorphs IIIa and IIIb^35^
C3	2.86	7QVC/1	2		**V123 Y132** T141 I165 **Q170** S218 **N223** F237 Q242 **I243**	singlet polymorph I, doublet polymorphs I and II^35^
**TTR**						
C1	1.96	8E7*I*/1	0	4	N27 **A29 Y69** W79 F87 **D99** L111 **P113 Y114**	cardiac ATTRwt fold^68^
C2	1.79	8PKF/1	4	6	N27 Y69 **V71 W79** F87 **F95** **L110** L11**1** **S115**	closed gate polymorph^24^
C3	1.61	8E7*E*/1	0	5	**S23** N27 Y69 **V71** W79 F87 **L110** L111 **Y116**	absent gate and broken gate polymorphs^24^
C4	-	8TDO/1	16	8	**S23** N27 E62 Y69 **V71** F87 **L110 L111 Y116**	open gate polymorph^24^
C5	0.13	7OB4/1	10	7	N27 **L58** Y69 **F87** **A91** **L110** L111 **P113**	blocking gate polymorph;^69^ reviewed in^24^

a RMSD_mod_ variance within clusters indicates structural homogeneity. Medoid structures (PDB code/protofilament number) represent the fold or similar folds. Signature residues were selected for precise steric fit and interface embedding (highest Sc and SDi values; column 6). Overall packing tightness is quantified by the number of residues with >90% of maximum Sc and SDi values (columns 4–5).

In summary, amyloid fibrils exhibit polymorphism across multiple structural levels, from 2D-folds to protofilament organization and overall fibril morphology. Our RMSD_mod_-based clustering captures this diversity, allowing systematic identification of representative folds, quantification of structural variability, and recognition of less populated or divergent polymorph families. While some proteins, such as TTR, display minimal variation, others, including Tau, α-syn, and Aβ, form highly diverse clusters often linked to distinct pathological contexts. This approach provides a robust, structure-driven framework for exploring fibril organization and understanding how specific sequence segments contribute to polymorphic outcomes.

### 2.6 Identification of Structural Hot-Spots Characteristic to the 2D-Folds

Formation of β-sheet interfaces via solvent exclusion is a key step in fibril assembly, generating nanostructures resistant to dissociation and degradation. Assigning local interface descriptors (Sc, SDi, and Ab) to each residue enables precise characterization of their contribution to tightly packed assemblies. Here, we visualized these descriptor values as heatmaps for individual protofilaments and cluster averages, allowing a fingerprint-like comparison between 2D-folds (Figures S10–S16). To identify primary sites of solvent exclusion and highlight cluster-specific patterns, we focused on residues with the highest Sc and SDi values, as Ab is strongly dependent on side chain size (Table 2). Interestingly, we found that signature residues, those with the most precise steric fit and deep embedding within the interface, differ even between structurally similar clusters, e.g., modestly different TTR clusters.

Amyloidogenic regions of the sequence are represented among signature residues, but their appearance varies by cluster. In case of Tau filaments (Figure S10), for example, the 299–310 segment overlaps only with the signature residues of cluster ***C3***. Other segments dominate different clusters: 357–368 in ***C1*** and ***C5*** and residues beyond 390 in ***C2*** and ***C4***. Signature residues can have distinct roles. In Aβ (Figure S11), the Lys16–Ala21 segment, a known core amyloidogenic region,^70^ appears in clusters ***C1***, ***C4***, and ***C5***, whereas Asn27 (except ***C4***) and Ile32 (except ***C2*** and ***C7***) are signature residues across nearly all clusters. This suggests that initial oligomers and mature polymorphs might utilize different interaction networks. A recent temperature replica exchange molecular dynamics and ensemble self-docking study of Aβ conformers^71^ showed that regions 1–8, 16–24, 28–36, and 37–42 are preferred to be involved in dimerization interfaces. Though the transiently formed β-sheets in this highly dynamic state of Aβ are mostly intramolecular, it is interesting to compare these association zones with those of protofilament structures. These four regions highly overlap with the hot-spot residues, with region 28–36 being represented in all clusters. Region 16–24 is also a major site of burying molecular interfaces, though in some clusters, residues at its C-terminal end are hot-spot residues. This suggests that regions 16–24 and 28–36 are general sites of molecular aggregation, independent of amyloid-forming conditions, mutations, etc., while the other two zones depend more on mutations, post-translational modifications (PTMs), and other amyloid-forming conditions.

For α-syn (Figure S12), three segments contribute to amyloid formation: P1 (36–42), P2 (45–57), and 71–82 in the NAC domain.^72^ The P1 segment involves signature residues in clusters ***C2*** and ***C5*** only, whereas one or two residues of P2 are signature residues in all clusters, consistent with its greater importance. The hydrophobic NAC segment contains multiple signature residues and a common residue, V74, present in five of the seven clusters.

Clusters were also evaluated for overall packing tightness using the top 10% Sc and SDi residues for each protein type (Table 2, columns 4–5). In Tau filaments, clusters ***C2*** and ***C4*** contain the most superior local interfaces. While ***C4*** is homogeneous (J-fold protofilaments), ***C2*** is structurally diverse, encompassing Pick, in vitro GGT, and unique folds. A detailed analysis of interfaces and topology is presented for Aβ protofilaments in Section 2.8, while corresponding analyses for the six other polypeptides are shown in Figures S10–S16.

In summary, analysis of 2D-fold interfaces across diverse amyloid fibrils highlights the critical role of β-sheet formation and solvent exclusion in the formation of distinctive fibril nanostructures. Local interface descriptors, steric fit (Sc) and interdigitation (SDi), identify cluster-specific signature residues that define the tightest-packed regions, or “hot-spots,” of each 2D-fold. While amyloidogenic segments contribute, signature residues do not always coincide with these regions, indicating that initial oligomers and mature polymorphs rely on distinct interaction networks. Overall, the location and density of these tightly packed interfaces vary across protein types and polymorphs, providing a structural basis for most compacted fibril regions and a framework for targeted modulation of amyloid assemblies.

### 2.7 Hierarchy of 2D-folds Shown for Aβ Fibrils

Aβ fibrils exhibit a remarkably diverse structural landscape, despite consisting of only 42 amino acids. Furthermore, many of its APR segments have been crystallized^17,70^ that also display polymorphism. Aβ has a high number of ex vivo structures determined, making it an interesting system for detailed analysis of structural polymorphism.

We identified 7 major clusters (***C1***–***C7***) (Figure 5) among Aβ fibril structures. The ***C2*** cluster contains straight chains resembling class 1 APR crystals, with a 2-fold symmetry axis corresponding to the fibril axis. Together with ***C1*** and ***C6***, these clusters form a subgroup characterized by main zipper regions between two protofilaments. The *N*-terminal conformers differ subtly, as in ***C1*** the chain adopts a U-shape, whereas in ***C6***, the backbone forms a wave-like bend, contrasting with the sharp turns observed in other clusters.

**Figure 5:**
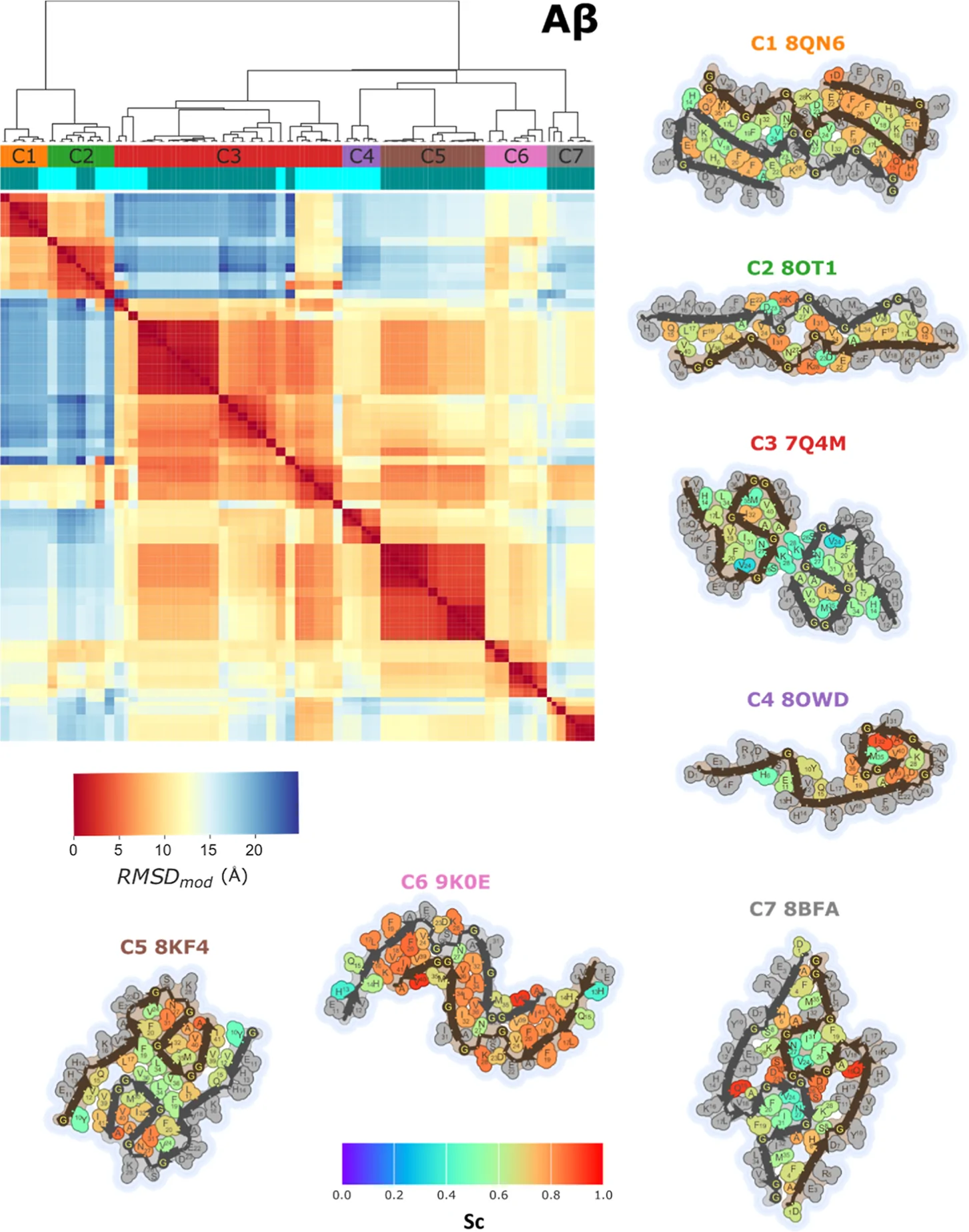
Clustering of Aβ protofilaments represented as the heatmap of RMSD_mod_. The dendrogram shows relative variance. Seven clusters are marked by the first colored row ***C1***: orange, ***C2***: green, ***C3***: red, ***C4***: purple, ***C5***: brown, ***C6***: pink, and ***C7***: gray. The second row indicates sample source (cyan: in vitro and teal: ex vivo). The cluster medoid of each group is visualized using the Amyloid Atlas Illustrator,^12^ colored by Sc values (blue–red: 0–1 and gray: no local interface). (High-resolution figures for different proteins are shown in Figures S3–S9 and in the extended material).

Another subgroup shows extended interprotofilament zipper regions, dominated by hydrophobic residues. These can be mapped to previously defined folds: fold type I corresponds to ***C5***, while fold types II and III form subclusters of ***C3***.^51,54^ These clusters share an S-shaped conformation from residue 24 onward; in ***C4***, a similar bend occurs at the C-terminus (local interface Ile32, Met35, Val40). ***C4*** contains Aβ40, and the absence of two residues enables the chain to curve differently, fully burying the C-terminus. Conformational similarity among ***C3***, ***C4***, and ***C5*** is reflected by smaller RMSD_mod_ distances in the clustering heatmap (light orange square, Figure 5).

Clustering also reflects mutation and complex/PTM effects on 2D-folds. ***C4*** includes lipid-induced fibrils (single or double protofilaments) with curled C-termini and surrounding unresolved lipids.^50^ Mutations at positions 22–23 (Arctic, Dutch, and Iowa mutations)^73^ enable a new fold (***C7***), where the middle of the chain is most buried and bends are less sharp, similar to ***C6***.

Cluster-averaged descriptors highlight structural hot spots and loosely packed regions (Figures 6 and S11). The Ab heatmap (Figure 6d) identifies tightly packed regions: segments 16–21 in clusters ***C1*** and ***C5*** and 29–40 in clusters ***C3*** and ***C5***, corresponding to APR regions.^73,74^ The largest local interfaces are in ***C1*** (Figure 6c), where both faces of the ^16^KLVFFAE^22^ motif are buried. Sc values are generally high for Aβ (Figure 3b), particularly in clusters ***C2***, ***C4, C5***, and ***C6*** (Figure 6a). ***C2*** and ***C6*** show a long and tight interface similar to those found in APR structures. ***C1*** also shows a long interface at the center of the structure, but the local interface is formed by the other face, where the local interface of Ile32 is less tightly packed than Ile31 in ***C2***. ***C4*** structures, though partially buried (Figure 5c,d), show high local Sc.

**Figure 6:**
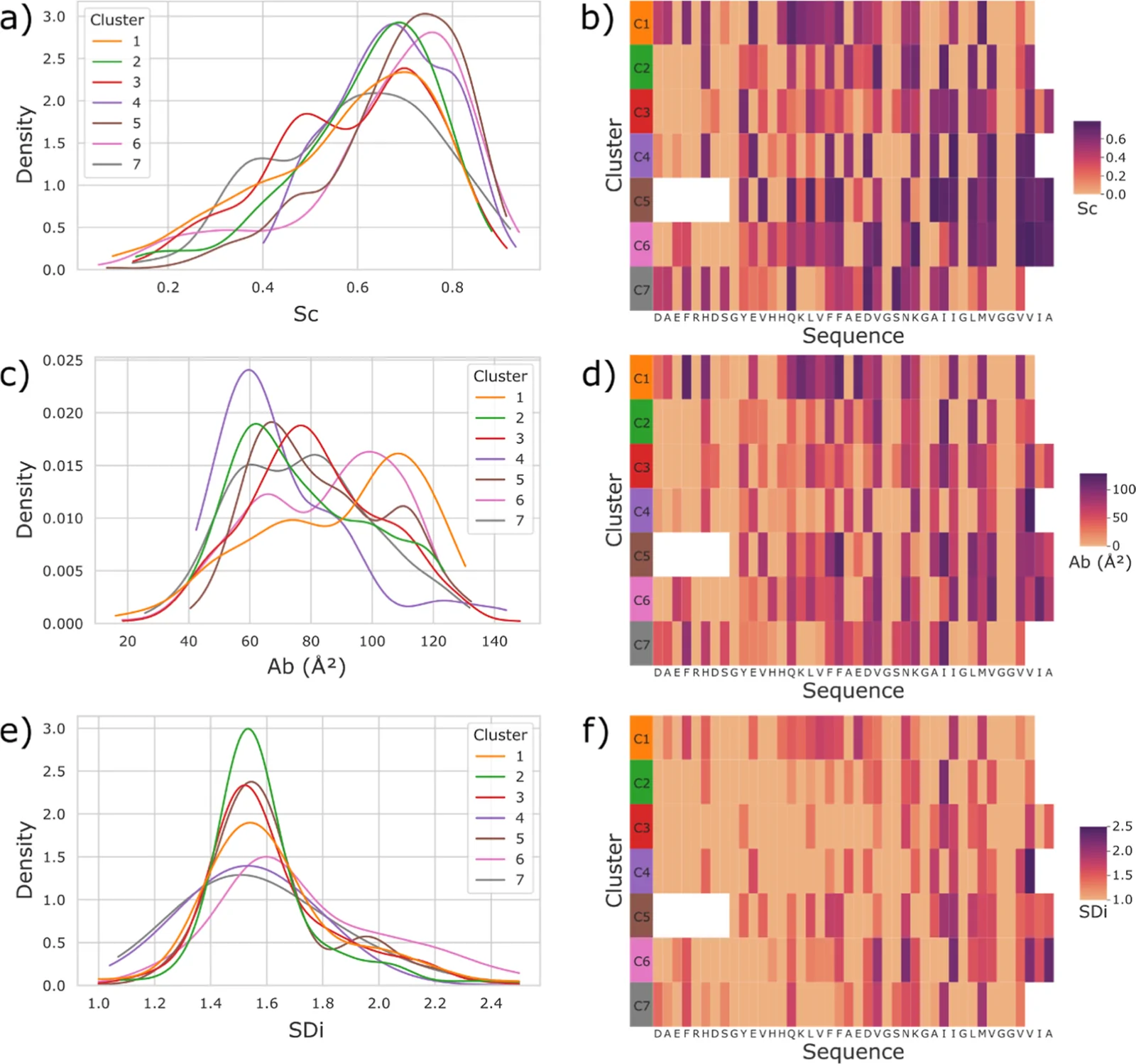
Summarizes Aβ descriptor values by cluster. (a,c,e) Kernel density distributions of Sc, Ab, and SDi. (b,d,f) Cluster-averaged heatmaps plotted against the amino acid sequence. These visualizations capture cluster-specific hot-spots and loose regions, clarifying the structural hierarchy of Aβ 2D-folds. (High-resolution figures for different proteins are shown in Figures S10–S16 and in the extended material).

SDi distributions are consistent across the clusters (Figure 6e). High SDi values are rare but are position-specific. The APR ^30^AIIGL^34^ segment shows an elevated SDi, notably Ile31 (Figure 6f). Bends or junctions generate high SDi; e.g., cluster ***C4*** shows the largest SDi around Val40, where the chain curls around the C-terminus. ***C2***, lacking turns (Figure 5), displays the narrowest SDi distribution (Figure 6e).

APR regions and heatmaps also illuminate mutation effects on Aβ. Mutations alter APR interfaces, impeding amyloid formation.^73,75^ Gatekeeper residues like Glu22 typically reside on solvent-exposed fibril surfaces, rarely contributing to zippers (Figure 5). It is only cluster ***C1***, where it forms the edge of the main zipper region, which is formed by the N-terminal segment in ***C1***. The importance of this segment in the 2D-fold of ***C1*** is also reflected by the accumulation of hot-spot residues in this segment (Figure S17). Interestingly, the structure possessing a mutation in this region (D7N Tottori mutation, PDB id: 9M5P) belongs to another cluster where the segment involving the mutation is disordered; thus, in this structure, the mutation does not have a major role in forming the most compacted part of the fibril.

Structures including mutation of Glu22 appear in Clusters ***C2***, ***C3***, ***C5***, and ***C7***, but not in ***C1***, indicating their different types of involvement in the interaction patterns. Glu22 is solvent-exposed in clusters ***C2*** and ***C4***, indicating that in these cases the E22G mutation could cause a difference in solubility of the aggregate without modifying the 2D-fold. In ***C3***, ***C5***, and ***C7***, there are examples where Glu22 is near short-polar interprotofilament connections; thus, its mutation could influence the arrangement of protofilaments. The mutation pair E22Q + D23N is present in cluster ***C7***, where it causes a tighter fit of the zipper but also diminishes interprotofilament interactions (PDB structures 8OLG, 8OLQ, and 8OLN, additional Supporting Information).

The Arctic mutation (E22G) extends local interfaces, forming complementary contacts in cluster ***C7*** versus those in other clusters (Figure 6b,d,f). The effect of the enhanced aggregation can be counteracted by another mutation:^76^ I31E. Ile31 is the most buried hot-spot residue with high SDi in ***C7***; its mutation disrupts structures belonging to not just the ***C7*** fold but also other clusters.

In summary, Aβ fibrils display a hierarchical landscape of 2D-folds, organized into seven major clusters (***C1***–***C7***). Clustering distinguishes subgroups by protofilament zipper regions, backbone curvature, and segment burial, reflecting both inherent sequence features and mutation or PTM effects. Cluster-averaged interface descriptors (Sc, SDi, and Ab) reveal signature hot-spots, highlighting residues and regions that drive tight packing. While amyloid-prone regions are often part of these hot-spots, key buried residues and interfacial topology vary across clusters, illustrating that polymorphic 2D-folds deploy distinct interaction networks. Mutations such as Arctic E22G modulate these interfaces, demonstrating the sensitivity of fold formation to sequence changes. Together, this analysis provides a comprehensive structural hierarchy for Aβ fibrils, linking cluster-level topology to residue-specific packing and polymorphism.

### 2.8 Characteristics of the Sites of Chemical Variations

Steps of amyloid formation, such as unfolding of protein, formation of oligomers, fibril elongation, secondary nucleation, and maturation to divergent polymorphic forms,^77^ depend on many factors, some of them having amplifying, while others inhibiting roles. These can be a complex system of tissue/solution components acting as cofactors, with mutations or PTMs causing conformational transitions or influencing processing rates of the polypeptide or their interactions. Some effectors thus can directly influence the amyloidogenicity of a certain polypeptide, while others indirectly modify protein/protein interactions or processing). Characterizing the relationships of these effectors and the most aggregated sites of the zippers of filaments, we chose examples where amyloidogenicity and other effects are distinctively studied.

In the previous section, we exposed the relationships of mutation sites and the zipper regions of Aβ within the database. There are several other mutation sites affecting amyloidogenicity of Aβ, with no structural data. These mutations, as described by Seuma et al.,^73^ are shown relative to the hot-spot residues (Figure S17). The hot-spot residues are accumulated in the longest zipper regions (segments 15–22 in cluster ***C1***; and segments of the C-terminal region is other clusters —both being APR regions). Most of the amyloidogenic mutations affect the most aggregated local environments within these sections for only one or two clusters, indicating that none of these mutations are of universal effect for all 2D-folds. There are some mutations with “protecting” roles, two near the N-terminus, which (based on the hot-spot residues) seem to be of less importance in the interaction patterns of matured fibril structures. In contrast, Asn27 is a hot-spot residue in all but one cluster, and its mutations inhibit amyloid formation. Asn27 is involved in kinks forming local interfaces with hydrophobic residues (Leu23, Ile31, Ile32, or Leu23+ Ile31, depending on the cluster), thus having an important structural role in burying these hydrophobic residues and also in folding the backbones.

For Tau, alternative splicing results in different isoforms containing 0–2N-terminal inserts and 3 or 4 microtubule-binding repeats (3R and 4R isoforms)—where the disease-specific presence or absence of the second (R2) repeat results in very different 2D-folds: the ***C1*** and ***C3*** clusters contain it, while ***C4*** and ***C5*** do not contain this repeat in their ordered cores. For analyzing how the effect of modifications at individual spots is reflected by the descriptors of the zippers, we chose cluster ***C5*** containing 3R/4R polymorphs. Tau’s PTM patterns in various pathologies and interactions with cofactors were extensively studied.^78,79^ Moreover, the necessity of anionic cofactors for stabilizing amyloids was also reported.^80^ Various PTMs described are also present in the cryo-EM maps, but these structural elements are often heterogeneous or disordered and not built in the cryo-EM maps. These are uniformly handled in our database: the nonprotein type groups are omitted, so they appear as cavities and small channels in the zipper regions. On one hand, this is a limitation of our method, as some basic segments of the zipper regions are not shown; on the other hand, this can be a tool to find sites of PTMs by comparing 2D-folds within the same cluster. Hot-spot residues and pathogenic mutation sites,^81^ as well as a disease-specific selection of PTM sites^82^ are shown in Figure S18. AD folds (AD-PHF and AD-SF) and CTE folds I and II all belong to cluster ***C5*** possessing similar, C-shape folds. The major difference is that in AD polymorphs Ser356 may be phosphorylated, pointing outward, while in CTE folds, this residue is buried and Leu357 is pointing outward. This conformational difference is also related to the fact that there is a cavity inside the filament in the CTE folds filled by disordered cofactors. Due to the flipping of the Ser356-Leu357 segment, the Lys353 side chain forms a more compacted structural element, burying Leu357 in CTE folds; in contrast, it is loosely packed, making the Ser356 side chain accessible in AD folds. This difference of compactness could be related to the observed opposing effects of PTMs: Lys353 acetylation inhibits Ser356 phosphorylation.^83^ These differences are shown by the local interface data: residues lining the cavity of CTE with unmodelled density are characteristically shown to be not involved in local interfaces. The tighter packing of Lys353 and Leu357 in CTE folds is indicated by their higher Sc, Ab, and SDi values. It should be noted that bound ligands on the surface of the filaments do not cause any difference: they are not involved in the zipper regions. We suggest that, analogously, the database can be used to find yet unlocalized cofactors or PTM sites.

In case of α-syn, we analyzed those and PTMs, which affect its aggregation propensity itself^84,85^ (Figure S19, PTMs with known effects are shown). Hot-spot residues accumulate in the APR region for clusters ***C1***, ***C5***, and ***C6***, while the more aggregated region is present in the N-terminal region in clusters ***C3*** and ***C7***, hot-spot residues being scattered along the protein chain, indicating that the impact of chemical modifications can be different for different groups of 2D-folds. The majority of aggregation-promoting modifications are next to or between hot-spot residues—this pattern is typical to the zippers of the β-sheet regions: every other side chain contributes to the zipper, while those in between them may form another zipper in the folded structure or may be solvent-exposed. This arrangement suggests that those aggregation-promoting modifications affect solubility or enhance forming more solvent-excluded surface pairs without disrupting the original zipper region. On the contrary, aggregation-inhibiting modifications are often hot-spot residues too in one or more clusters; thus, their changes disrupt the zipper interface. In the APR region, only aggregation-inhibiting modifications were found. Clusters ***C1*** and ***C2*** contain only modified structures—in the case of ***C2***-type 2D-folds, the site for the phosphorylated Tyr39 is part of the hot-spot residues, while in ***C1***-type folds, Gly51 is at the edge of the hot-spot region. Some modified residues, like Ala53, have representatives in multiple clusters, indicating they are compatible with multiple 2D-folds. There are also few exceptions, like Thr81 and Glu83, for which PTM decreases aggregation propensity—these are next to hot-spot residues and, indeed, usually are turned to the solvent region; however, Thr81 can be involved in interprotofilament zippers, and Glu83 has a charge-compensating role in the vicinity of positively charged residues.

### 2.9 Maturation of Amyloids Increase Local Compactness in Different Ways

Time-resolved cryo-EM studies^40,62^ reveal that multiple polymorphs coexist during amyloid maturation, with shifts in their relative abundances and the emergence of new intermediates. In vitro filaments, forming over hours to weeks, and ex vivo filaments, forming over years to decades, provide contrasting time scales for the same protein type. Though both short- and long-time-scale processes depend on many variables like conditions, PTM profile, mutations, associated cofactors—and for ex vivo filaments, presence of other components of the tissue—we were curious if there is any general difference in the zipper characteristics of short-term filaments forming and transforming new intermediate structures and long-term ones, where the set of polymorphs is more constant because of the sizes of the aggregates. We investigated structural shifts in compactness and signature residues using two examples: Tau amyloid intermediates from time-resolved cryo-EM studies of paired helical filaments (PHF, associated with AD) and CTE-type fibrils,^40^ and long-term maturation effects in Aβ and α-syn structures comparing in vitro and ex vivo data sets.

For Tau amyloids,^40^ the first intermediate (FIA) consists of a stretched dimer of 15 residues from the APR region, with topology resembling class 1 APR oligopeptide crystals. FIA evolves into multiple folds, mainly forming fibrils with two protofilaments. Clustering of these intermediates (Figure 7a) shows two major clusters: one includes J-shaped folds corresponding to 180–240 min intermediates (equivalent to cluster ***C4*** in Table 2 and Figure S3), while the other contains C-shaped folds, representing 300–360 min intermediates and 720 min mature fibrils (equivalent to cluster ***C5*** in Table 2 and Figure S3).

**Figure 7:**
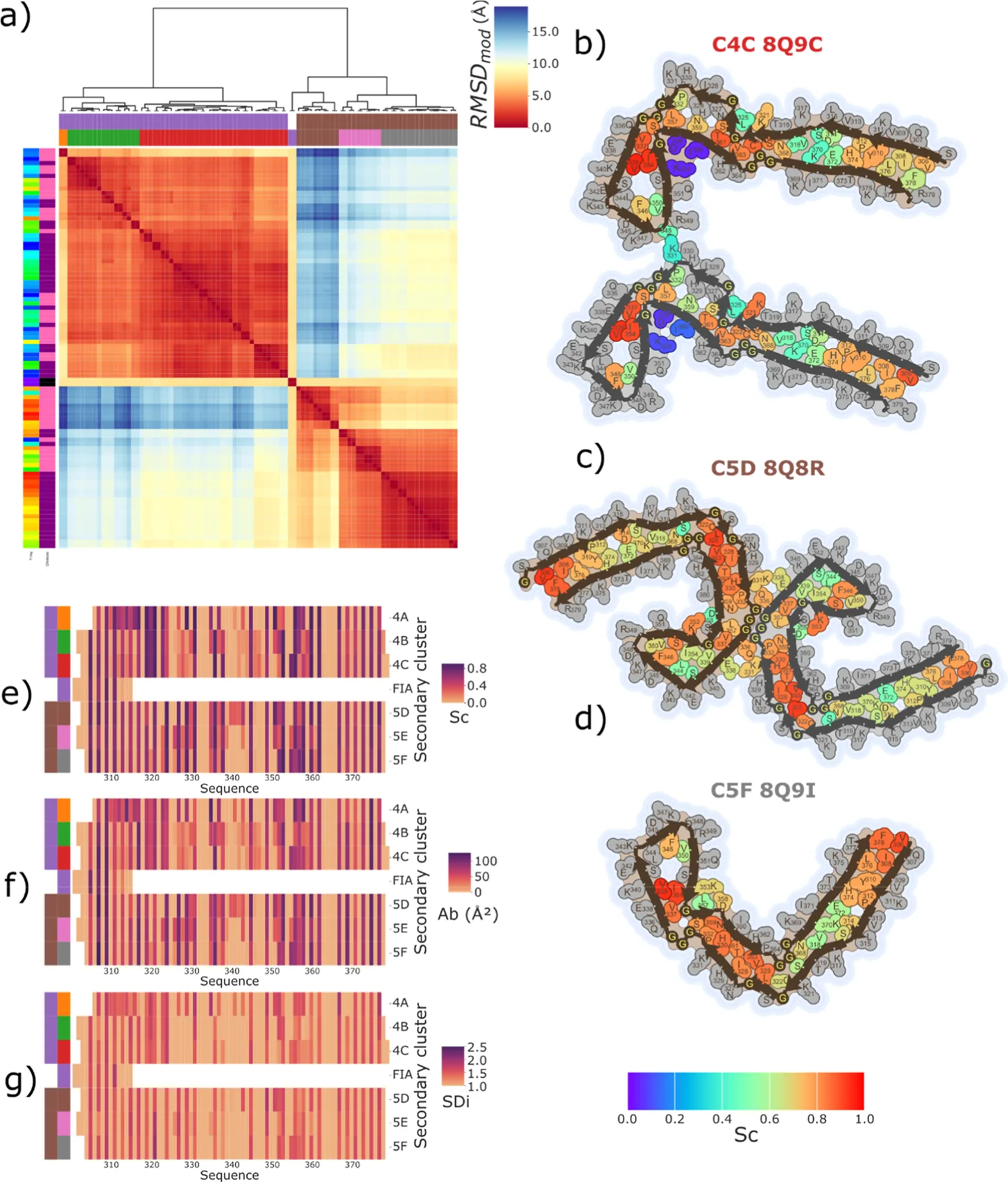
Changes in dominant 2D-folds and interaction hot-spots during Tau amyloid maturation. (a) Clustering results of time-resolved cryo-EM structures,^40^ with two major clusters (fist row: purple/***C4***; brown/***C5***) in full Tau clustering (Figure S2). Six subclusters are annotated (second colored row orange/***C4A***; green/***C4B***; red/***C4C***; purple: the single FIA structure belonging to cluster ***C2***; brown/***C5D***; and pink/***C5F***). The first colored column indicates fibril detection time (purple: 120, blue: 180, green: 240, yellow: 300, orange: 360, and red: 720 min). The second column indicates the experimental source: Alzheimer’s (pink) or CTE-modeling (pink/purple, black for both). (b–d) Medoid structures of largest subclusters of J-type and matured AD/CTE folds (PDB codes are 8Q9C, 8Q9I, and 8Q8R for subclusters ***C4C***, ***C5D***, and ***C5F***, respectively). (e–g) Local descriptors averaged for each subcluster shown as heatmaps (Ab, SDi, and Sc).

To refine characterization, we subdivided each large cluster using a 10% total variance criterion. FIA was classified as a separate cluster that shows low RMSD_mod_ values with each other protofilament (middle of Figure 7a). Up to 240 min, PHF and CTE reactions exhibit similar folds, with mixed structures in subclusters ***C4B***, ***C4C***, and ***C5E*** (Figure 7a). Beyond this point, divergence occurs: subclusters ***C5D*** and ***C5F*** represent CTE- and PHF-type filaments, respectively. Descriptor distributions indicate shifts during maturation, as local Ab values increase, reflecting tighter packing, while high Sc and SDi regions emerge with J-shaped filaments (***C4***), and their relative proportion slightly increases in later C-shaped filaments (***C5***). Heatmaps of Ab along the polypeptide sequence highlight burial of segment ^325^LGNIHH^330^ (Figure 7f), it contains 3 hot-spot residues (Table 2). In the new interface Sc is high, but edge residues (Ser320 and Cys322) show decreased Sc, indicating local loosening. The 325–345 region exhibits minimal intraprotofilament interfaces (Figure S10d,e,f), consistent with preformed interprotofilament binding sites. Subclusters ***C5D*** and ***C5F*** reveal distinct interprotofilament binding modes in CTE versus PHF filaments. SDi heatmaps identify unique sharp-turn features in subcluster ***C4A*** (Figure 7g) and differentiate PHF and CTE filaments at hot-spot residues Cys322 (***C5D***) and Val350 (***C5F***). Initial interface residues in FIA remain buried during maturation, serving as aggregation hot-spots despite being subsequently covered by C-terminal regions.

There are similar changes in another time-resolved cryo-EM experiment of IAPP.^62^ IAPP also evolves divergently; L and C conformers emerged from the first paired P-type fold (Figures S20 and 21). Interestingly, the increase in Ab values with maturation is predominantly caused by interactions of additional protofilaments, with the emergence of fibrils with 3 and 4 protofilaments (Figure S22). Inter-protofilament interactions in the early P fold (especially residues Asn21, Phe23, and Val32) have high Sc, yet in the later L type they are missing (Figures S20 and S22). However, the inward-facing residues in the bend region (15–28) have medium Sc, while in the L type, it has high. At the 22 weeks mark, these regions have lowered Sc and slightly elevated Ab, while new interactions emerge with high Sc at the outside-facing residues of the bend. C-type folds are bent the opposite way (Figure S21), possibly because there are many intermediates between them and the P-type folds. Interestingly, the additional protofilament lowers Sc in the contact region but elevates at the other side of the bend for this fold type (Figure S21).

Longer time-scale processes also distinguish in vitro and ex vivo filaments. Such long-time-scale effects are shown by Aβ and α-syn fibrils too: Ab increases are a clear marker of maturation, evident in distributions of in vitro versus ex vivo Aβ filaments (Figure S23a,b). SDi also shows a new local maximum at high values for ex vivo Aβ filaments (Figure S23c), showing an increase of interdigitation in the long term, leading to tighter folds. Another long time-scale effect can be observed in α-syn dimer fibrils as folds shift over decades in multiple system atrophy:^86^ 2D-folds represented by PDB structure 6XYO declines while 6XYP and 6XYQ predominate, representing a more matured form. These folds belong in the same cluster, as the change is restricted to the region of residues 70–100. This region adopts two new folds with larger Ab values but lower Sc values compared to the original fold (Figure S24).

In summary, our topology- and interface-based framework enables detailed analysis of amyloid maturation across both short- and long-term data sets. Tau intermediates reveal early J-shaped folds with persistent aggregation hot-spots that guide the formation of later C-shaped PHF and CTE filaments, reflecting shifts in interprotofilament contacts. Descriptor trends, Ab, Sc, and SDi, highlight local compaction, emerging sharp-turn motifs, and residues maintaining key structural roles, even as the global fold evolves. Comparisons of in vitro versus ex vivo Aβ and maturation of α-syn filaments show analogous maturation patterns; however, the available data is still insufficient to draw firm conclusions. These results demonstrate that our topologically grounded approach can capture both static and dynamic structural hierarchies, linking residue-level interface features to the evolving landscape of amyloid polymorphism.

## 3 Conclusions and Outlook

The structural complexity of amyloid fibrils presents a major challenge for understanding their formation, polymorphism, and maturation. While approaches describe per-residue energetic contributions to fibril stability, our approach might provide an alternative view when polymorphic states are of similar stability. Our topology- and interface-based framework allows recognition of common structural patterns, structural similarity (beyond backbone conformations), and the common characteristics of these 2D-folded structures. By defining local residue-level interfaces and quantifying shape complementarity (Sc), buried surface area (Ab), and surface detail index (SDi), our method captures the hierarchy of 2D-folds, identifies signature residues, and distinguishes tightly packed “hot-spots” from loosely packed regions across diverse amyloid types.

Comparison with structures of model systems based on canonical APR sequences highlights key differences: while APR crystals display narrow, highly ordered β-sheet interfaces, full-length cryo-EM fibrils exhibit extensive, meandering interfaces with bends, junctions, and variable curvature, possessing more aggregated regions at few spots. Quantifying the characteristics of the zipper regions shows that fibril structures are, in general, less tightly packed, indicated by the lower ratio of high Sc values, though they show tighter packing than APRs in a few spots, and they contain a large range of precisely to loosely fitting local interfaces.

Polymorphs collected in families by structure-based clustering often share a highly compacted region as their most similar 2D-fold element. The interface topologies for these collective clusters also contain distinct sets of tightly interacting residues—cores for solvent exclusion, characteristic to each cluster. Mapping of local characteristics of the zipper interfaces to polymorphic 2D-folds reveals how sequence features, mutations, and post-translational modifications modulate packing and protofilament interactions.

Importantly, our per-residue approach tracks maturation, capturing shifts in interprotofilament contacts, local compaction, and sharp-turn motifs, linking early oligomeric arrangements to fully matured fibrils. In the short-to-medium term evolvement of amyloids, the highly interdigitated regions tend to shift from aligned β-sheet-pairs to the kinked segments of the 2D-folds. Maturation leads to burying larger surfaces and reorganization of protofilaments forming later polymorphs, with ex vivo structures representing more compact assemblies—we hypothesize—that can be related to their higher resistance to enzymatic degradation.^34,87^ The framework thus unites static structural snapshots with dynamic, time-dependent changes, providing mechanistic insight into amyloid evolution.

Overall, interface hot-spots identified across polymorphs define residues and regions critical for aggregation to fibrils and also provide information on accessibility for possible therapeutic targeting. By directly linking residue-level topology to fibril polymorphism, our method provides a rigorous, experimentally grounded tool for dissecting amyloid architecture and its maturation across temporal and structural scales.

## 4 Methods

### 4.1 Extraction of Cryo-EM Amyloid Fibril Structures from the PDB

Amyloid fibril structures were manually extracted from the PDB^22^ and Amyloid Atlas.^12^ Analysis of structures determined by NMR methods revealed that extension procedure based on fitting backbone atoms of consecutive fibrils led to ambiguous long-range superstructure. Consequently, further analysis presented in this study was performed for structures obtained by cryo-EM. Only structures incompatible with our methods were filtered manually, if: (i) the deposited structure did not contain at least 3 chains for each protofilament as it is needed for additional chain generation. (ii) Their protofilaments contained a different number of chains, leading to an ambiguous extended structure, (iii) the chains from one protofilament contained a different number of atoms, (iv) they contained PTMs, or (v) a memory-allocation problem occurred during the SASA calculation of structures. A total of 543 structures were found that meet the criteria. Ex vivo samples (extracted from tissue) were categorized according to the Amyloid Atlas.^12^ This data set is mostly high-resolution (Figure S25): only 23 structures have a resolution worse than 4.0 Å, and merely 5 of them are worse than 4.5 Å. Also, it should be considered that the local resolution is very varied within the cryo-EM maps, and typically the highest resolution region is the rigid fibril core.^88^ Because our Sc, Ab, and SDi metrics specifically evaluate these deeply buried side chains, the calculations sample the most reliable and well-defined coordinate regions, effectively mitigating the impact of low-resolution surface artifacts. Thus, we did not apply resolution-based rejection criteria.

### 4.2 Structure Analysis Software and Automation

The structures were analyzed using PyMol^89^ (visualization, fibril generation, and structural alignment) and the following programs of the CCP4 package:^90^ SC^91–94^ (Sc, shape complementarity index calculation) and AREAIMOL^95,96^ (SASA, solvent-excluded surface area calculation). Based on our previous PyMol plugin and database ACW (Amyloid Coordinate Wizard),^21^ we created a plugin ACWF for amyloid fibril structures, its functions including representation of the current database elements and structural clusters, as well as analysis of new structures. It is capable of automatically extending the deposited fibrils, assigning the local interfaces, and calculating its descriptors. Results presented here were calculated using a fully automated protocol, except for the following steps: Extraction of structures from the PDB,^22^ filtering for applicability for calculations, and assignment of protofilaments, where the results were inspected for the need of manual corrections.

### 4.3 Clustering Algorithm

Clustering was performed only for protein types represented by at least 15 protofilament structures, ensuring sufficient sampling of structural variability (Table 1 and Figure 4). First PyMol’s “align” function was used with zero cycles of refinements to generate RMSD values. The middle chain of each protofilament was fitted. This function carries out sequence alignment prior to structural alignment, so the same regions of the polypeptide structures are compared between protofilaments (1 and 2). The *RMSD* values are modified (RMSD_mod_) using the proportion of the overlapping sequence length (length_1∩2_) to the total sequence length (length_1∪2_) of the two compared protofilaments scaled by the average RMSD as follows (eq 1)­

1 $${RMSD}_{\mathrm{mod}}=RMSD+\overset{®}{RMSD}\times \left(1-\frac{{length}_{1\cap 2}}{{length}_{1\cup 2}}\right)$$

RMSD_mod_ is considered as the distance between two structures in the agglomerative hierarchical clustering. It was carried out by the SciPy linkage algorithm using Ward’s method,^36,37^ which better accommodates clusters with differing homogeneity^97^ than average-based clustering methods. For example, in the case of Aβ clusters, different variances are present: cluster ***C5*** (Ab42 Type I + Arctic mutation fold) shows lower in-cluster variance (9.77 Å^2^) than cluster ***C6*** (Ab42-b polymorphs, 20.11 Å^2^; Table 2). Cluster limits were manually set to 30% of the total RMSD_mod_ range; this global value allows clusters to reflect structure-set properties for each protein type, also expressing the relationships between partially similar 2D-folds. For analysis of maturation effects, subclusters were also generated for the set of time-resolved cryo-EM structures where the dendrogram was cut at 10% too to reveal the divergent evolution of the maturation. Heatmap and dendrograms were generated by Seaborn.^98^

### 4.4 Extension of Fibrils from Deposited Coordinates in the PDB

Amyloid fibrils can be considered quasi-infinite structures. However, a repeating representation with periodic boundaries would not be compatible with SC and AREAIMOL. The deposited structures contain only a small part of the protofilaments, which should be extended to better stimulate quasi-infinite structures. For these extension calculations, the twist and rise parameters are deposited in the PDB in a majority of the cases. However, these values are occasionally rounded, and in rare cases, the sign is missing, which could cause errors in coordinate extensions based on them. The position of the fibril axis is not deposited in the PDB, but parameters of the bounding box are deposited in the EMDB, and the axes can be found in the middle. In some cases, however, the structure might have been centered or rotated during refinement, making the information unreliable. Therefore, we generated additional chains by repeatedly fitting the deposited chains to themselves.

The number of chains to be generated should be carefully chosen for avoiding the effect of finite fibrils on the descriptor values. One of them is the ‘edge effect’; that is, the molecular surfaces of the outermost chains of a finite filament are different, resulting in smaller contacting areas and different shapes of surfaces for them. Contacts may get overlooked due to the warping of the fibrils, where a peptide chain spans besides multiple chains, forming an interface with all of them (Figure 1f). Thus, the following algorithm was applied (Figure 8a): (1) along the fibril axis, the lowest and highest points of the van der Waals surface were determined, and their distance was calculated (this is the theoretical limit for the furthest possible interaction). (2) For avoiding the warping and edge effects for the middle chains of the extended filament, this distance is multiplied by 3 (Figure 8a, chains A, B, C or D, E, F) to ensure the coverage of interactions in both directions. (3) Then this distance is divided by the rise and the number of chains in the original protofilament. The result is rounded up to the nearest odd number; this value represents how many copies of the original structure are needed. (4) Extension of the deposited structure is carried out by repeatedly fitting Cα atoms of the first N-1 chains to the last N-1 chains within the protofilaments. The RMSD values of this shifting-and-fitting algorithm are less than 0.1 Å RMSD in 66% of the protofilaments, and there are only 15 cases where this value is larger than 0.3 Å.

**Figure 8:**
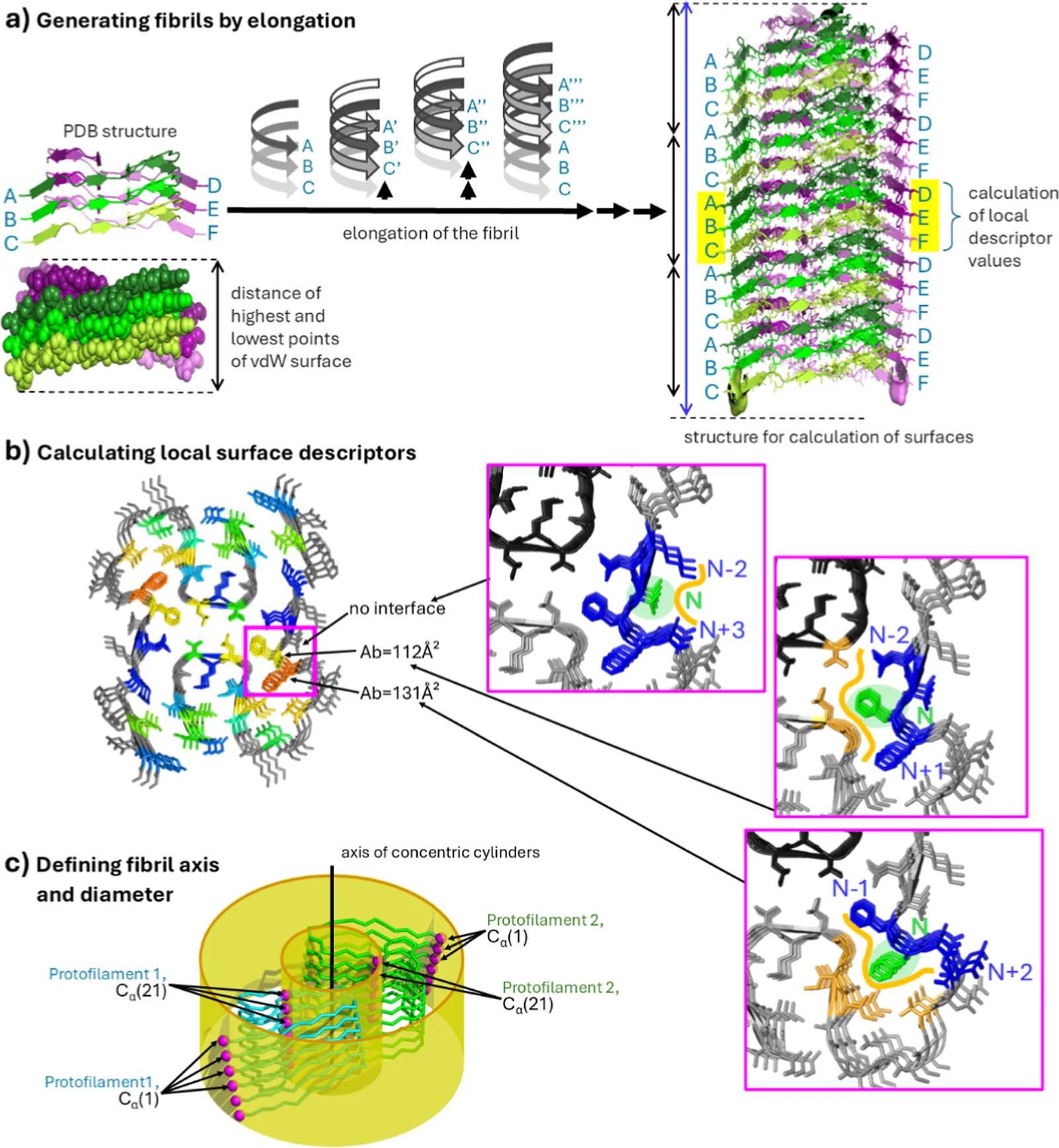
Steps of calculation of fibril descriptor quantities. (a) Elongation of the PDB structure for elimination of filament-truncation artifacts. The surfaces are generated for the whole structure, and local descriptor values of the middle chains are averaged (highlighted in yellow). (b) A sliding window method is used to calculate the descriptor values of the local interfaces of each residue (represented in rainbow colors, from low to high values: from blue to red). Figure insets show examples of assigning to consecutive residues (the N^th^ side chain is highlighted in green) their local interfaces (blue: segment N – 3 – N + 3, forming interface(s) with residues in yellow). (c) Method of calculating fibril axis and fibril diameter used in this study: fitting multiple coaxial cylinders to Cα atoms.

### 4.5 Calculation of Descriptors

To assign interfaces similar to those of peptide amyloid crystals, we applied a sliding window method (Figure 8b). For each side chain in the polypeptide sequence, a local interface(s) involving its neighboring residues in all filaments is explored, and the descriptor values are calculated to characterize the role of that residue in the 2D-fold of the polypeptide. The sliding window algorithm for defining local interfaces is as follows: (1) For the N^th^ residue, the contacting residues of its side chain are found (based on Areaimol calculation). (2) Then, the residues are separated into two groups: those with residue numbers between *N* – 3 and *N* + 3, together with the original residue, are assigned to the same polypeptide chain and form surface-A of the local interface; residues outside this residue range or in other protofilaments are assigned to the counteracting surface(s) (surface-B) of the local interface. Similar to our previous article,^21^ local interfaces are only accepted if at least 3 residues with their side chains are interacting. (3) The descriptor values Sc, Ab, and SDi are calculated similarly as described in^21^ for surfaces A and B and assigned to the *N* ^th^ residue. Sc is calculated for the whole surface; the Ab is reported as the average from the middle chains. Note, the definition of SDi includes a projection along the fibril axis (the axis of the local β-sheet in APR crystal structures). While this axis is well-defined in crystal structures, its direction changes with the twisting and the distance to the fibril axis. Thus, the axis was approximated for this calculation locally, for 3 chains at a time: the projection vector assigned to the *N* ^th^ residue is fitted to the Cα atoms of the *N* ^th^ residue of the middle polypeptide chain and those of the chains directly above and below it within the protofilament. Atomic coordinates are projected onto a perpendicular surface, and SDi is calculated as defined in our previous study.^21^ After calculating it for each of the 3 chains, the SDi values are averaged. KDE plots were generated using Seaborn.^98^ Hot-spot residues are those with the 5 highest cluster-averaged Sc or SDi values. This could be skewed by a structure with high values in a sequence segment unique within a cluster. To avoid residues being identified as hot-spots in regions where only one structure is ordered, these were dropped from hot-spot assignment unless the cluster contained 3 or fewer structures.

### 4.6 Calculation of the Diameter of the Filament

Was carried out by fitting concentric cylinders using the least-squares method to the Cα atoms after extension (Figure 8c). The position (*x*, *y*) of the fibril axis as well as the radius of the cylinders was refined. Each residue with the same sequence position across all chains was assigned a cylinder, resulting in a total of 2 + *N* variables, where *N* is the number of residues within the sequence. The diameter of the widest cylinder was considered the diameter of the fibril. Note, this method resulted in reasonable results for structures with RMSD of the chains within the protofilament <0.1 Å, so these are discussed here. Total warping was calculated as the distance between the projections of the lowest and highest Cα atoms of one chain along the fibril axis.

## Supplementary Material







## Data Availability

ACWF plugin for PyMol^89^ (including the database of the polypeptide fibril structures and resulting data of their analysis presented here) is made publicly available on GitHub: MateSulyokEiler/ACWF.

## Notes

High-resolution clustering heatmaps, cluster-averaged and unaveraged descriptor values for full local interfaces and interprotofilament local interfaces, and KDE distributions for each protein type analyzed (ZIP)
Clustering results and cluster-averaged local interface descriptors shown as heatmaps, comparisons for representative distributions of protofilament descriptors, and various fibril mutation and maturation effects on Sc, Ab, and SDi (PDF)
Data for all analyzed fibril structures, filament descriptor values, and clustering results (XLSX)

## List of Abbreviations

α-syn: α-synuclein
**Ab**: buried area of the molecular surface
**Aβ**: amyloid-β
**ACWF**: amyloid coordinate wizard for fibrils, a PyMol plugin to analyze cryo-EM amyloid structures, presented in this article
**AD**: Alzheimer’s disease
**APR**: aggregation prone region
**CTE**: chronic traumatic encephalopathy
**FIA**: first intermediate amyloid in TAU time-resolved cryo-EM experiment
**GGT**: globular glial tauopathy
**IAPP**: islet amyloid polypeptide
**LC**: immunoglobulin light chain
**PD**: Parkinson’s disease
**PDB**: Protein Data Bank
**PHF**: paired helical filament
**PTM**: post-translational modification
**SASA**: solvent accessible surface area
**Sc**: shape complementarity
**SDi**: surface detail index
**TDP-43**: transactive response DNA binding protein 43
**TMEM106B**: transmembrane protein 106B
**TTR**: transthyretin.
