# Supplementary material for: Structural Hierarchy and Maturation of Amyloid Fibrils Revealed by Interface Descriptor Analysis
Source: J Chem Inf Model. 2026 Aug 21;66(17):11471–90. doi: 10.1021/acs.jcim.6c01376 (PMC13580560; doi:10.1021/acs.jcim.6c01376)
Supplement: Supplementary file 1 [file ci6c01376_si_002.pdf]

Structural hierarchy and maturation of amyloid fibrils revealed by interface descriptor analysis

Máté Sulyok-Eiler[2,3], Veronika Harmat[2,4] and András Perczel[1,2,4*]*

[1] Medicinal Chemistry Research Group, HUN-REN Research Centre for Natural Sciences, H-1117 Budapest, Hungary
[2] Laboratory of Structural Chemistry and Biology, Institute of Chemistry, ELTE Eötvös Loránd University – Pázmány P. stny. 1/A, 1117 Budapest, Hungary
[3] Hevesy György PhD School of Chemistry, Institute of Chemistry, Eötvös Loránd University, Pázmány Péter stny . 1/A, Budapest, Hungary
[4] HUN-REN-ELTE Protein Modelling. Research Group, Hungarian Research Network, Pázmány P. stny. 1/A, 1117 Budapest, Hungary

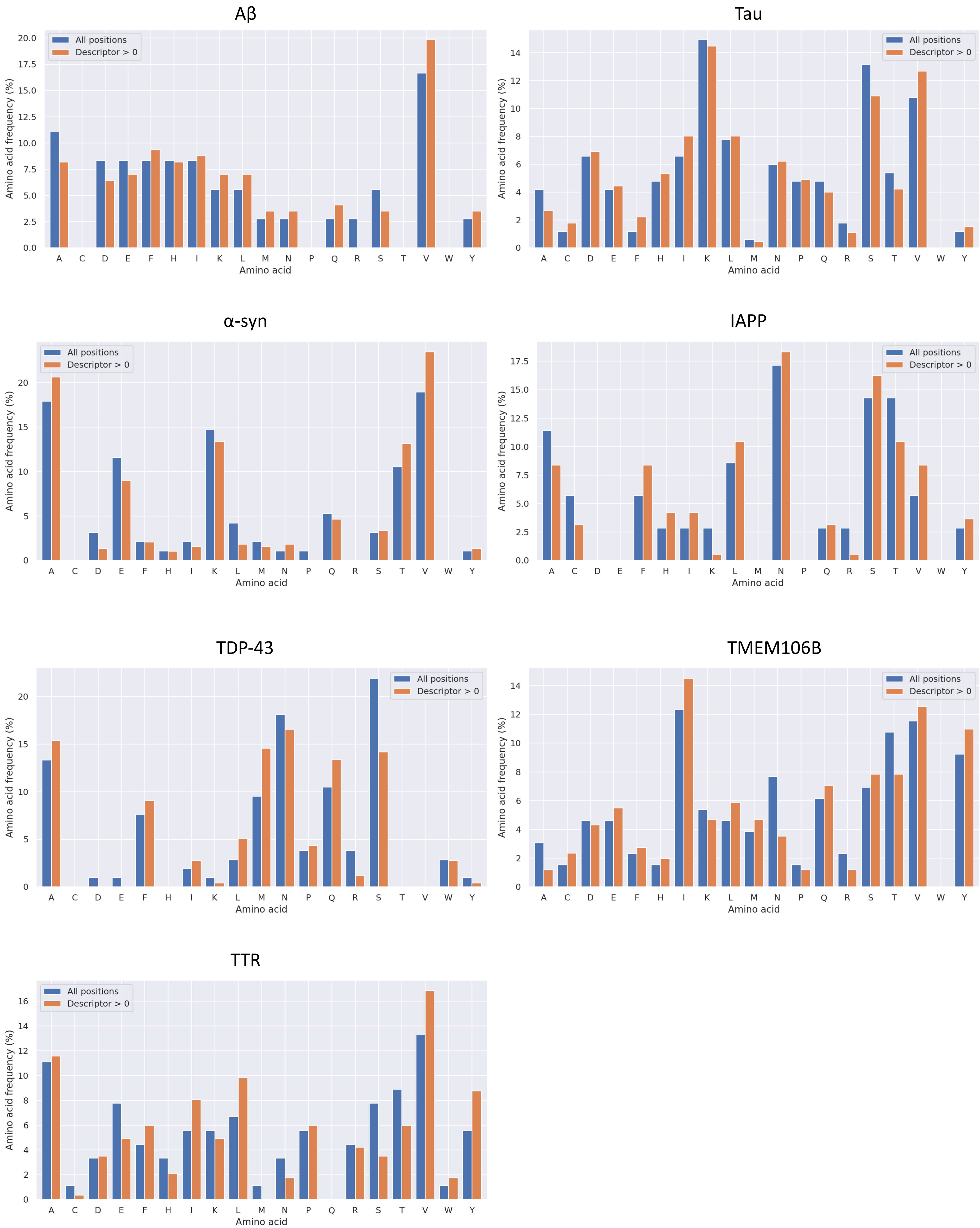

Supplementary Figure S1. Amino acid frequency in the different types of protein. Frequency is shown for the full sequence of the structure also for sidechain with local interface (descriptor >0)

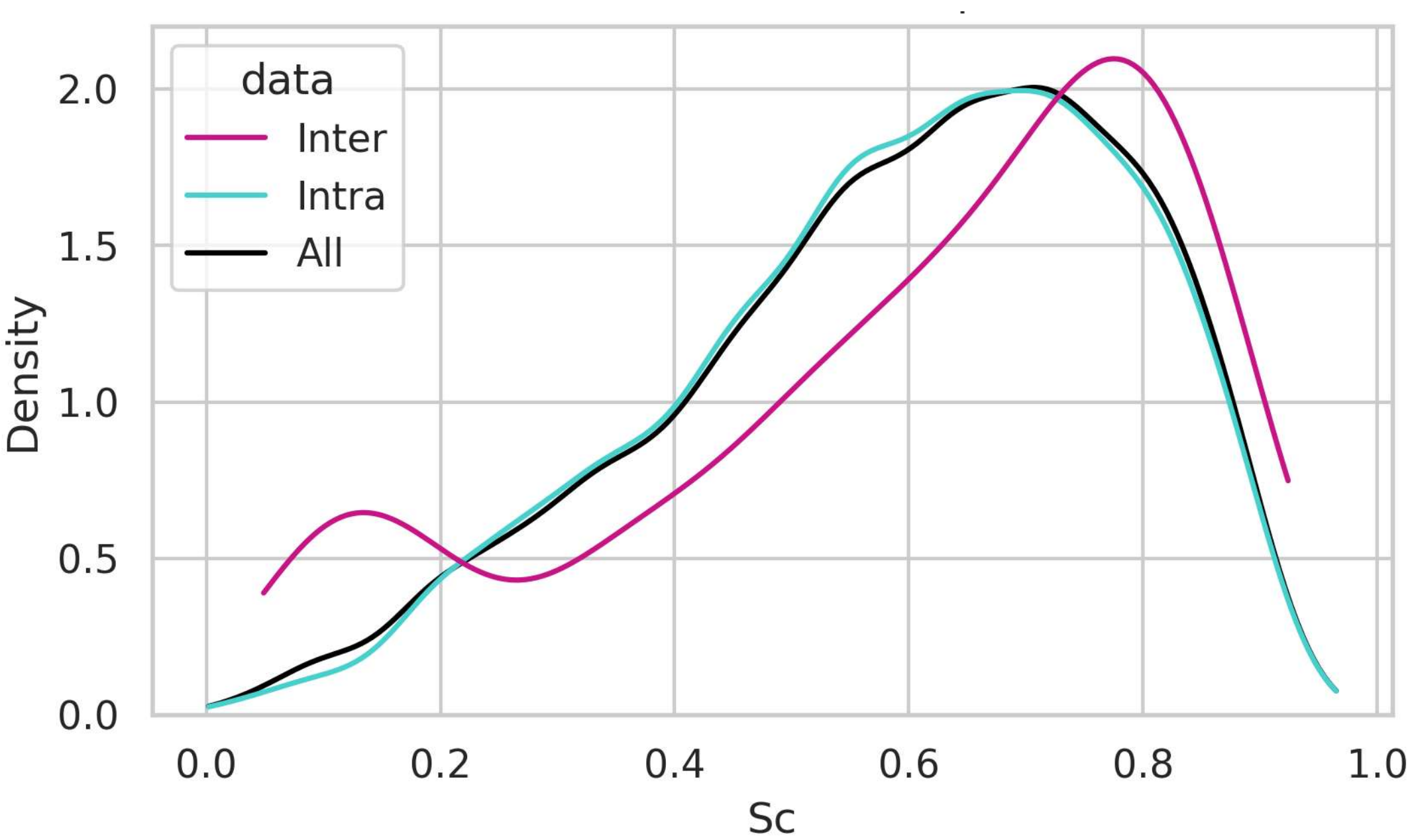

Supplementary Figure S2. Distributions of Sc values calculated for inter-protofilament, intra-protofilament and all interfaces in α-syn structures. Distributions are shown as kernel density plots.

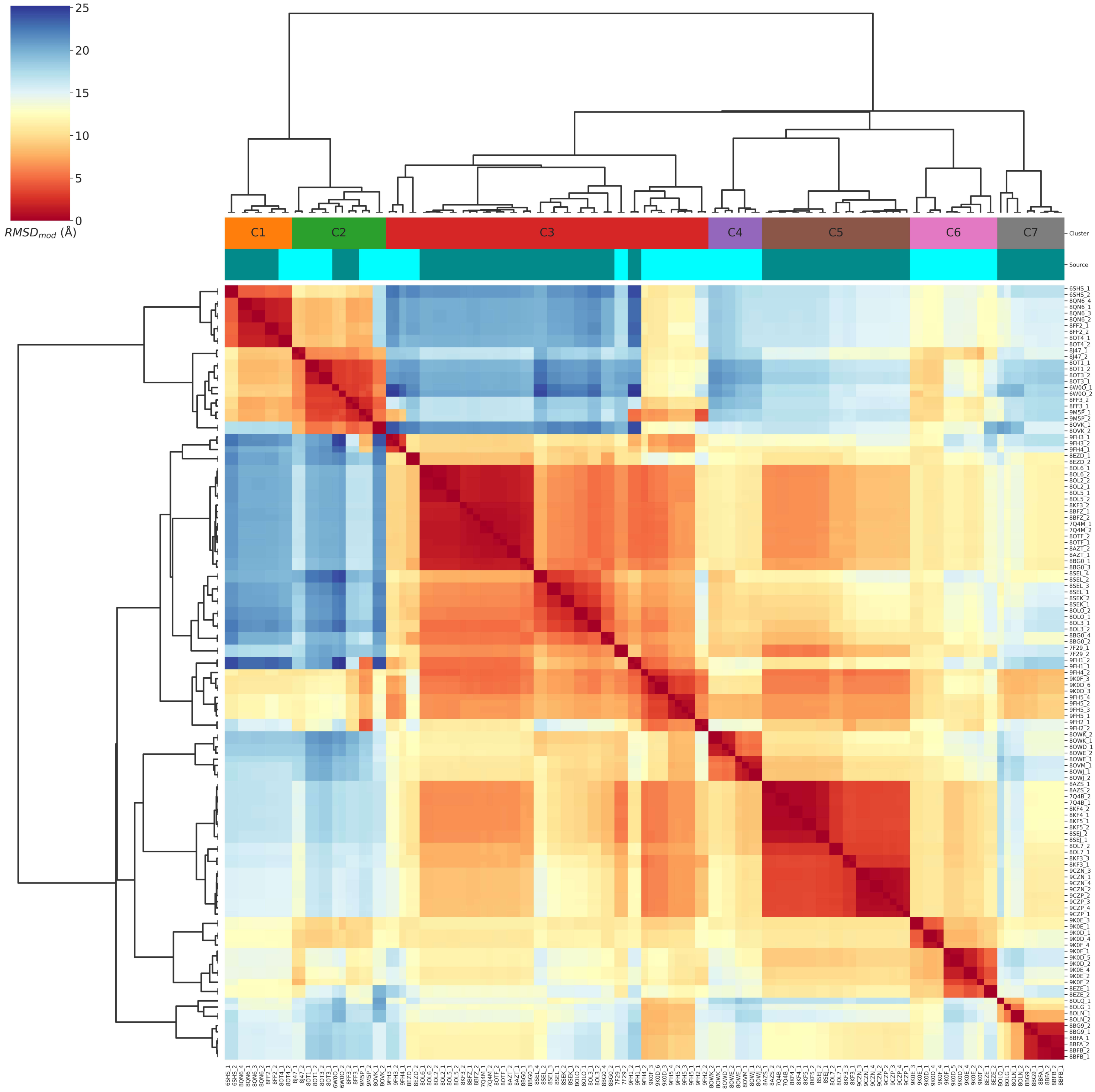

Supplementary Figure S3. Agglomerative clustering result of **Aβ** protofilaments, shown as a heat map of $RMSD_{mod}$ values. The dendrogram represents the relative variance in the clustering. Clusters (assigned with a limiting value of 30% ot the total $RMSD_{mod}$) are shown as the first colored row(color-coded as *C1*: orange, *C2*: green, *C3*: red, *C4*: purple, *C5*: brown, *C6*: pink, *C7*: grey) . The second colored row denotes the origin of the structure (cyan for in vitro, and dark cyan for ex vivo)

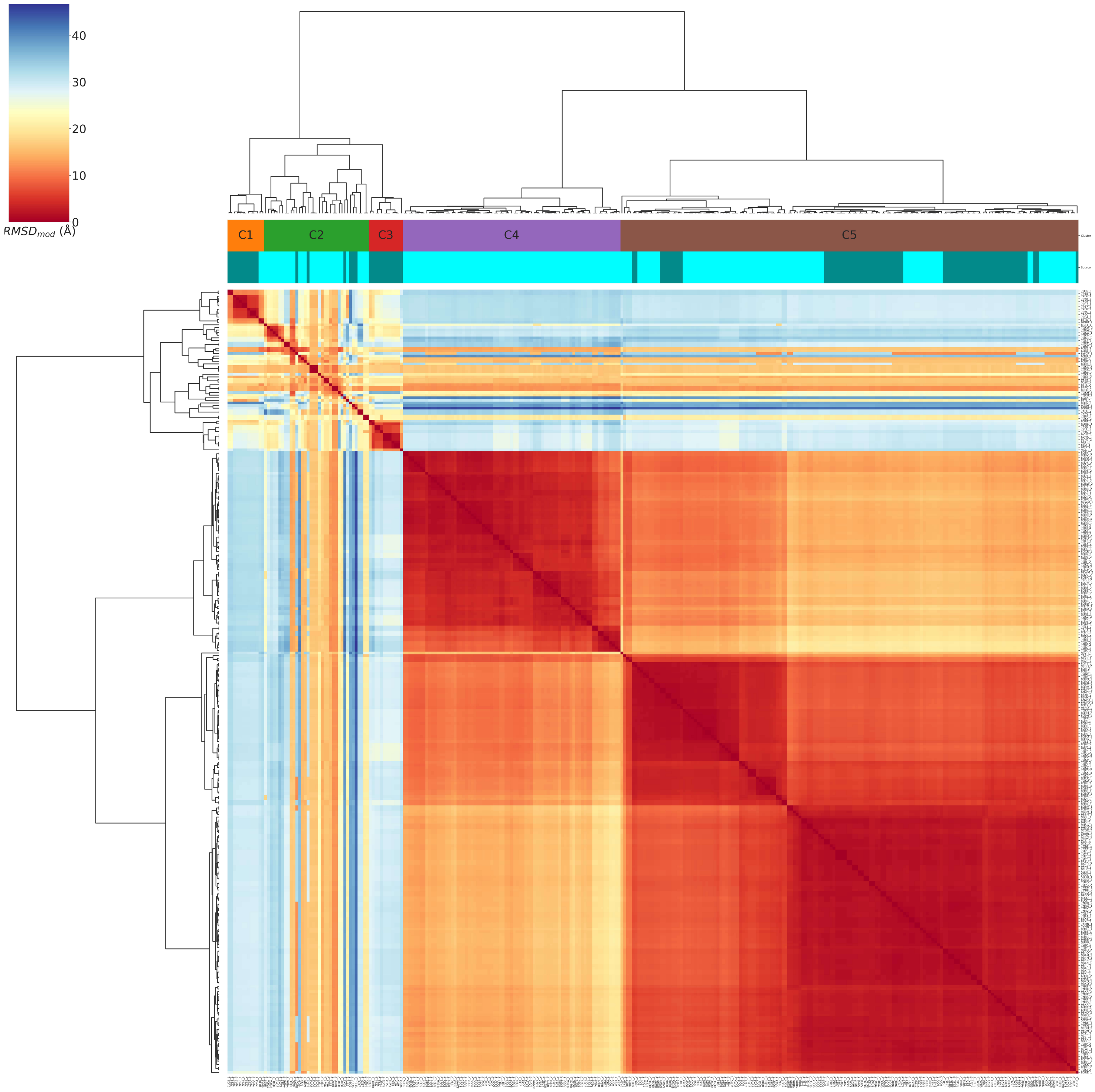

Supplementary Figure S4. Agglomerative clustering result of **TAU** protofilaments, shown as a heat map of $RMSD_{mod}$ values. The dendrogram represents the relative variance in the clustering. Clusters (assigned with a limiting value of 30% ot the total $RMSD_{mod}$) are shown as the first colored row(color-coded as *C1*: orange, *C2*: green, *C3*: red, *C4*: purple, *C5*: brown) . The second colored row denotes the origin of the structure (cyan for in vitro, and dark cyan for ex vivo)

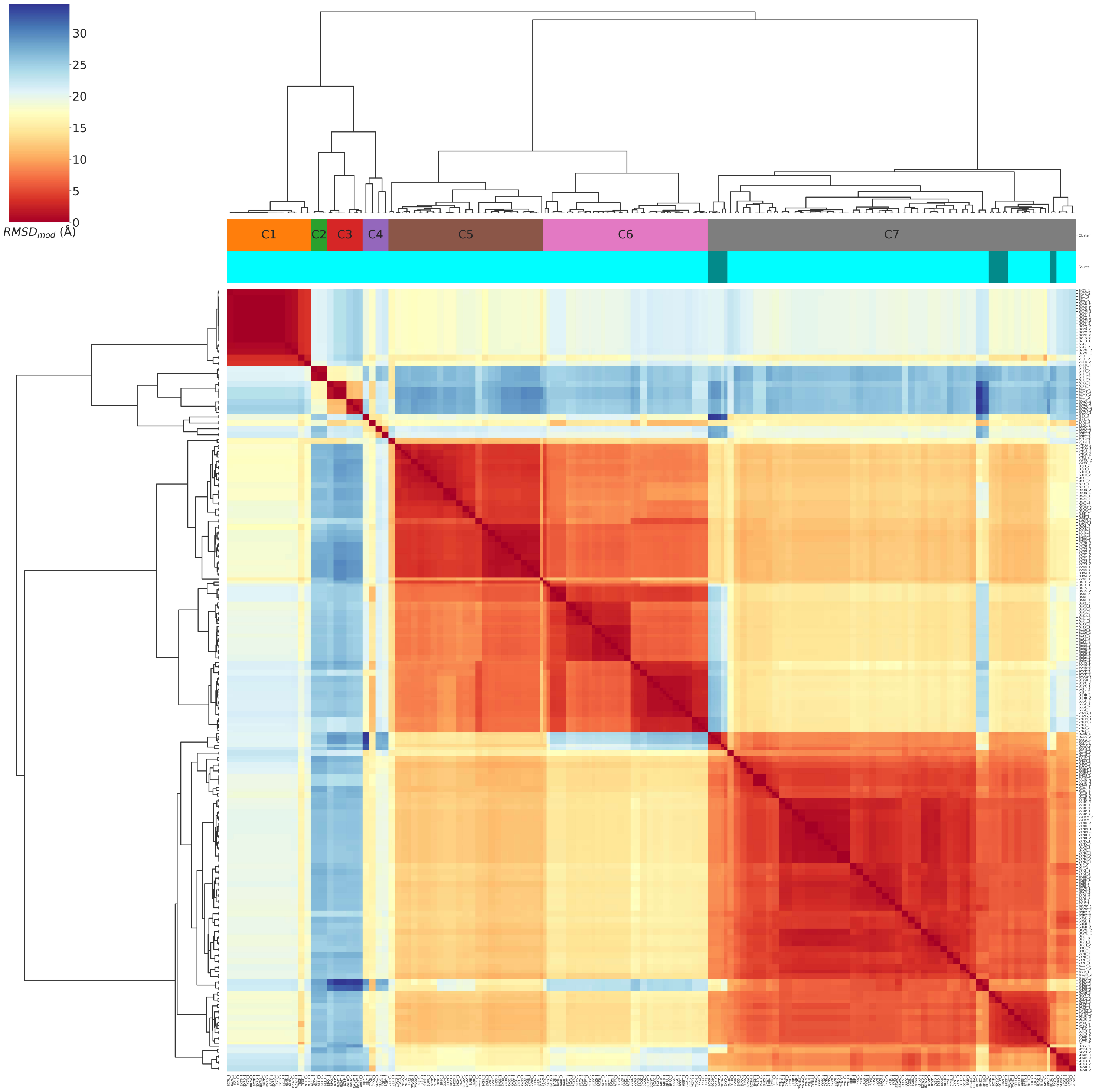

Supplementary Figure S5. Agglomerative clustering result of **α-syn** protofilaments, shown as a heat map of $RMSD_{mod}$ values. The dendrogram represents the relative variance in the clustering. Clusters (assigned with a limiting value of 30% ot the total $RMSD_{mod}$) are shown as the first colored row(color-coded as *C1*: orange, *C2*: green, *C3*: red, *C4*: purple, *C5*: brown, *C6*: pink, *C7*: grey) . The second colored row denotes the origin of the structure (cyan for in vitro, and dark cyan for ex vivo)

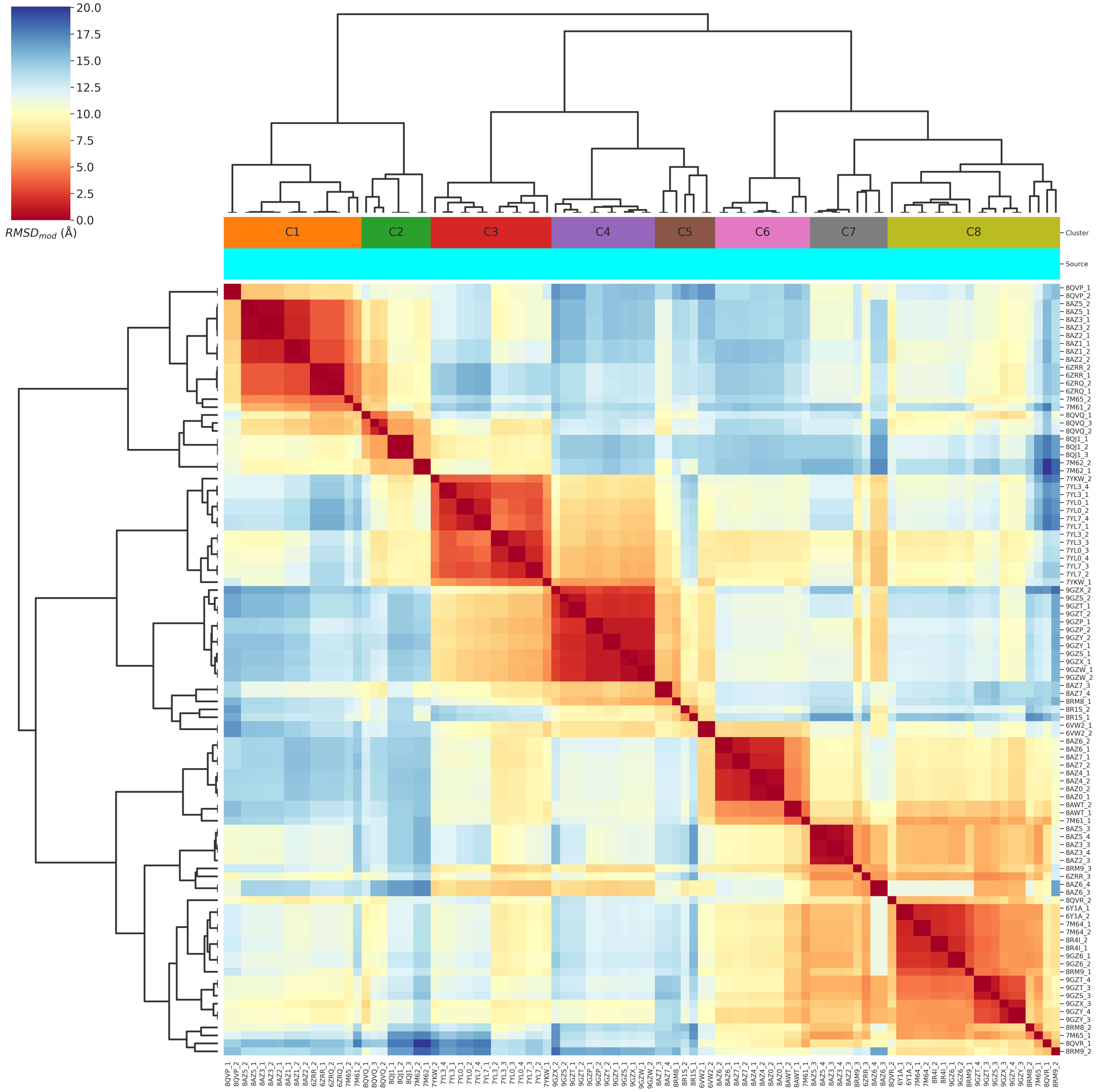

Supplementary Figure S6. Agglomerative clustering result of **IAPP** protofilaments, shown as a heat map of $RMSD_{mod}$ values. The dendrogram represents the relative variance in the clustering. Clusters (assigned with a limiting value of 30% ot the total $RMSD_{mod}$) are shown as the first colored row(color-coded as *C1*: orange, *C2*: green, *C3*: red, *C4*: purple, *C5*: brown, *C6*: pink, *C7*: grey, *C8*: olive) . The second colored row denotes the origin of the structure (cyan for in vitro, and dark cyan for ex vivo)

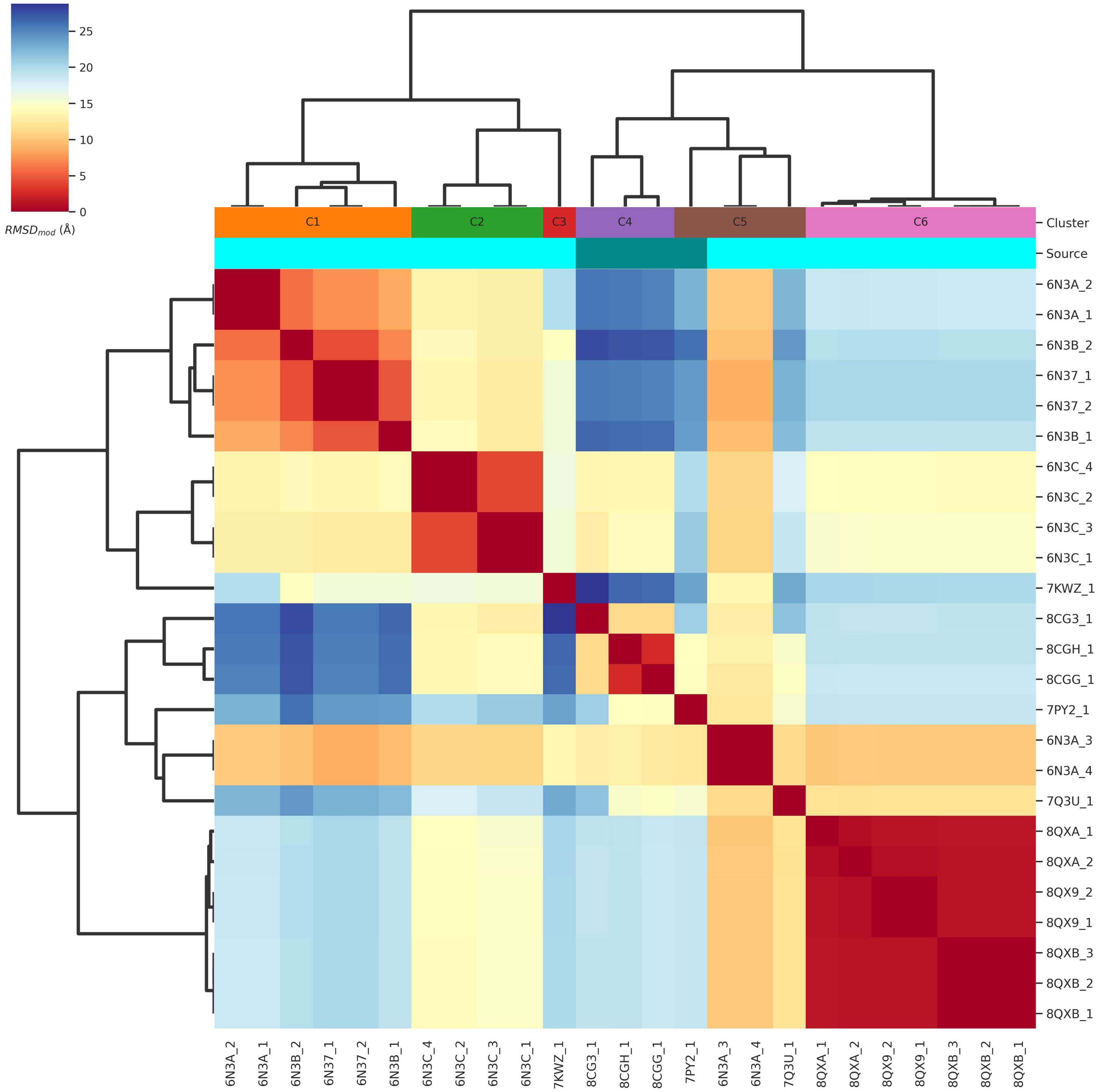

Supplementary Figure S7. Agglomerative clustering result of **TDP-43** protofilaments, shown as a heat map of $RMSD_{mod}$ values. The dendrogram represents the relative variance in the clustering. Clusters (assigned with a limiting value of 30% ot the total $RMSD_{mod}$) are shown as the first colored row(color-coded as *C1*: orange, *C2*: green, *C3*: red, *C4*: purple, *C5*: brown, *C6*: pink, *C7*: grey, *C8*: olive) . The second colored row denotes the origin of the structure (cyan for in vitro, and dark cyan for ex vivo)

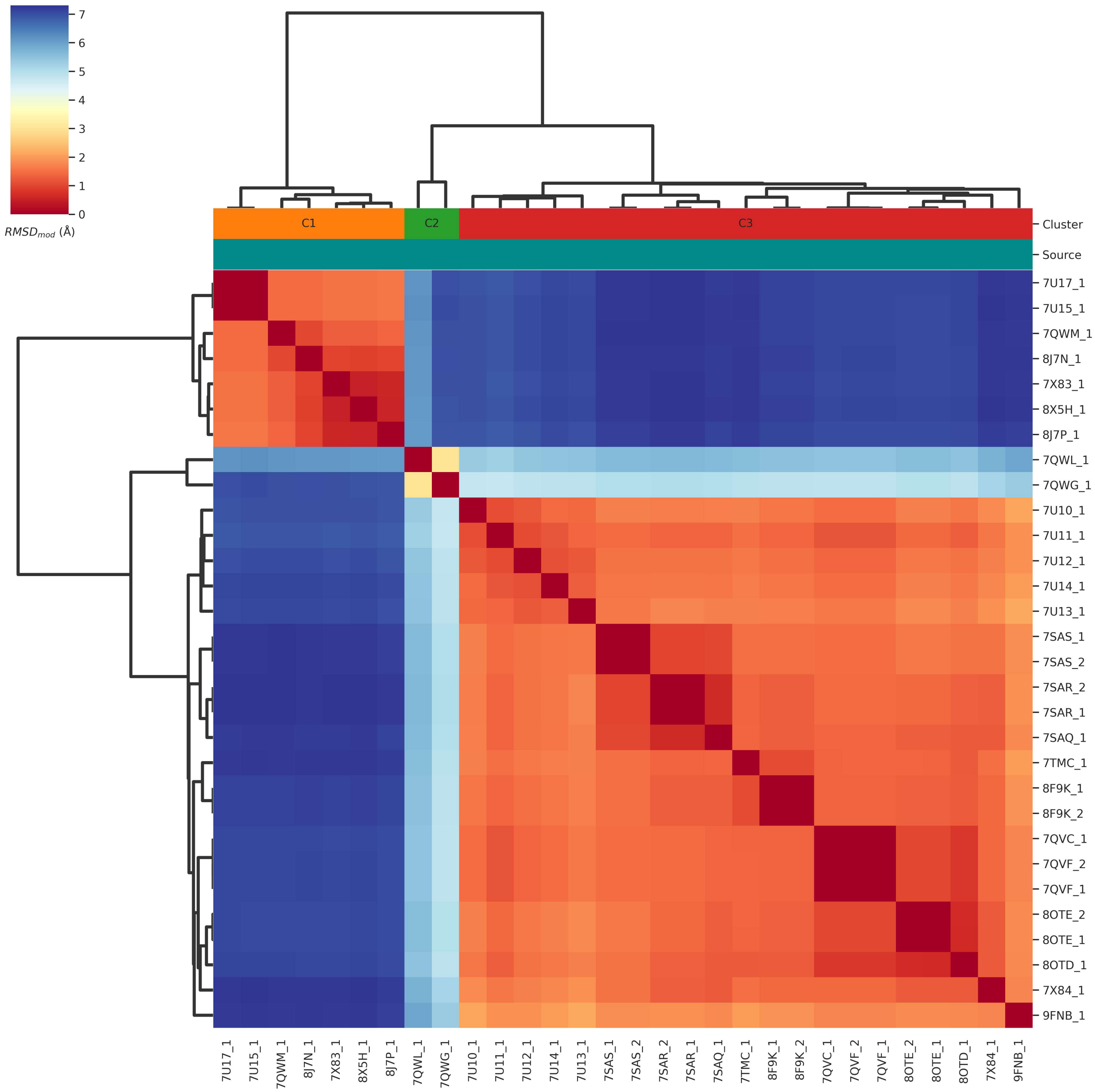

Supplementary Figure S8. Agglomerative clustering result of **TMEM106B** protofilaments, shown as a heat map of $RMSD_{mod}$ values. The dendrogram represents the relative variance in the clustering. Clusters (assigned with a limiting value of 30% ot the total $RMSD_{mod}$) are shown as the first colored row(color-coded as *C1*: orange, *C2*: green, *C3*: red) . The second colored row denotes the origin of the structure (cyan for in vitro, and dark cyan for ex vivo)

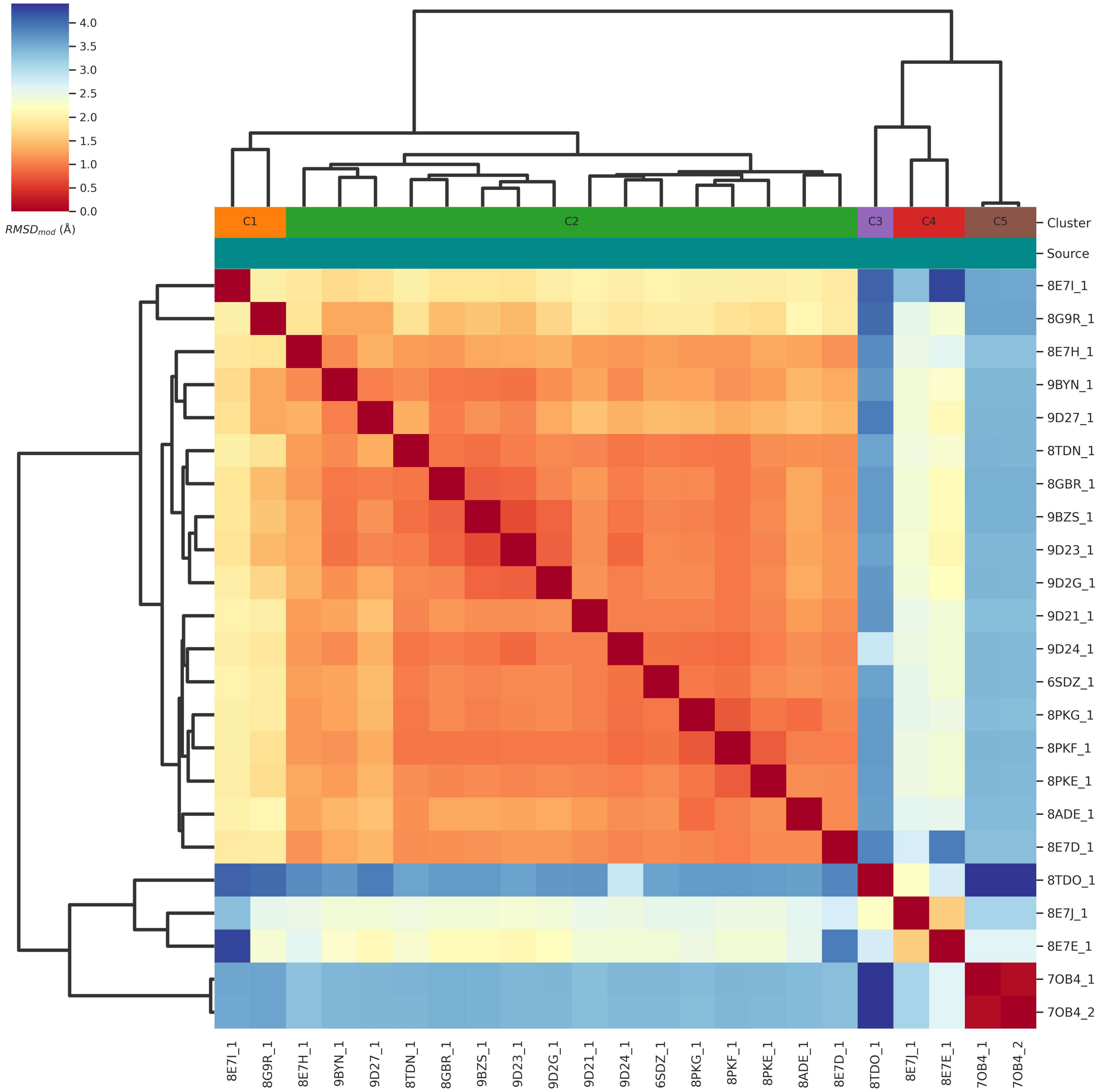

Supplementary Figure S9. Agglomerative clustering result of **TTR** protofilaments, shown as a heat map of $RMSD_{mod}$ values. The dendrogram represents the relative variance in the clustering. Clusters (assigned with a limiting value of 30% ot the total $RMSD_{mod}$) are shown as the first colored row(color-coded as *C1*: orange, *C2*: green, *C3*: purple, *C4*: red, *C5*: brown) . The second colored row denotes the origin of the structure (cyan for in vitro, and dark cyan for ex vivo)

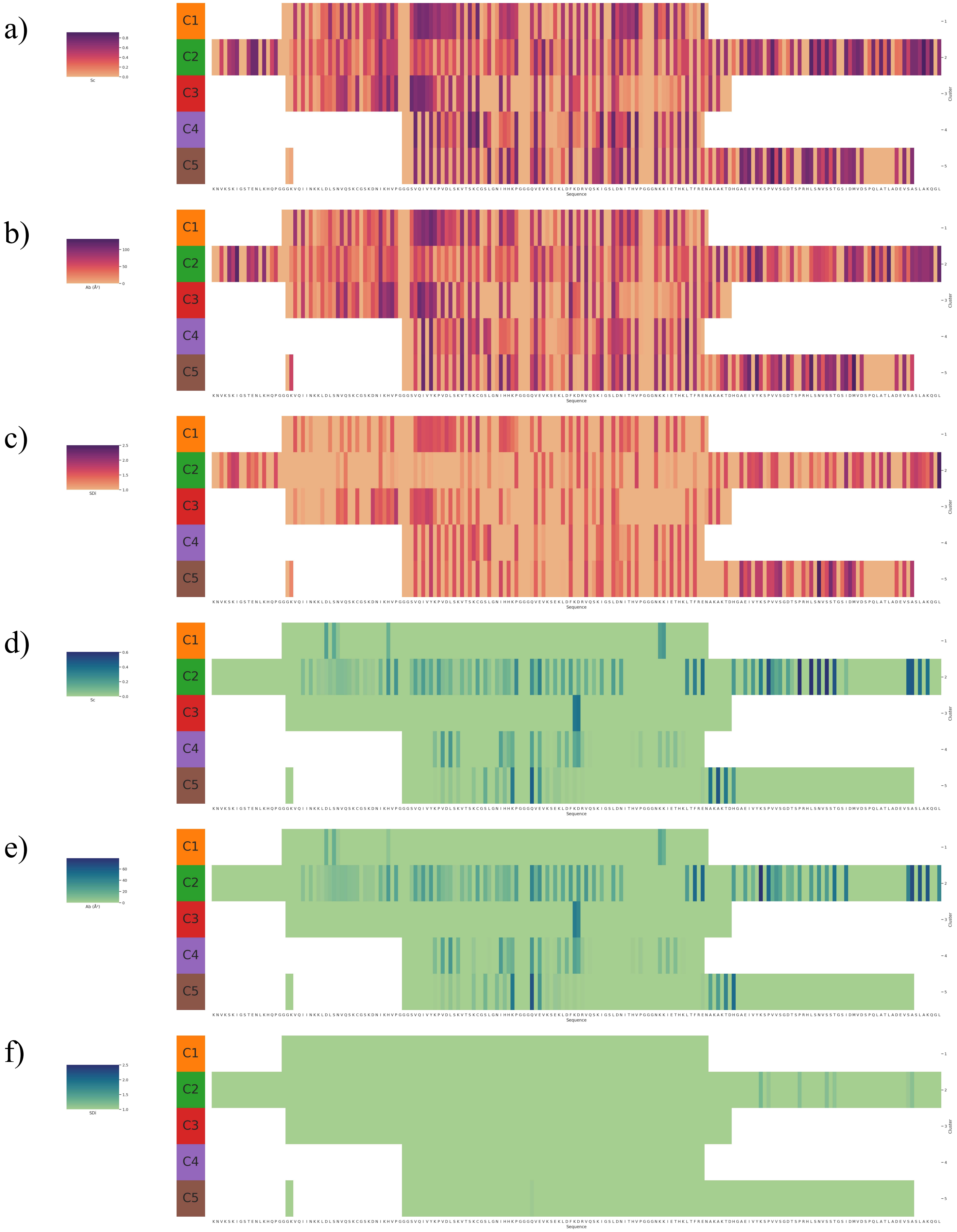

Supplementary Figure S10. Descriptor values of local interfaces of residues of **Tau** protofilaments. Local descriptor values averaged within each clusters, are plotted against the amino acid sequence as heatmaps. **a,d**) plots of Sc; **b,e**) plots of Ab; **c,f)** plots of SDi
a,b,c) all interfaces considered, colored as shades of orange; **d,e,f**) only inter-protofilament interfaces considered, colored as shades of green.

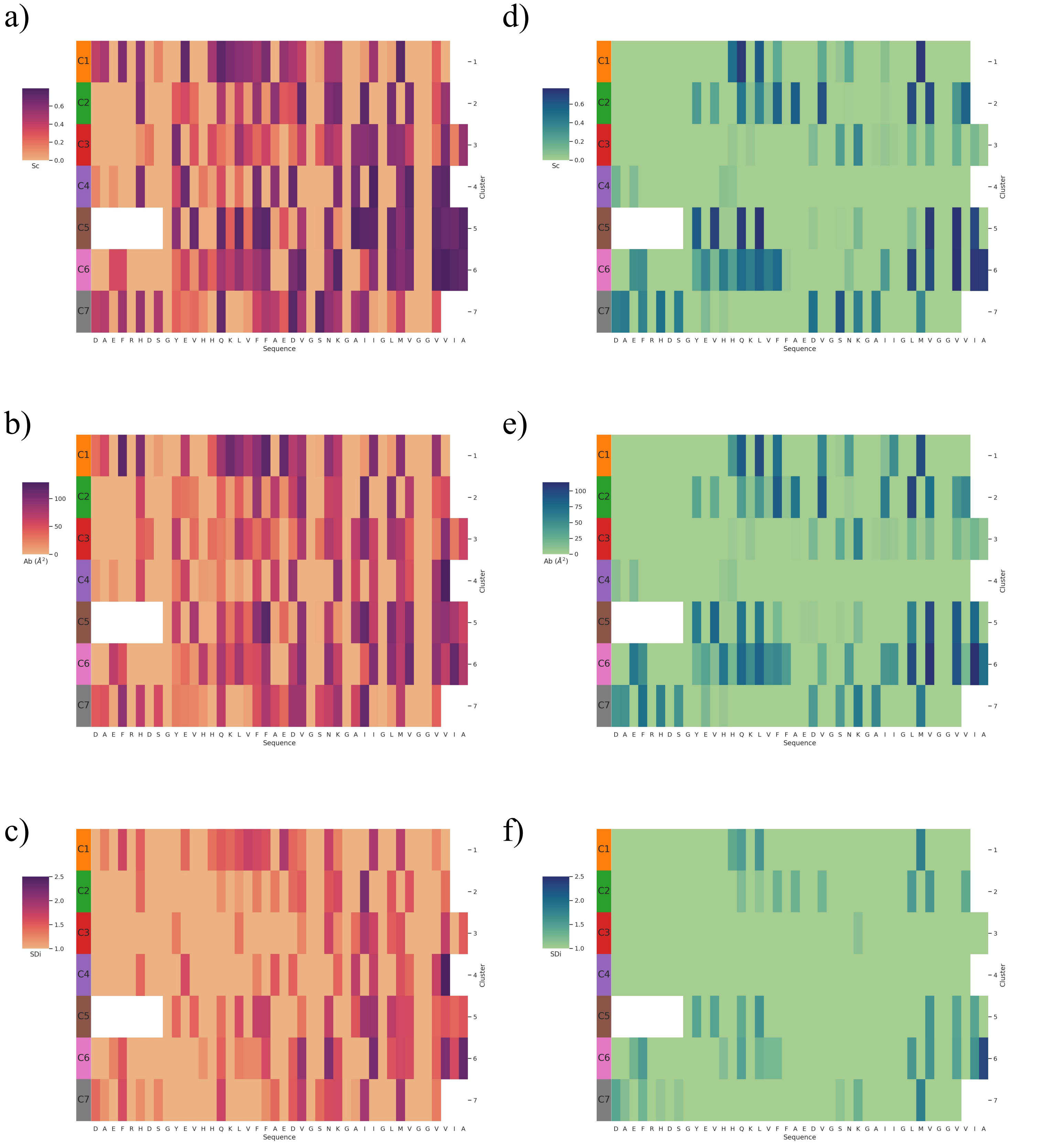

Supplementary Figure S11. Descriptor values of local interfaces of residues of **Aβ** protofilaments. Local descriptor values averaged within each clusters, are plotted against the amino acid sequence as heatmaps. **a,d**) plots of Sc; **b,e**) plots of Ab; **c,f)** plots of SDi
a,b,c) all interfaces considered, colored as shades of orange; **d,e,f**) only inter-protofilament interfaces considered, colored as shades of green.

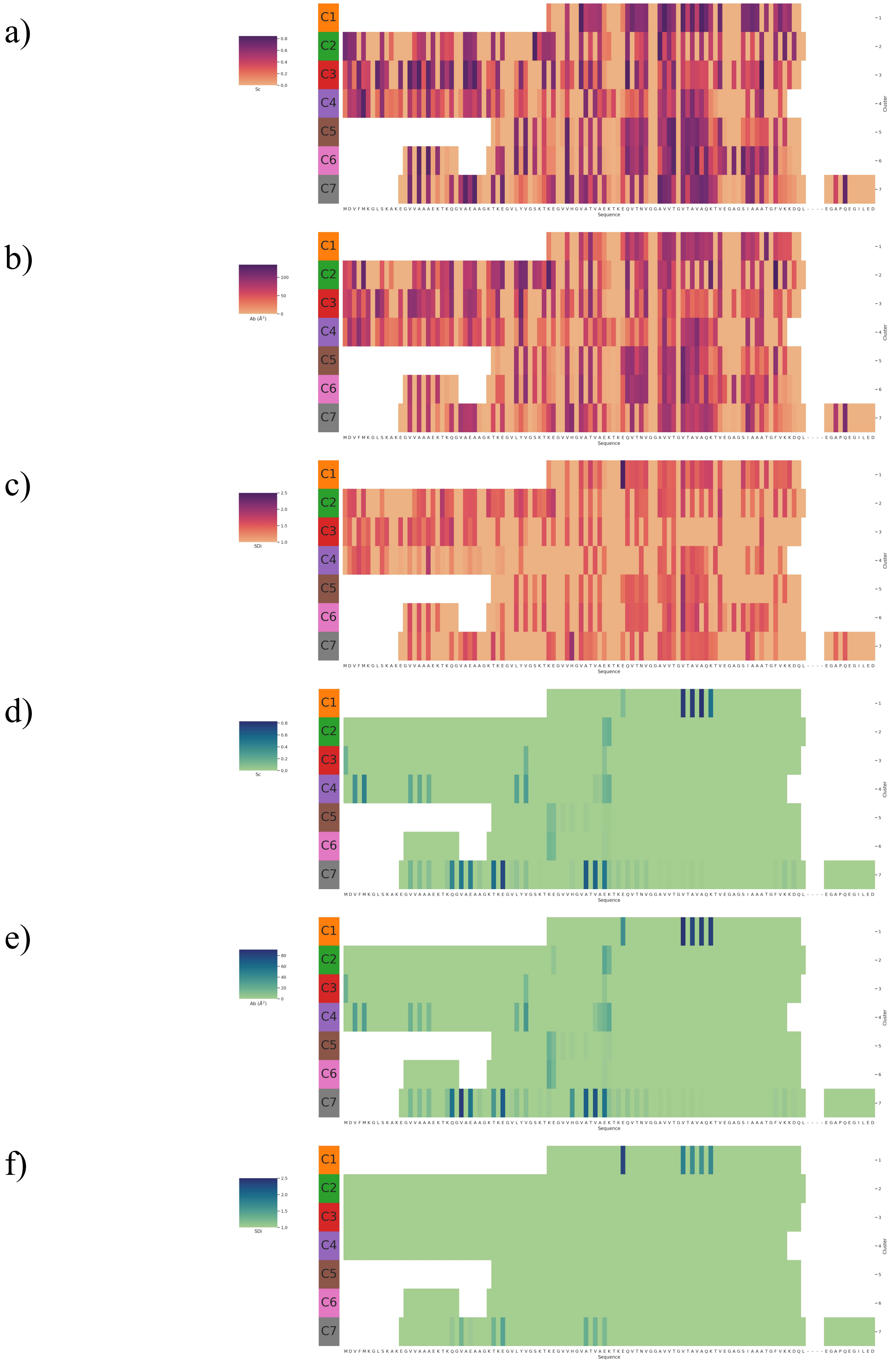

Supplementary Figure S12. Descriptor values of local interfaces of residues of **α-syn** protofilaments. Local descriptor values averaged within each clusters, are plotted against the amino acid sequence as heatmaps. **a,d**) plots of Sc; **b,e**) plots of Ab; **c,f)** plots of SDi
a,b,c) all interfaces considered, colored as shades of orange; **d,e,f**) only inter-protofilament interfaces considered, colored as shades of green.

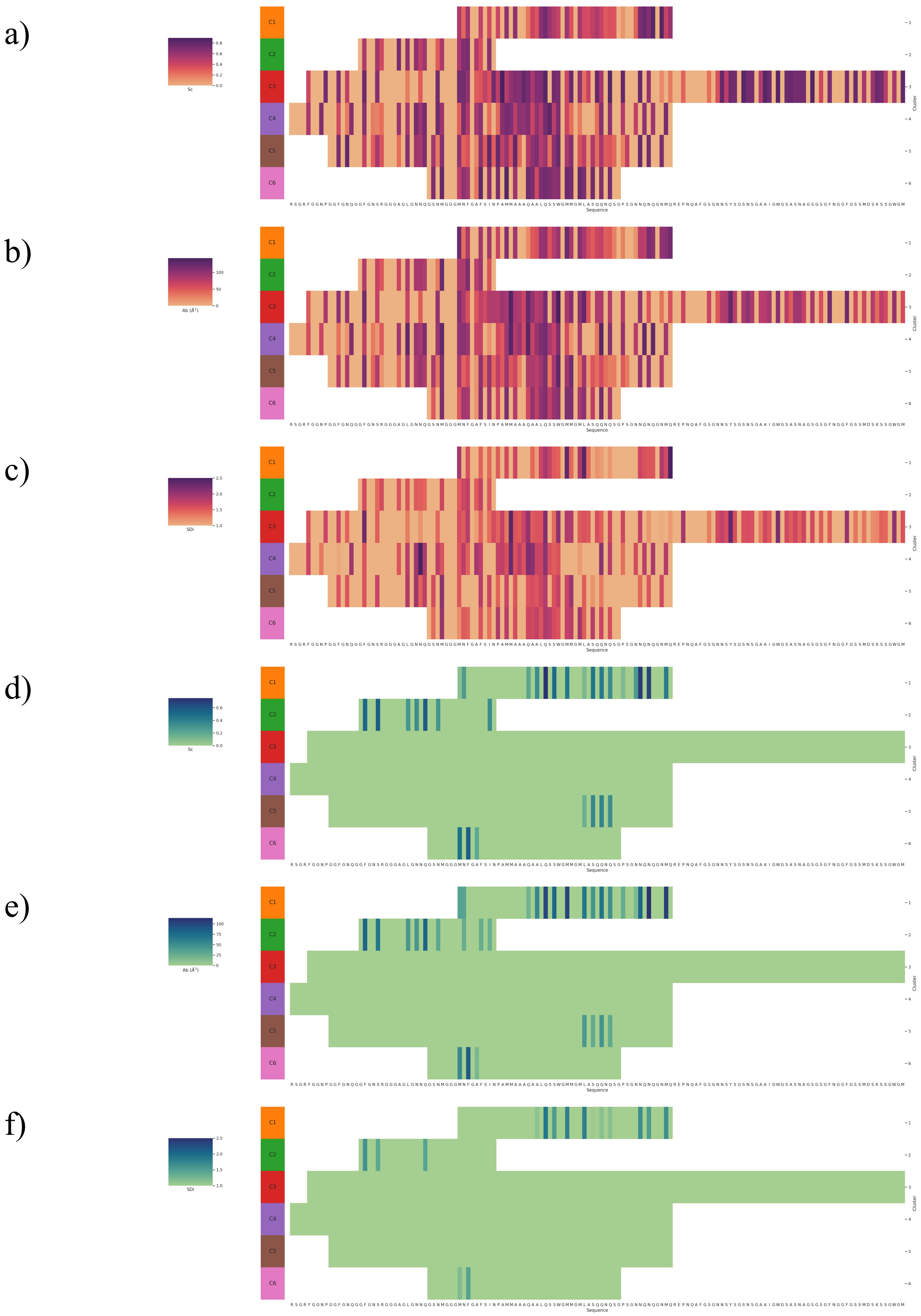

Supplementary Figure S13. Descriptor values of local interfaces of residues of **TDP-43** protofilaments. Local descriptor values averaged within each clusters, are plotted against the amino acid sequence as heatmaps. **a,d**) plots of Sc; **b,e**) plots of Ab; **c,f)** plots of SDi
a,b,c) all interfaces considered, colored as shades of orange; **d,e,f**) only inter-protofilament interfaces considered, colored as shades of green.

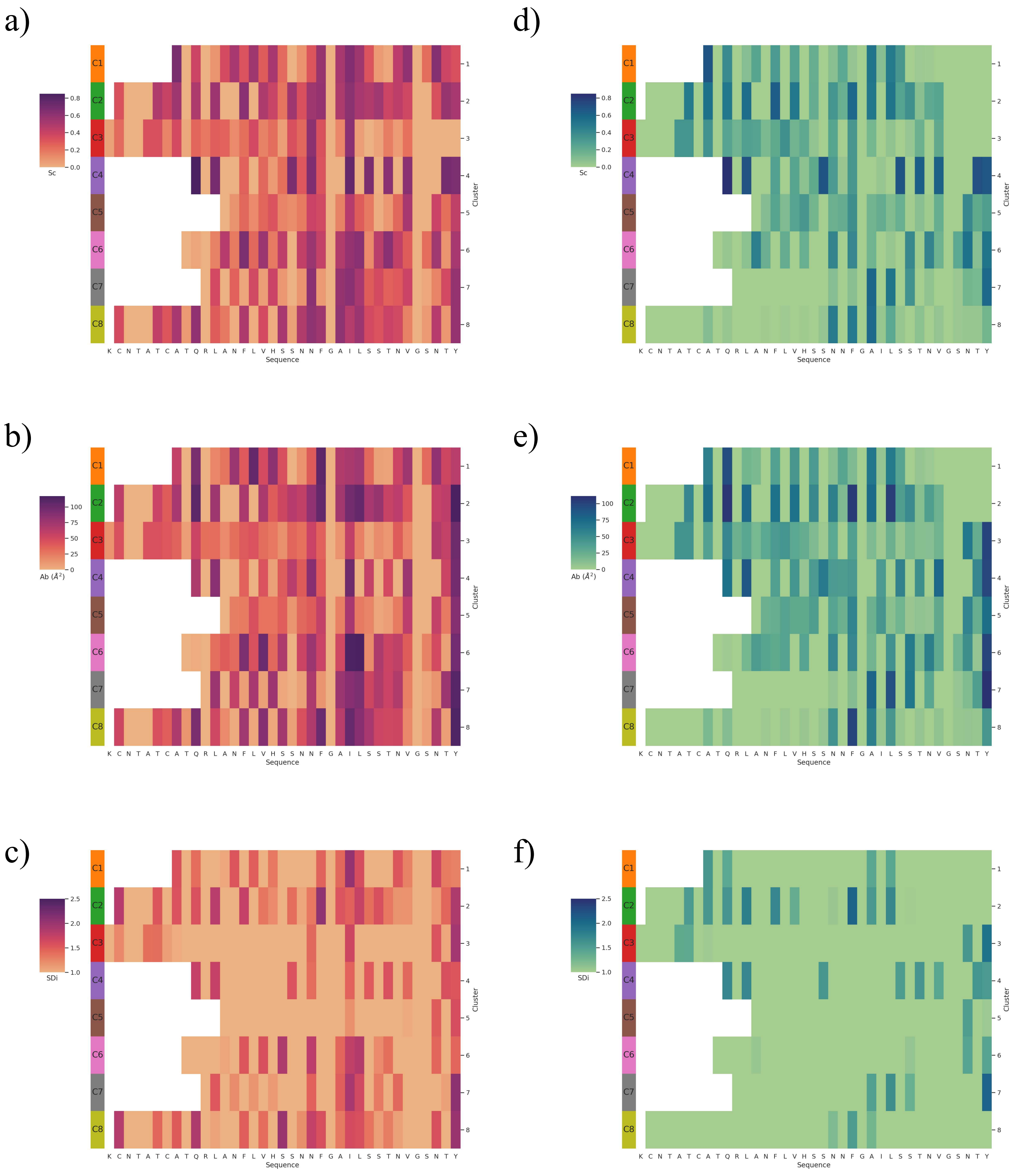

Supplementary Figure S14. Descriptor values of local interfaces of residues of **IAPP** protofilaments. Local descriptor values averaged within each clusters, are plotted against the amino acid sequence as heatmaps. **a,d**) plots of Sc; **b,e**) plots of Ab; **c,f)** plots of SDi
a,b,c) all interfaces considered, colored as shades of orange; **d,e,f**) only inter-protofilament interfaces considered, colored as shades of green.

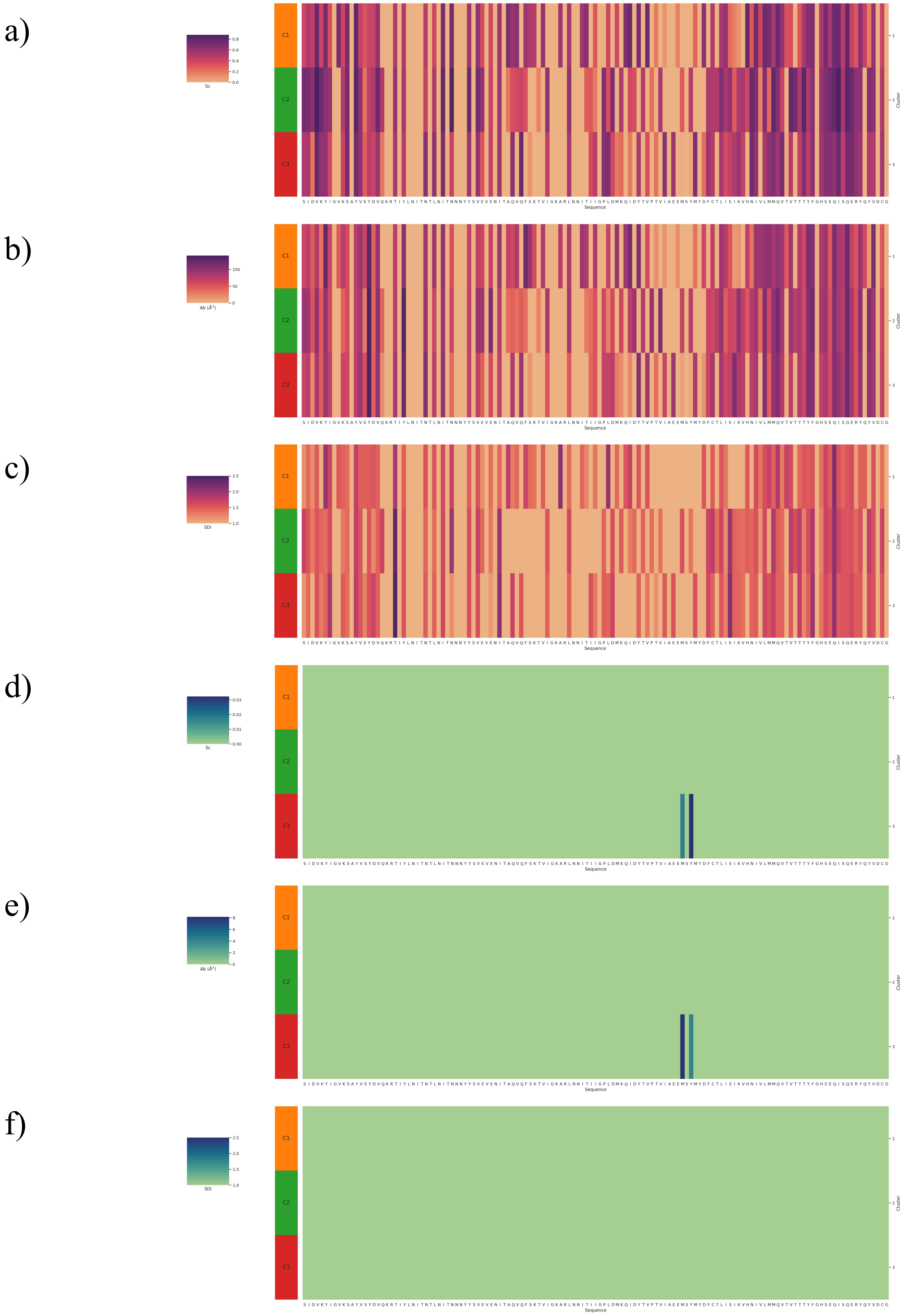

Supplementary Figure S15. Descriptor values of local interfaces of residues of **TMEM106B** protofilaments. Local descriptor values averaged within each clusters, are plotted against the amino acid sequence as heatmaps. **a,d**) plots of Sc; **b,e**) plots of Ab; **c,f)** plots of SDi
a,b,c) all interfaces considered, colored as shades of orange; **d,e,f**) only inter-protofilament interfaces considered, colored as shades of green.

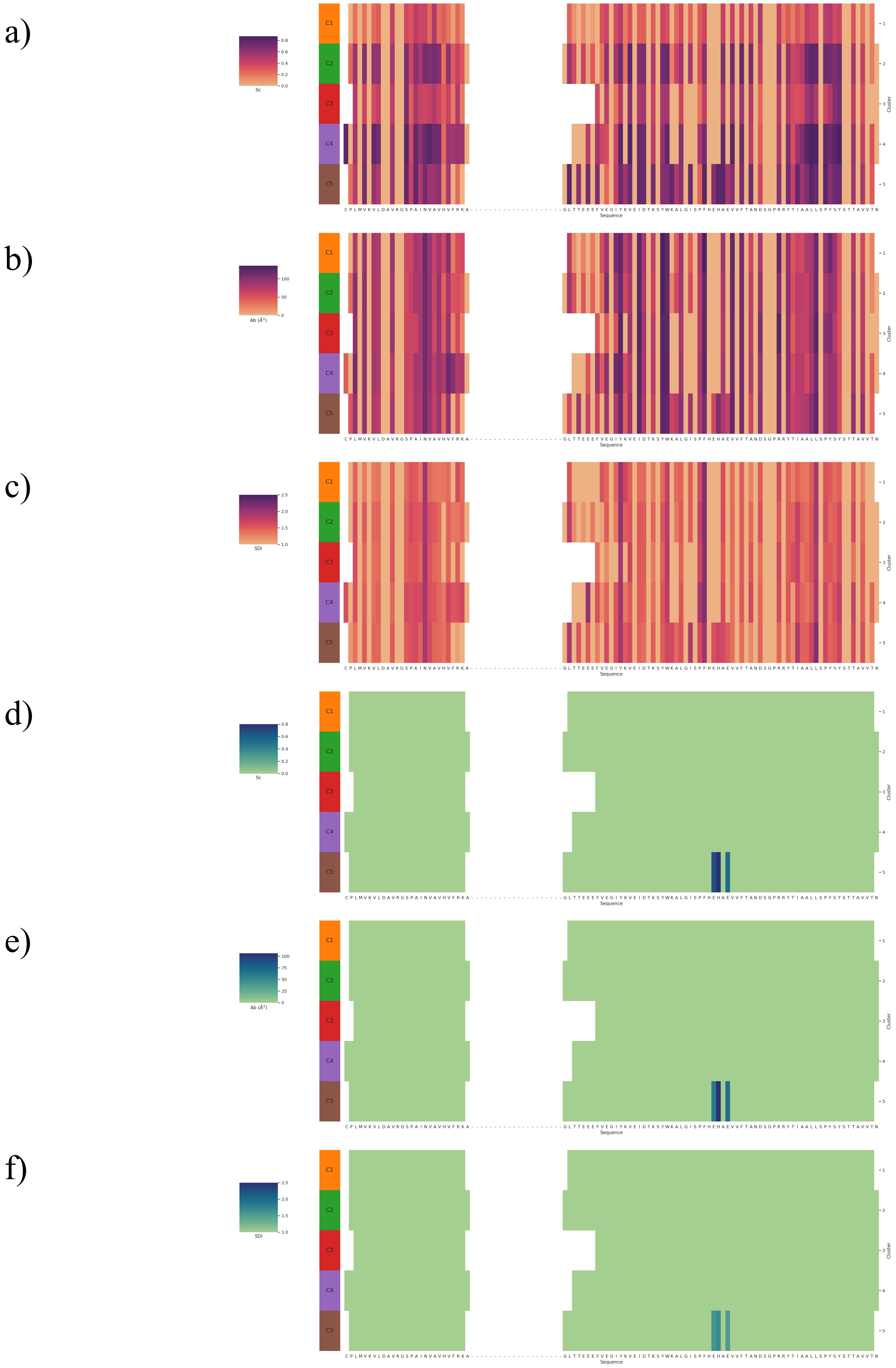

Supplementary Figure S16. Descriptor values of local interfaces of residues of **TTR** protofilaments. Local descriptor values averaged within each clusters, are plotted against the amino acid sequence as heatmaps. **a,d**) plots of Sc; **b,e**) plots of Ab; **c,f)** plots of SDi

a,b,c) all interfaces considered, colored as shades of orange; **d,e,f**) only inter-protofilament interfaces considered, colored as shades of green.

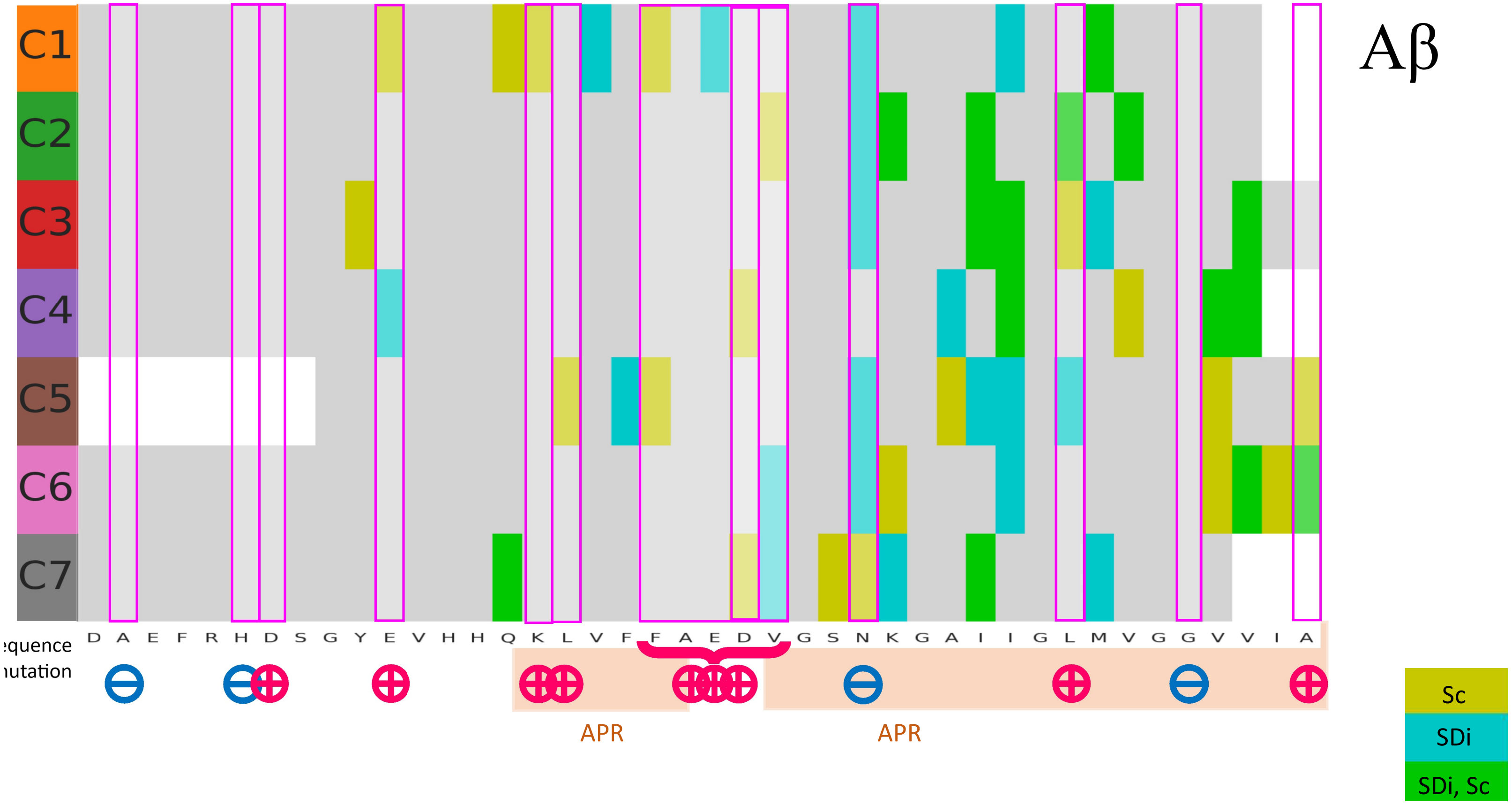

Supplementary Figure S17. Relative positions of hot-spot residues and mutations effecting aggregation propensity for Aβ. Hot spot residues are colored: yellow: top 5 Sc values within the cluster; cyan: top 5 SDi values within the cluster; green: both. Clusters are shown as rows (color-coded in the first column as *C1*: orange, *C2*: green, *C3*: red, *C4*: purple, *C5*: brown, *C6*: pink, *C7*: grey). Sites for mutations effecting the aggregation or formation of filaments according to the study of Seuma et al.[S1] are framed with magenta and labelled in the last row with + (promoting aggregation) or – (inhibiting aggregation). APR labelled segments are shown below the sequence with light brown: these are overlapping oligopeptide segments with their amyloid crystal structures solved.

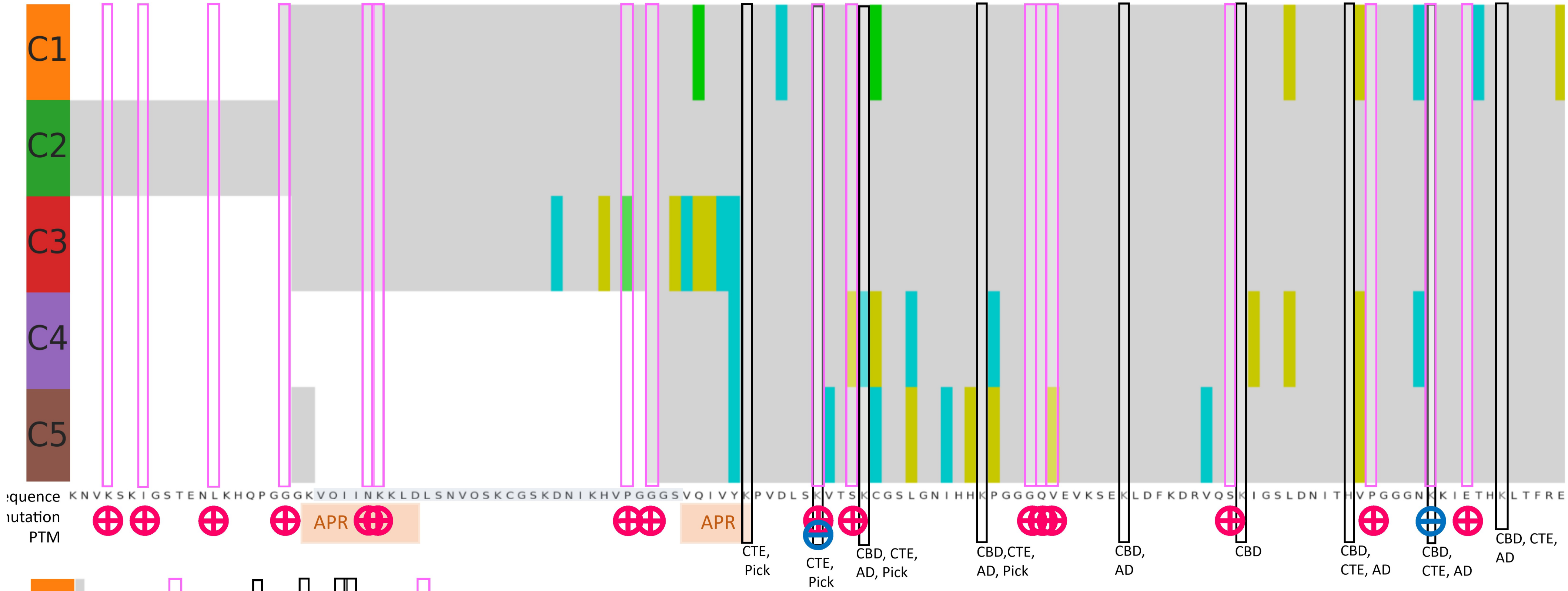

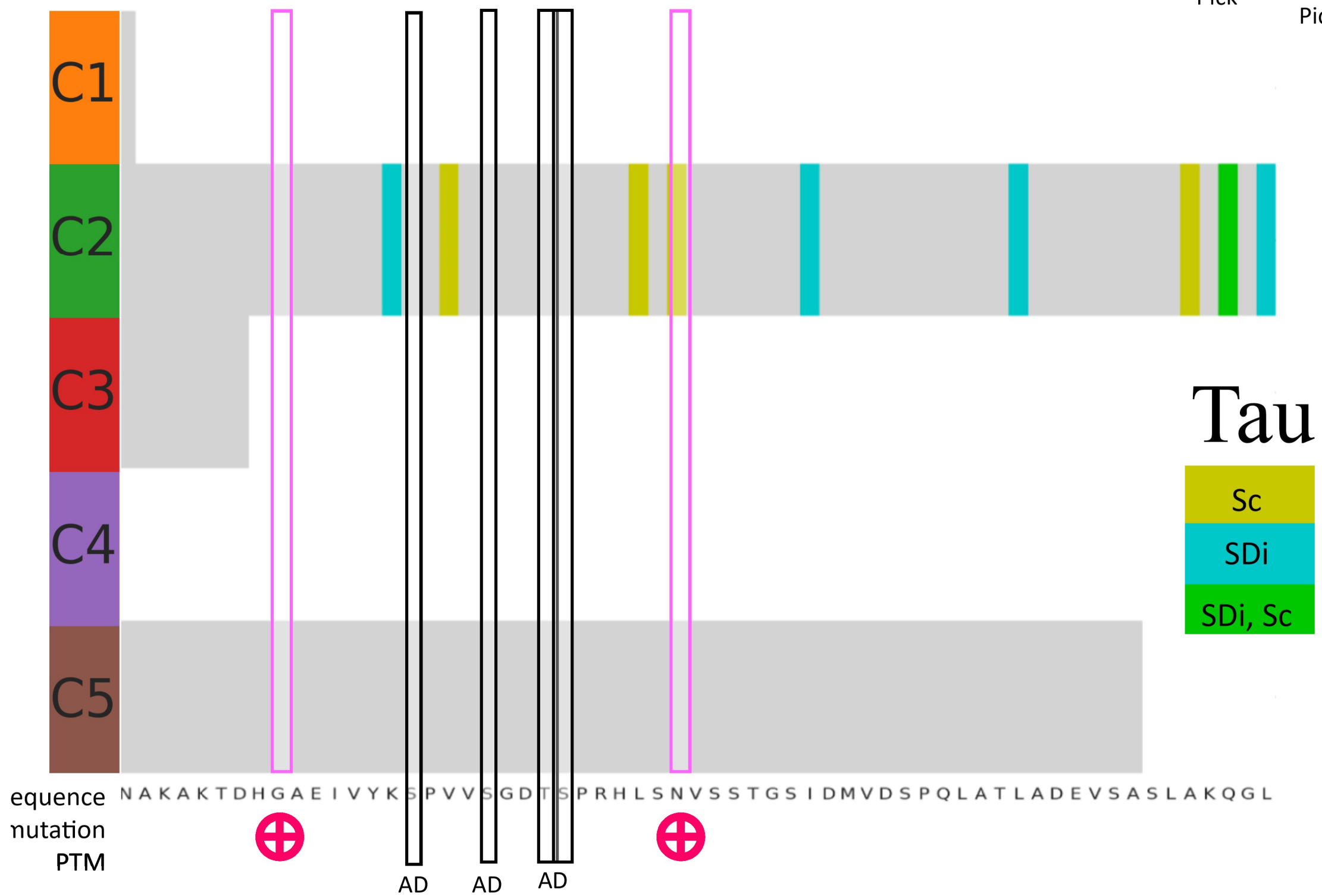

Supplementary Figure S18. Relative positions of hot-spot residues and mutations effecting aggregation propensity for Tau. Hot spot residues are colored: yellow: top 5 Sc values within the cluster; cyan: top 5 SDi values within the cluster; green: both. Clusters are shown as rows (color-coded in the first column as *C1*: orange, *C2*: green, *C3*: red, *C4*: purple, *C5*: brown). Sites for mutations effecting the aggregation or formation of filaments according to the review of Strang et al.[S2] and selected post-translational modifications (phosphorylation) according to Li et al.[S3] are framed with magenta and black, respectively and labelled in the last row with + (promoting aggregation) or – (inhibiting aggregation). APR labelled segments are shown below the sequence with light brown: these are overlapping oligopeptide segments with their amyloid crystal structures solved.

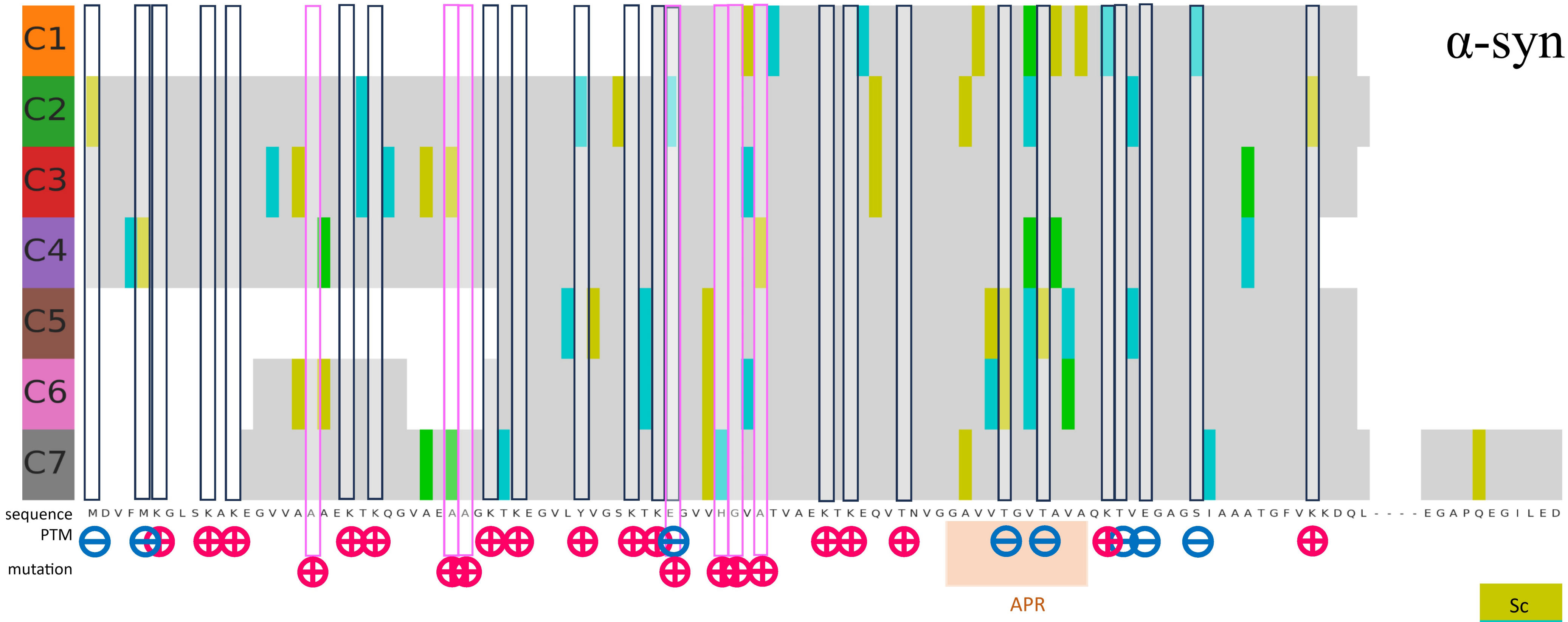

Supplementary Figure S19. Relative positions of hot-spot residues and mutations and post-translational modifications effecting aggregation propensity for α-syn. Hot spot residues are colored: yellow: top 5 Sc values within the cluster; cyan: top 5 SDi values within the cluster; green: both. Clusters are shown as rows (color-coded in the first column as *C1*: orange, *C2*: green, *C3*: red, *C4*: purple, *C5*: brown, *C6*: pink, *C7*: grey). Sites for mutations effecting the aggregation or formation of filaments according to the study of Lazaro et al.[S4] are framed with magenta and labelled in the last row with + (promoting aggregation) or – (inhibiting aggregation). Similarly, the post translational modifications (PTMs) are shown with black frames and + or – signs, as reviewed by Hassanzadeh et al.[S5] (Note, sites with missing residue Ids or uncertain promoting/inhibiting effects are not shown.) APR labelled segments are shown below the sequence with light brown: these are overlapping oligopeptide segments with their amyloid crystal structures solved.

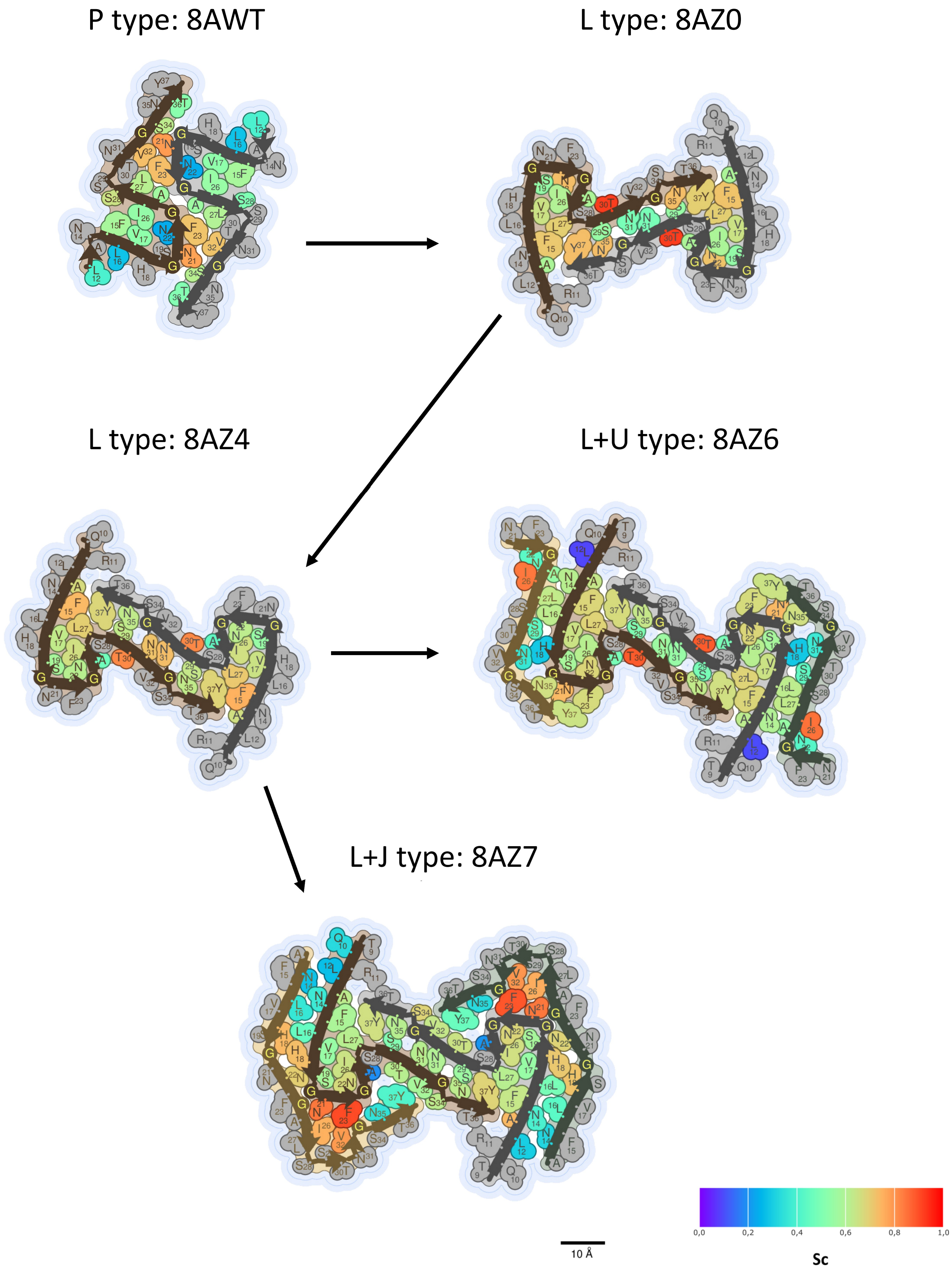

Supplementary Figure S20. Maturation pathway of P type and L-lineage IAPP structures[S6] (8AWT, 8AZ0, 8AZ4, 8AZ6, 8AZ7). Visualized using the Amyloid Atlas Illustrator, colored by Sc values (blue –red: 0–1; grey: no local interface)

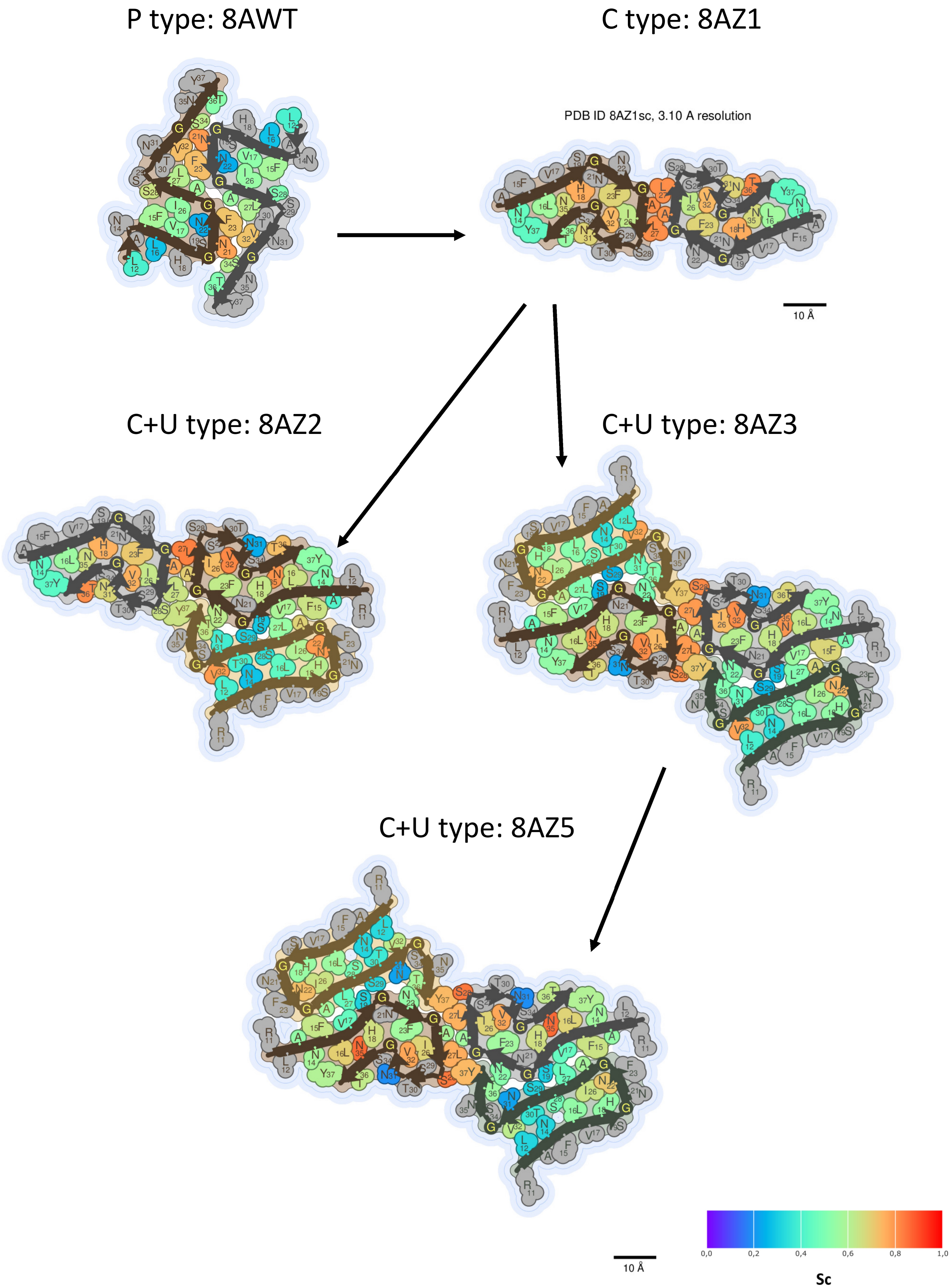

Supplementary Figure S21. Maturation pathway of P type and C-lineage IAPP structures[S6] (8AWT, 8AZ1, 8AZ2, 8AZ3, 8AZ5). Visualized using the Amyloid Atlas Illustrator, colored by Sc values (blue–red: 0–1; grey: no local interface)

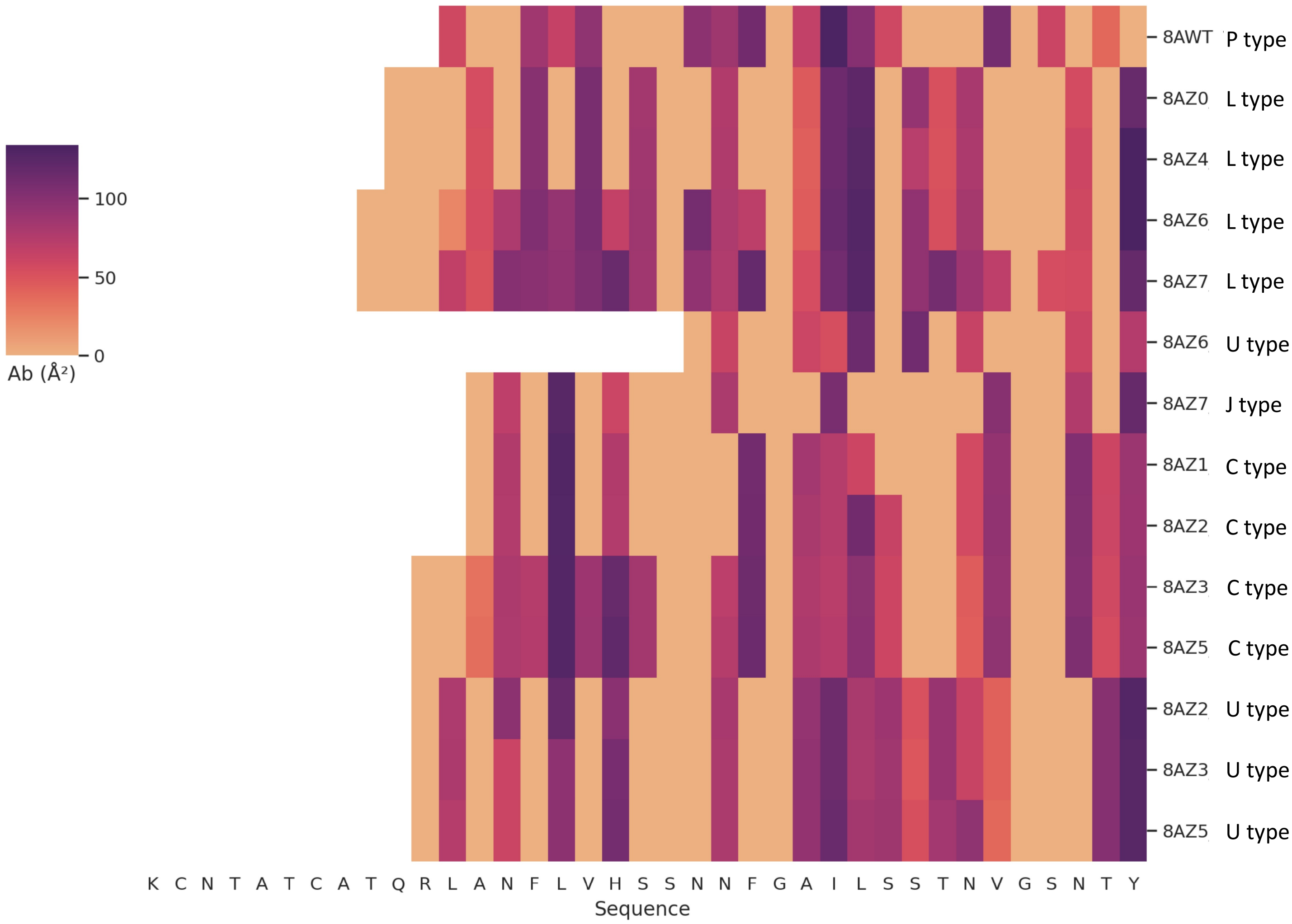

Supplementary Figure S22. Maturation of IAPP structures. Heatmap shown for the various filament types, colored according to the local interface Ab values.

a)

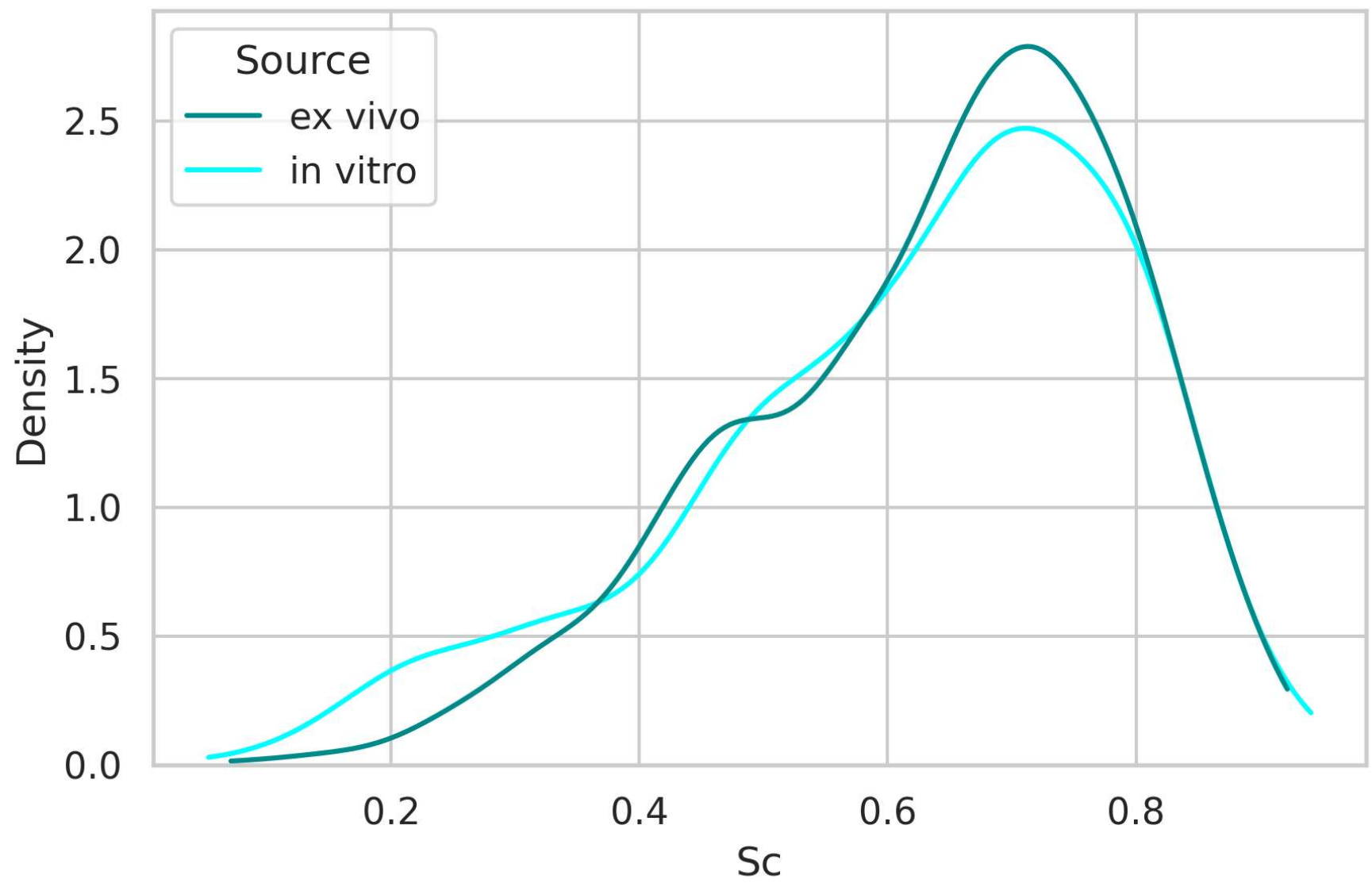

b)

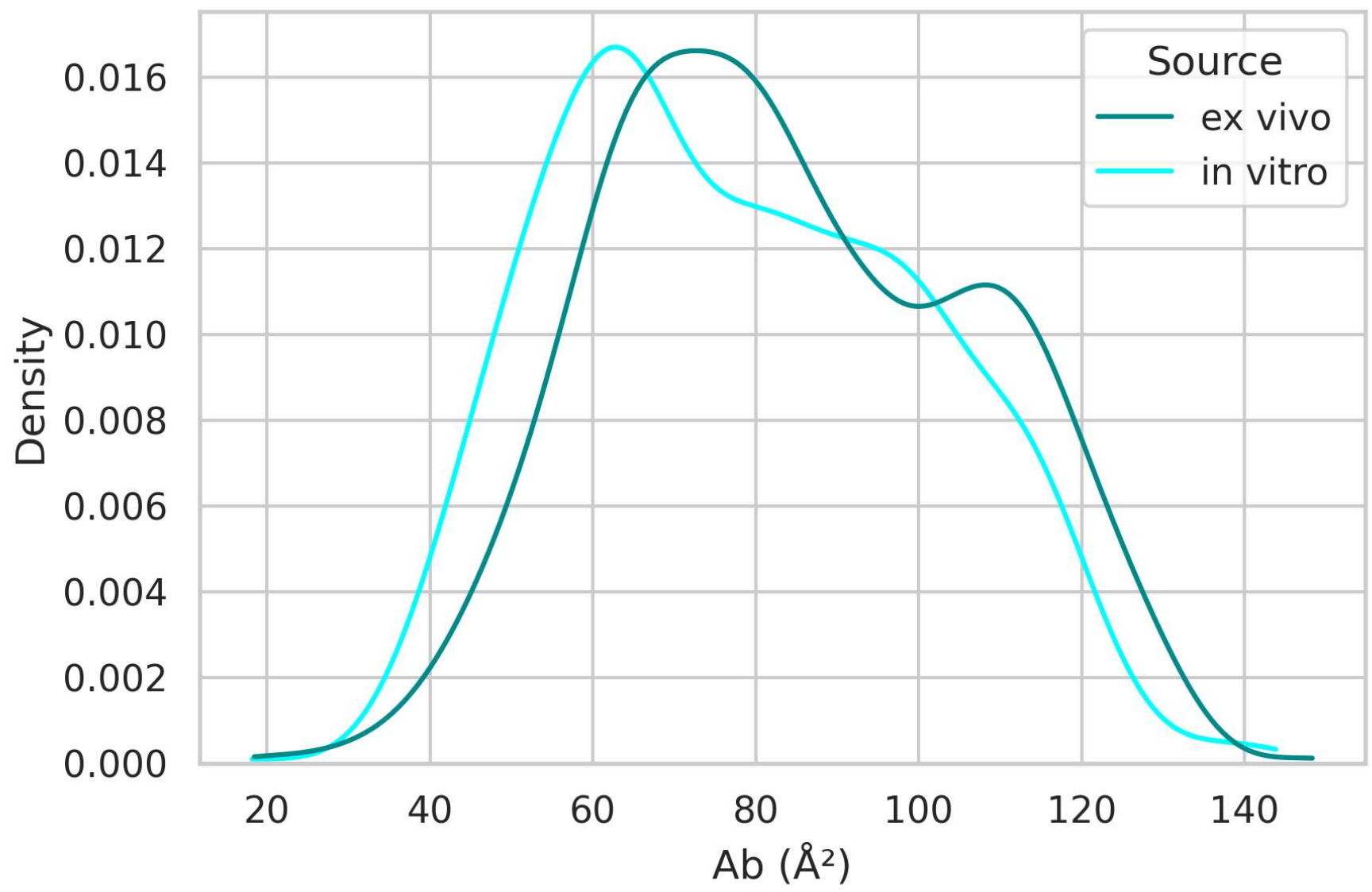

c)

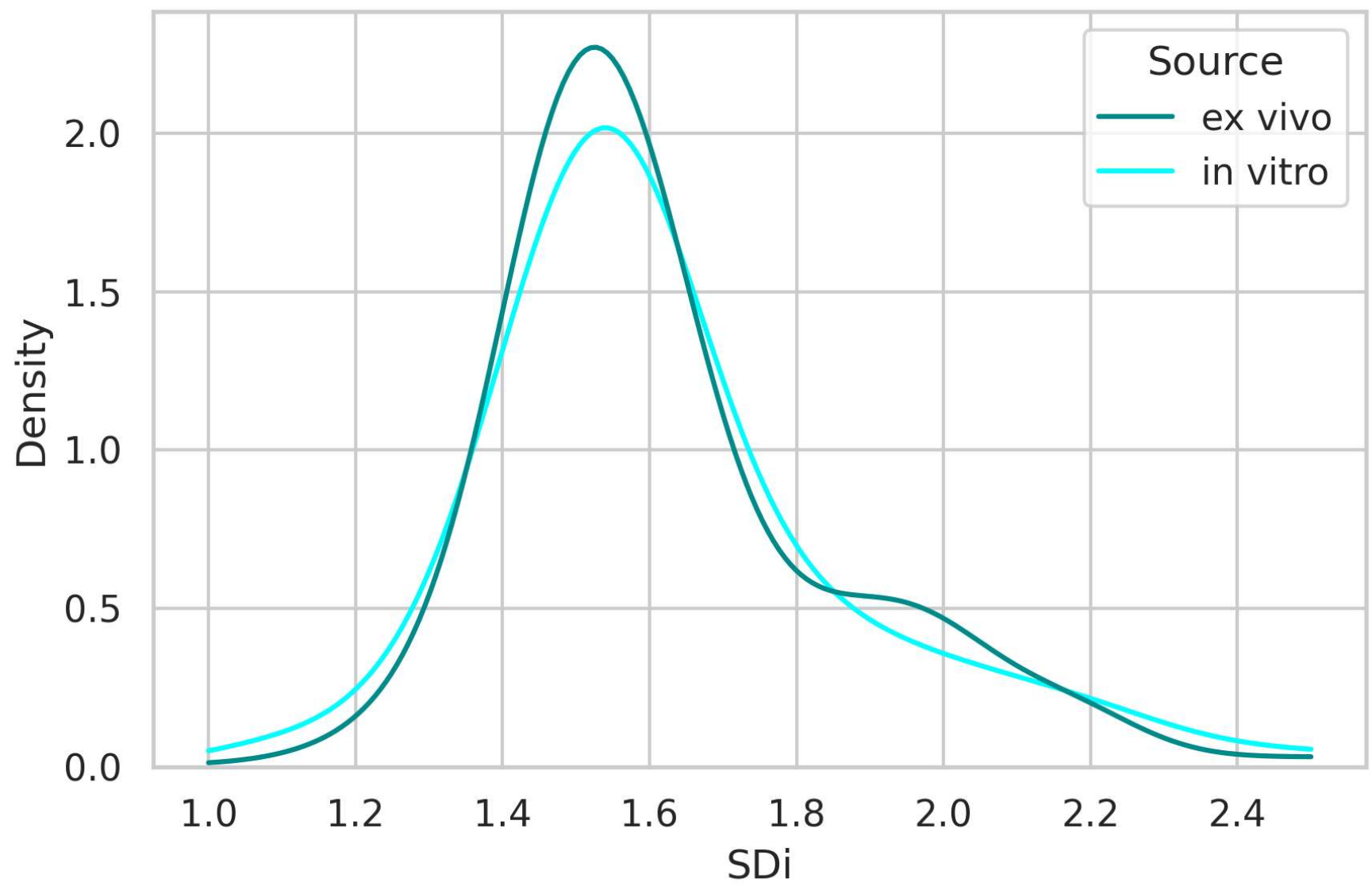

Supplementary Figure S23. Long time-scale effect of maturation on interface characteristics of Aβ Comparison of KDE distributions of local descriptor values of in vitro and ex vivo experiments of Aβ. **a)** Sc, **b)** Ab, **c)** SDi

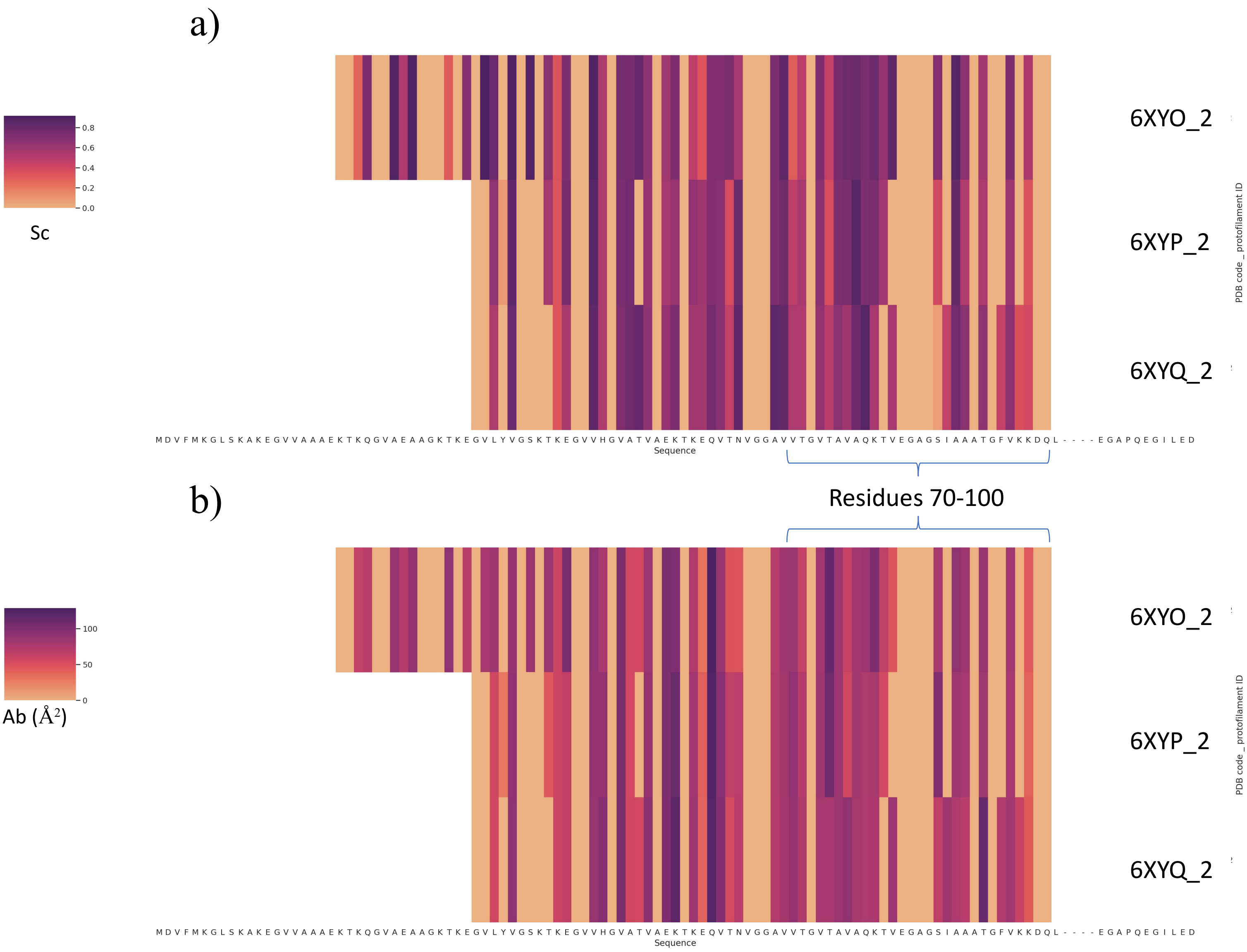

Supplementary Figure S24. Long time-scale effect of maturation on interface characteristics in α-syn. Heatmaps are shown for the second protofilament from early fold of PDB structure 6XYO and late structures of 6XYP and 6XYQ. **a)** local Sc values **b)** local Ab values.

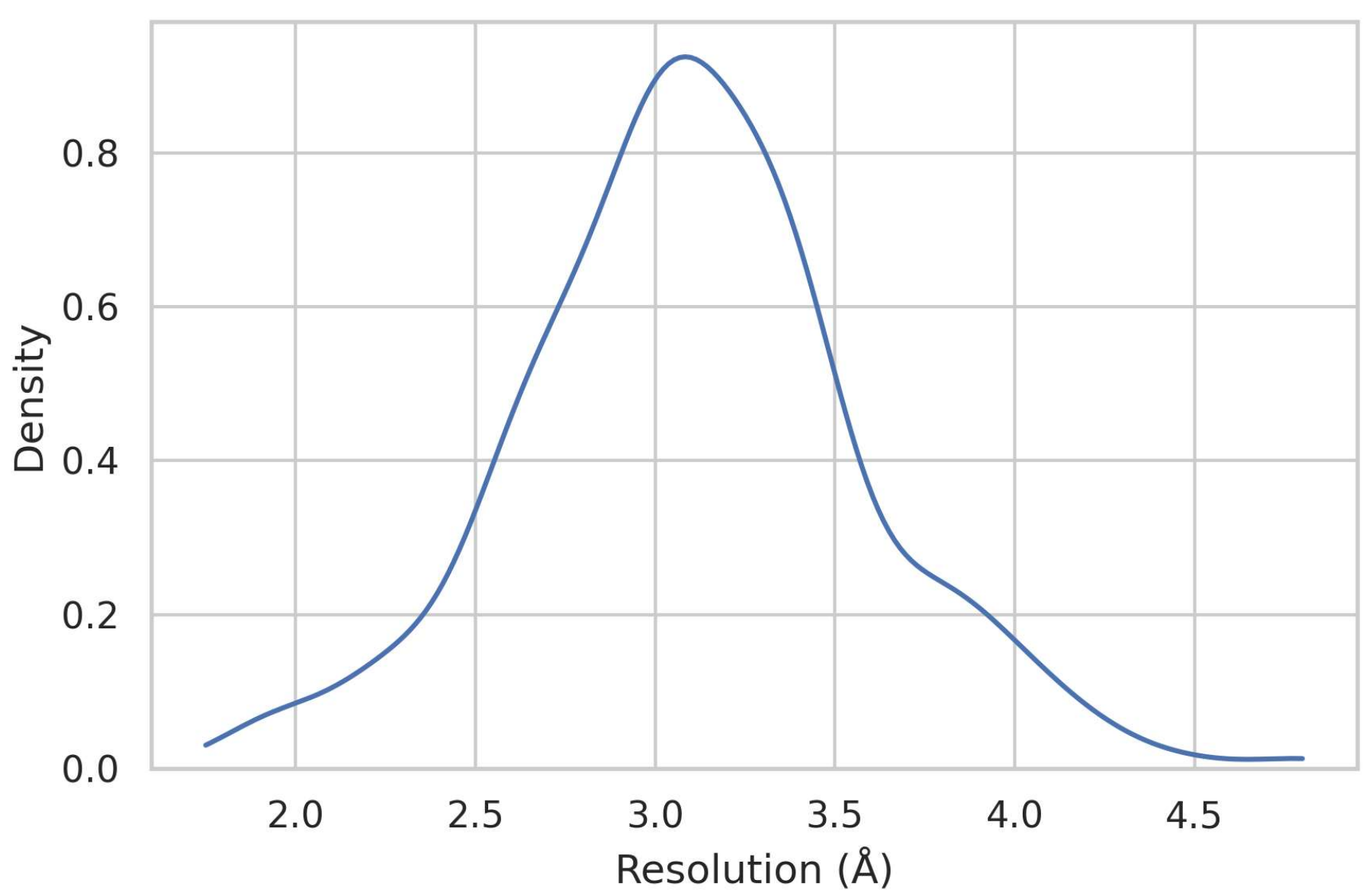

Supplementary Figure S25. KDE distribution of reported resolutions for all structures analyzed.

References

(S1) Seuma, M.; Lehner, B.; Bolognesi, B. An Atlas of Amyloid Aggregation: The Impact of Substitutions, Insertions, Deletions and Truncations on Amyloid Beta Fibril Nucleation. Nat Commun 2022, 13, 7084. https://doi.org/10.1038/s41467-022-34742-3

(S2) Strang, K.H.; Golde,T.E.; Giasson, B.I. MAPT mutations, tauopathy, and mechanisms of neurodegeneration. Laboratory Investigation 2019, 99, 912. https://doi.org/10.1038/s41374-019-0197-x

(S3) Taniguchi-Watanabe, S.; Arai,T.; Kametan, F.; Nonaka, T.; Masuda-Suzukake, M.; Tarutani, A.; Murayama, S.; Saito, Y.; Arima, K.; Yoshida, M.; Akiyama, H.; Robinson, A.; Mann,D.M.A.; Iwatsubo, T.; Hasegawa, M. Biochemical classification of tauopathies by immunoblot, protein sequence and mass spectrometric analyses of sarkosyl-insoluble and trypsin-resistant tau. Acta Neuropathol. 2016, 31, 267. https://doi.org/10.1007/s00401-015-1503-3

(S4) Lázaro, D.F.; Rodrigues, E.F.; Langohr, R.; Shahpasandzadeh, H.; Ribeiro, T.; et al. Systematic Comparison of the Effects of Alpha-synuclein Mutations on Its Oligomerization and Aggregation. PLoS Genet 2014, 10, e1004741. https://doi.org/10.1371/journal.pgen.1004741

(S5) Hassanzadeh, K,; Liu, J.; Maddila, S.; Mouradian, M.M. Posttranslational Modifications of α-Synuclein, Their Therapeutic Potential, and Crosstalk in Health and Neurodegenerative Diseases.
Pharmacological Reviews. 2024, 76,1254. https://doi.org/10.1124/pharmrev.123.001111.
.

(S6) Wilkinson, M.; Xu, Y.; Thacker, D.; Taylor, A. I. P.; Fisher, D. G.; Gallardo, R. U.; Radford, S. E.; Ranson, N. A. Structural Evolution of Fibril Polymorphs during Amyloid Assembly. Cell 2023, 186 (26), 5798-5811.e26. https://doi.org/10.1016/j.cell.2023.11.025
