# Supplementary material for: Structural Hierarchy and Maturation of Amyloid Fibrils Revealed by Interface Descriptor Analysis
Source: J Chem Inf Model. 2026 Aug 21;66(17):11471–90. doi: 10.1021/acs.jcim.6c01376 (PMC13580560; doi:10.1021/acs.jcim.6c01376)

Agglomerative clustering result of protofilaments, shown as a heat map of $RMSD_{mod}$ values. The dendrogram represents the relative variance in the clustering. Clusters (assigned with a limiting value of 30% ot the total $RMSD_{mod}$) are shown as the first colored row(color-coded as *C1*: orange, *C2*: green, *C3*: purple, *C4*: red, *C5*: brown) . The second colored row denotes the origin of the structure (cyan for in vitro, and dark cyan for ex vivo)

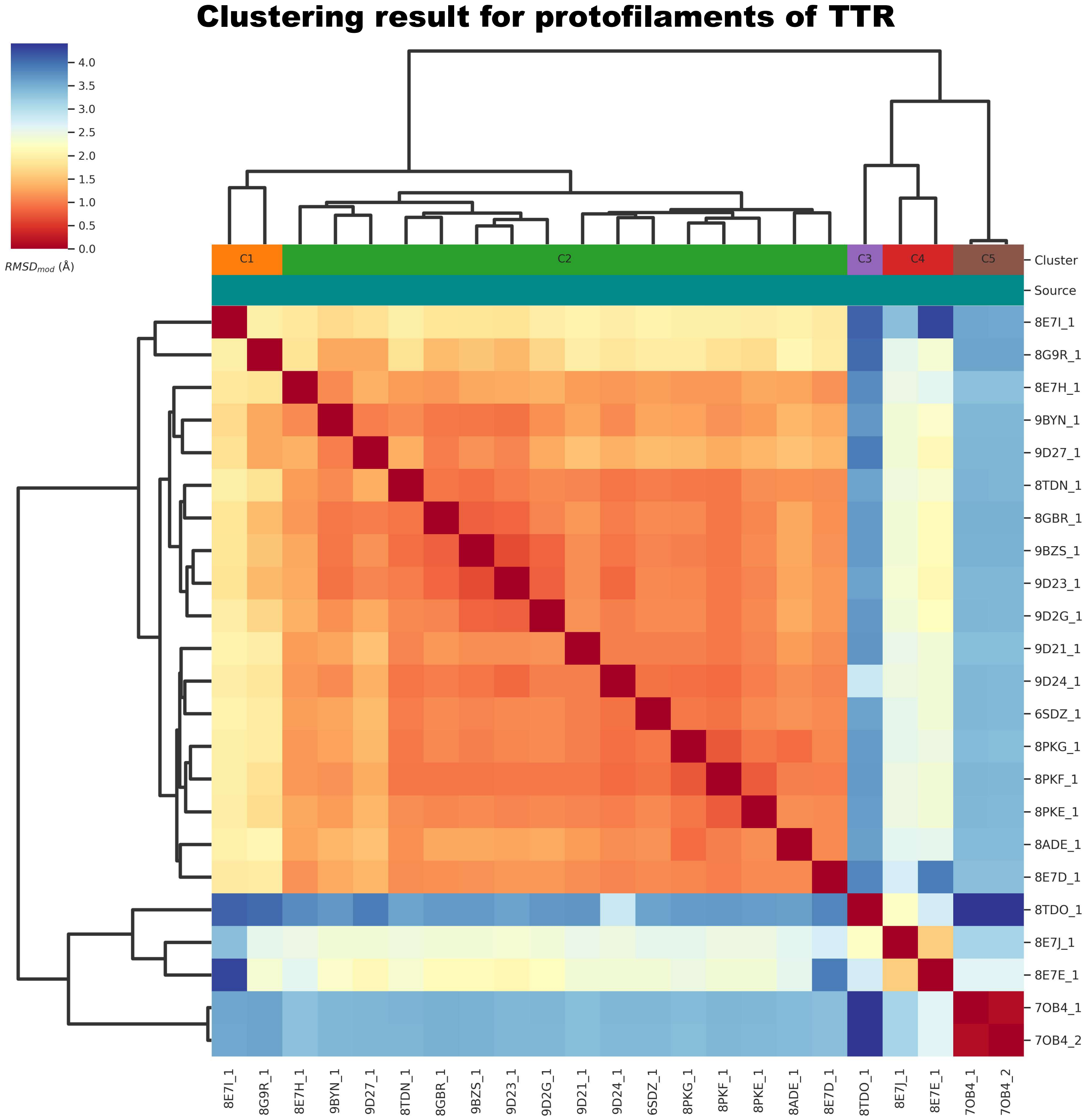

Local descriptor values plotted against the amino acid sequence as heatmaps. (white: missing residues; rose: residues with no local interface or no side chain) Clusters shown as the first colored column color coded as in the figure of clustering results. The second colored column denotes the origin of the structure (cyan for in vitro, and dark cyan for ex vivo)

Heatmap of descriptor values along the polypeptide sequence for individual structures of TTR

Sc

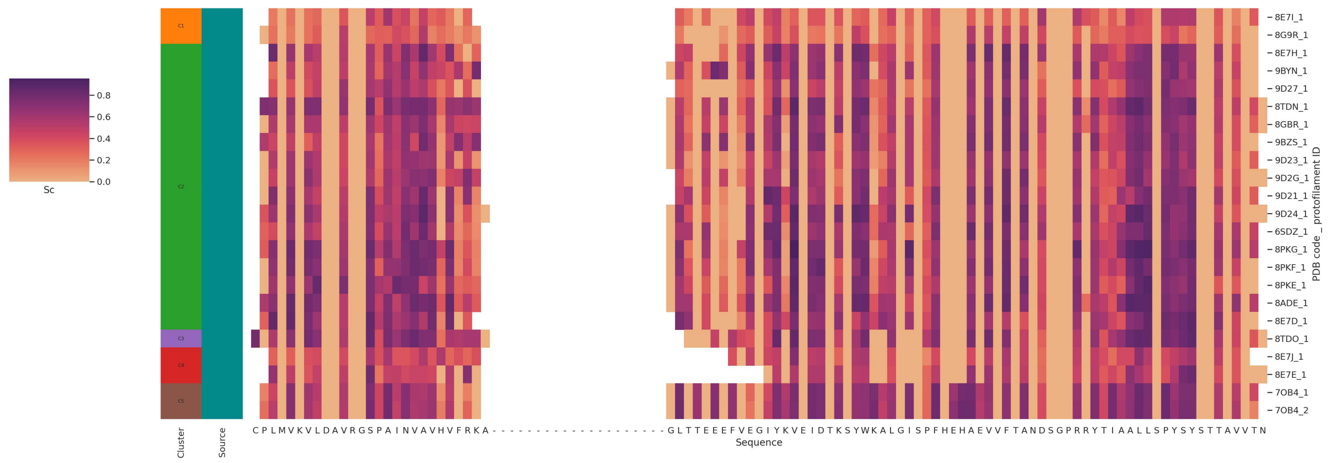

Local descriptor values plotted against the amino acid sequence as heatmaps. (white: missing residues; rose: residues with no local interface or no side chain) Clusters shown as the first colored column color coded as in the figure of clustering results. The second colored column denotes the origin of the structure (cyan for in vitro, and dark cyan for ex vivo)

Heatmap of descriptor values along the polypeptide sequence for individual structures of TTR

Ab

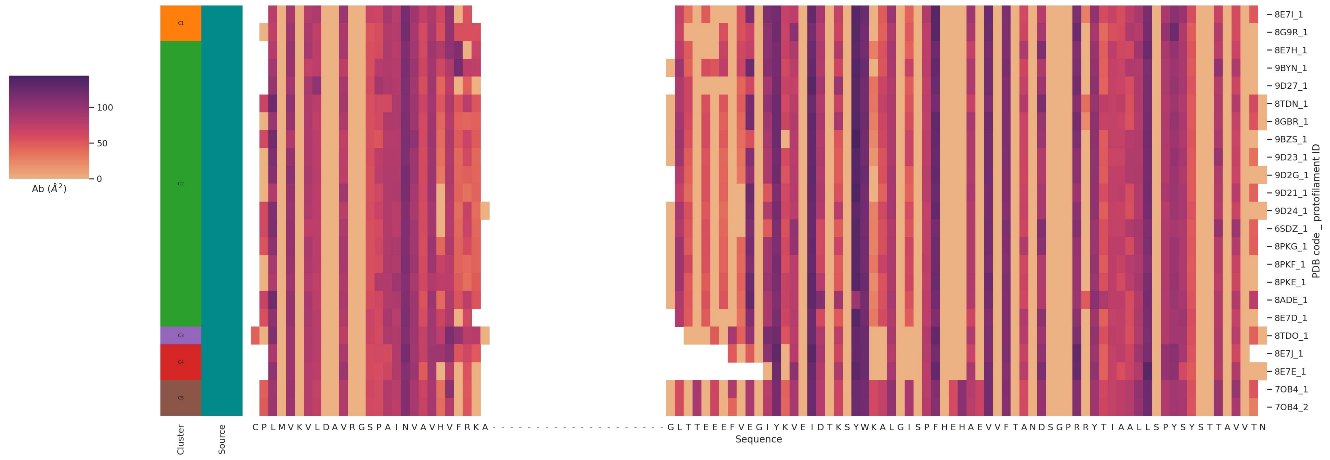

Local descriptor values plotted against the amino acid sequence as heatmaps. **(**white: missing residues; rose: residues with no local interface or no side chain) Clusters shown as the first colored column color coded as in the figure of clustering results. The second colored column denotes the origin of the structure (cyan for in vitro, and dark cyan for ex vivo)

Heatmap of descriptor values along the polypeptide sequence for individual structures of TTR

SDi

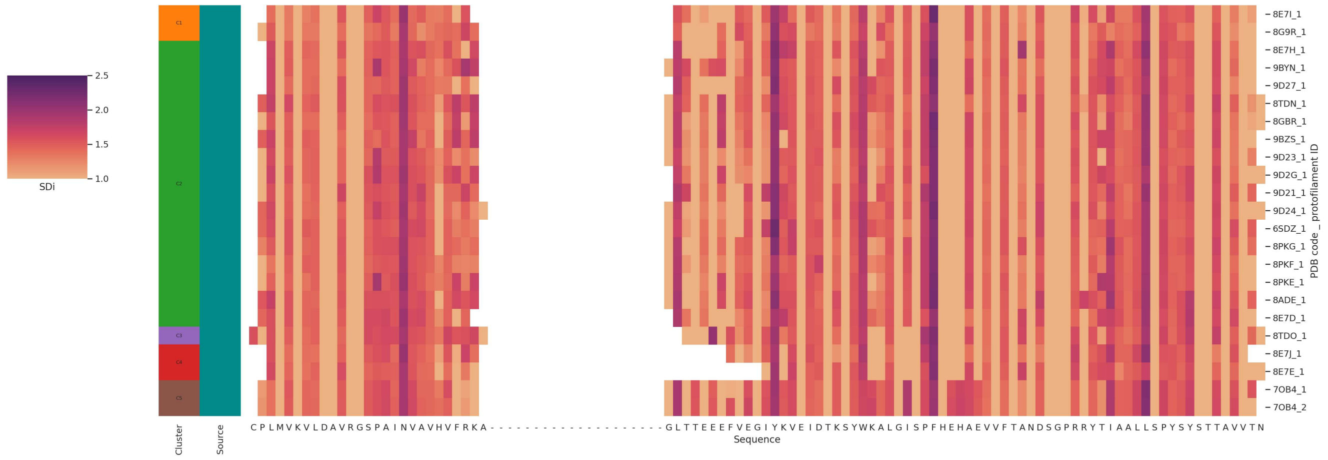

Heatmap of descriptor values along the polypeptide sequence, averaged values for each cluster of TTR

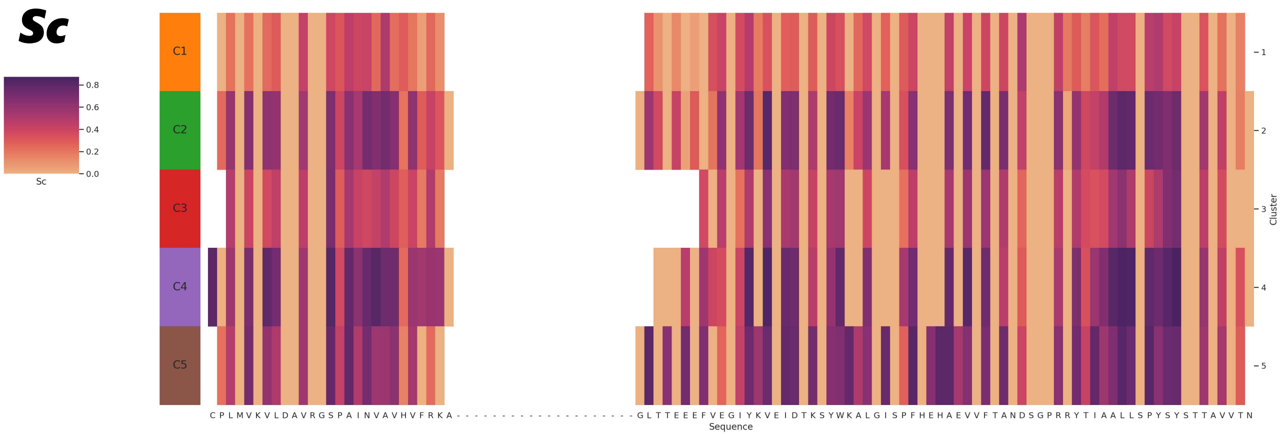

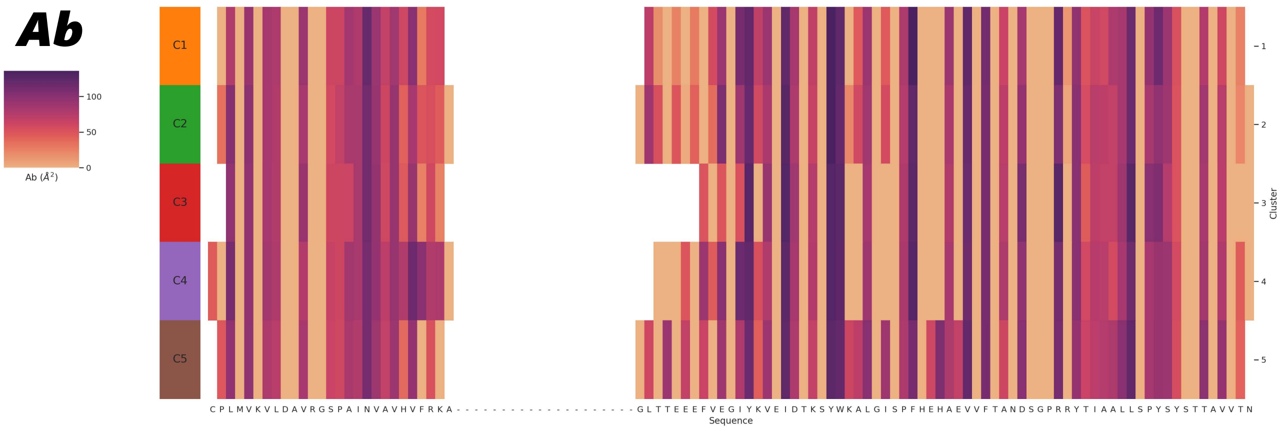

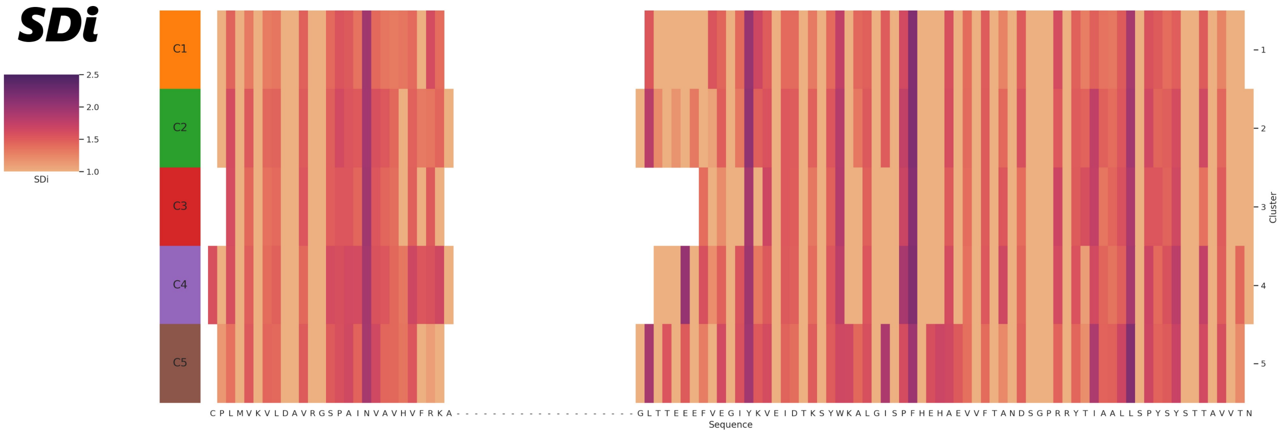

Local descriptor values plotted against the amino acid sequence as heatmaps – *calculated only for inter-protofilament interfaces*. (white: missing residues; light green: residues with no localinter-protofilament interface or no side chain) Clusters shown as the first colored column color coded as in the figure of clustering results. The second colored column denotes the origin of the structure (cyan for in vitro, and dark cyan for ex vivo)

Heatmap of descriptor values along the polypeptide sequence for individual structures of TTR, *calculated only for inter-protofilament interfaces*

Sc

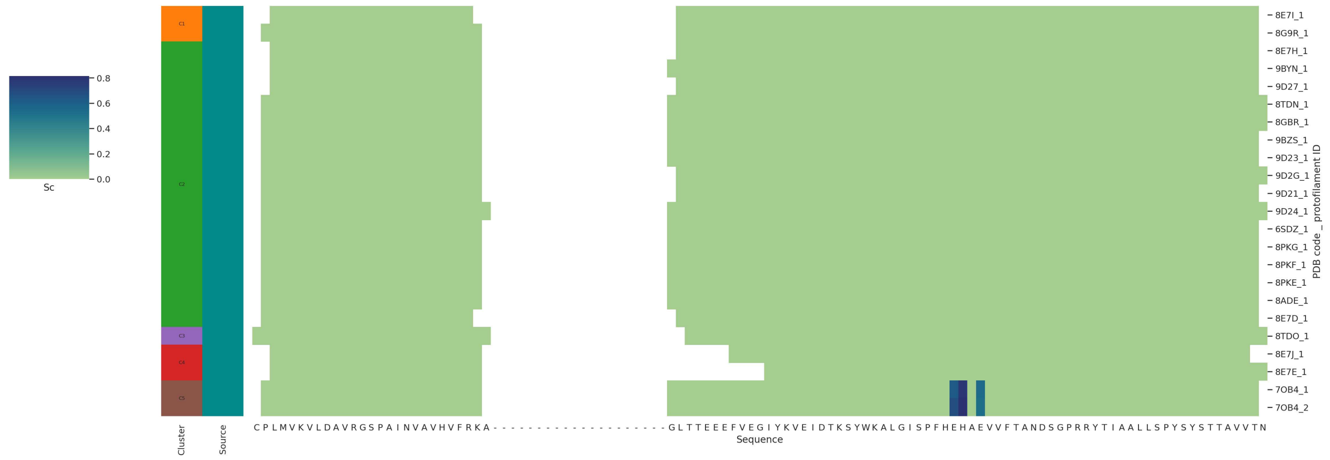

Local descriptor values plotted against the amino acid sequence as heatmaps – *calculated only for inter-protofilament interfaces*. (white: missing residues; light green: residues with no localinter-protofilament interface or no side chain) Clusters shown as the first colored column color coded as in the figure of clustering results. The second colored column denotes the origin of the structure (cyan for in vitro, and dark cyan for ex vivo)

Heatmap of descriptor values along the polypeptide sequence for individual structures of TTR, *calculated only for inter-protofilament interfaces*

Ab

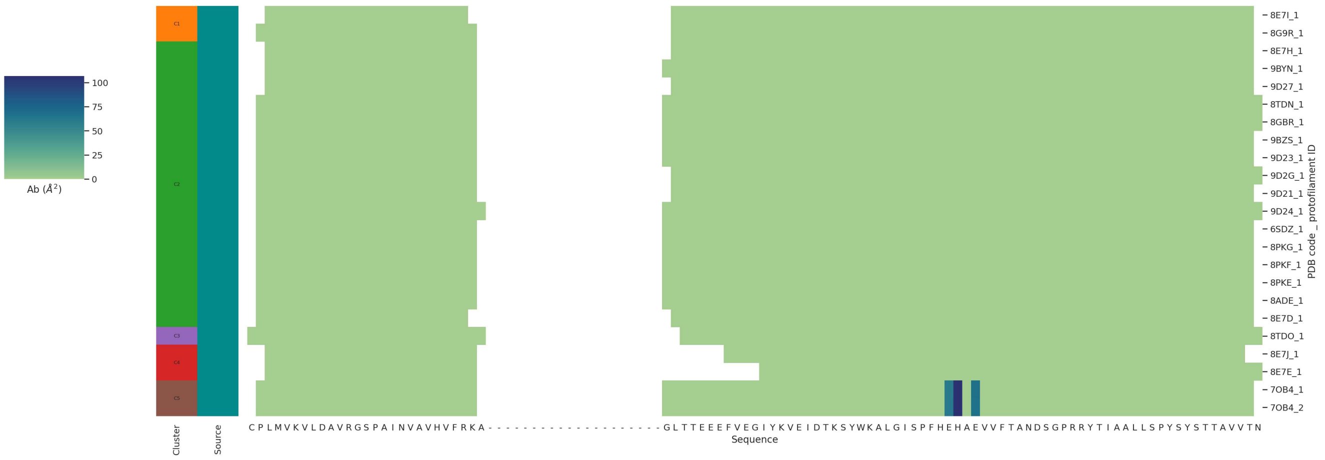

Local descriptor values plotted against the amino acid sequence as heatmaps – *calculated only for inter-protofilament interfaces*. (white: missing residues; light green: residues with no localinter-protofilament interface or no side chain) Clusters shown as the first colored column color coded as in the figure of clustering results. The second colored column denotes the origin of the structure (cyan for in vitro, and dark cyan for ex vivo)

Heatmap of descriptor values along the polypeptide sequence for individual structures of TTR, *calculated only for inter-protofilament interfaces*

SDi

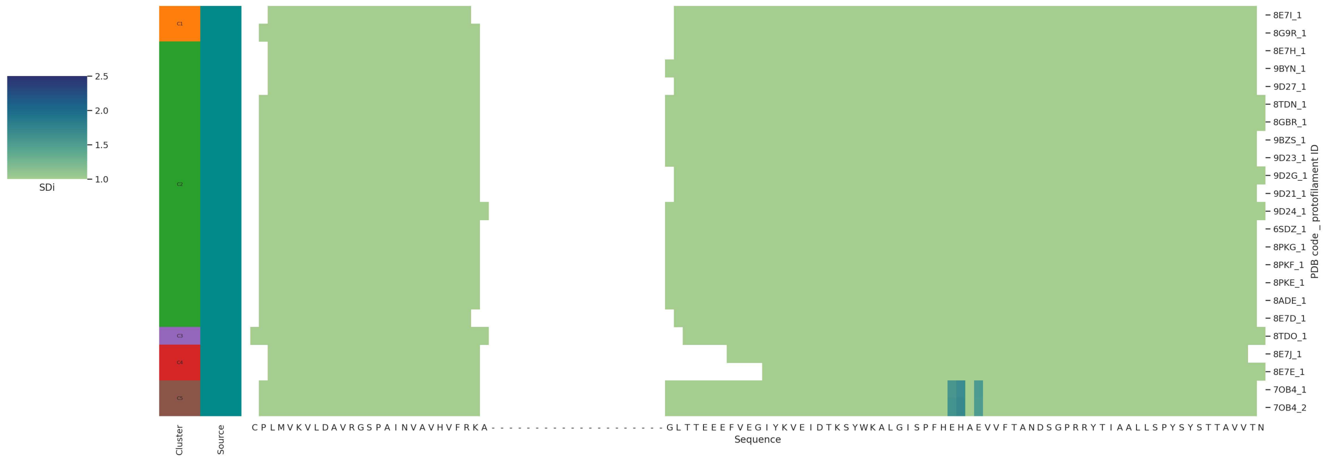

Heatmap of descriptor values along the polypeptide sequence, averaged values for each cluster of TTR, *calculated only for inter-protofilament interfaces*

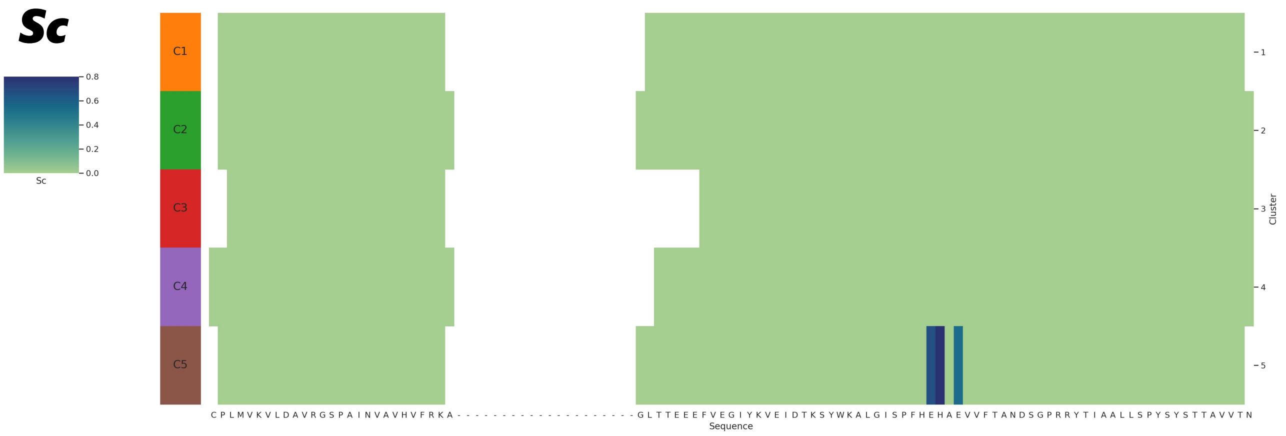

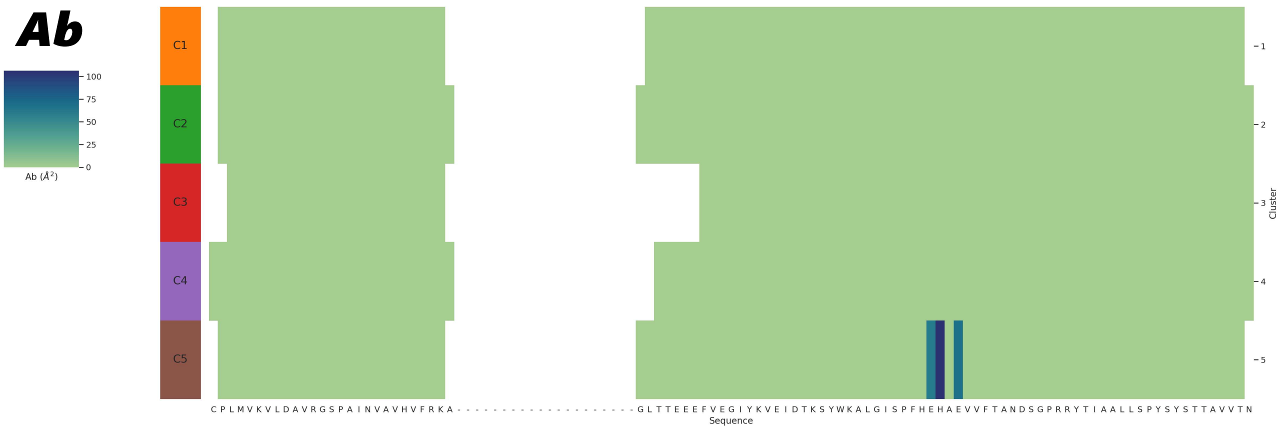

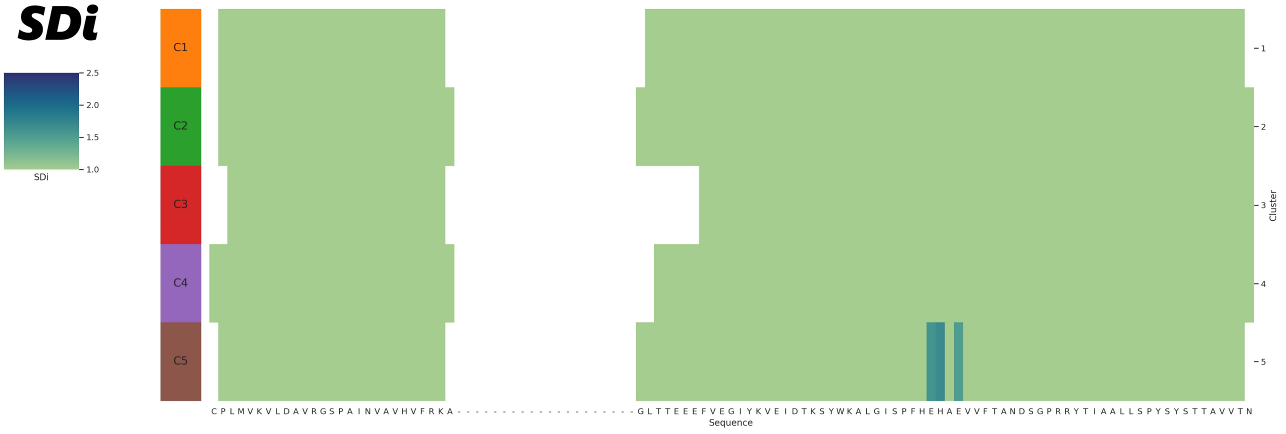

Distributions of descriptor values for each cluster of TTR structures, shown as kernel density estimate profiles of probability densities for each cluster. Clusters shown as the first colored column color coded as in the figure of clustering results.

Sc

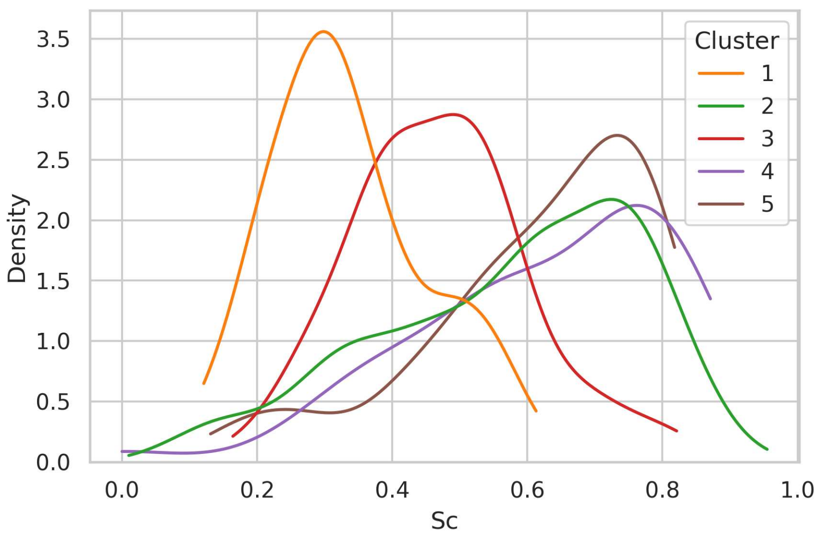

Ab

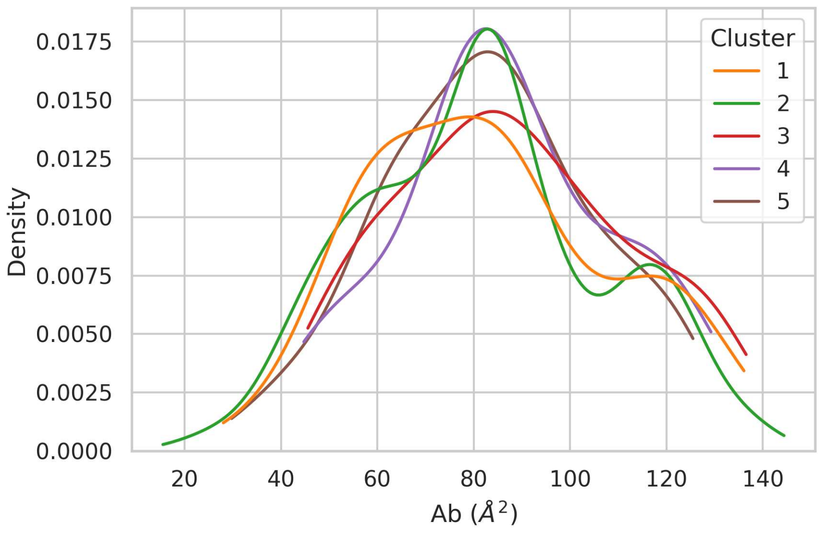

SDi

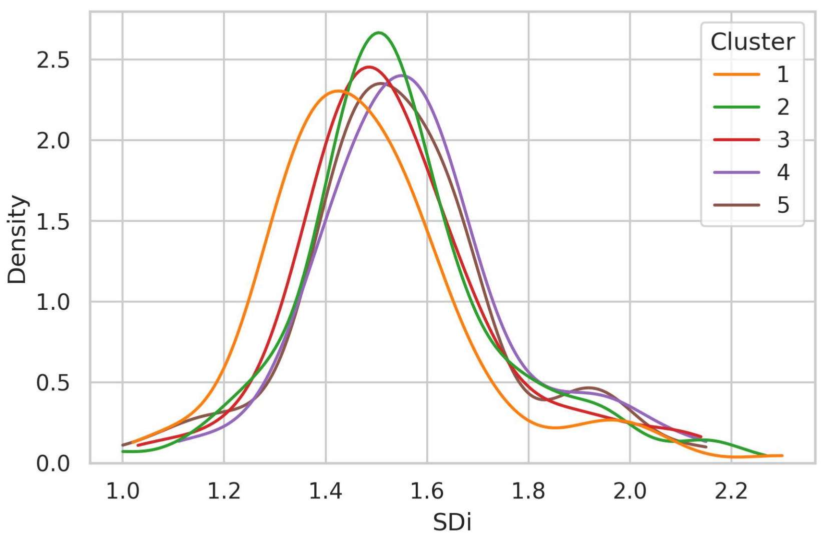

Distributions of descriptor values for TTR structures shown as kernel density estimate profiles of probability densities for in vitro source(cyan) and ex vivo source (dark cyan).

Sc

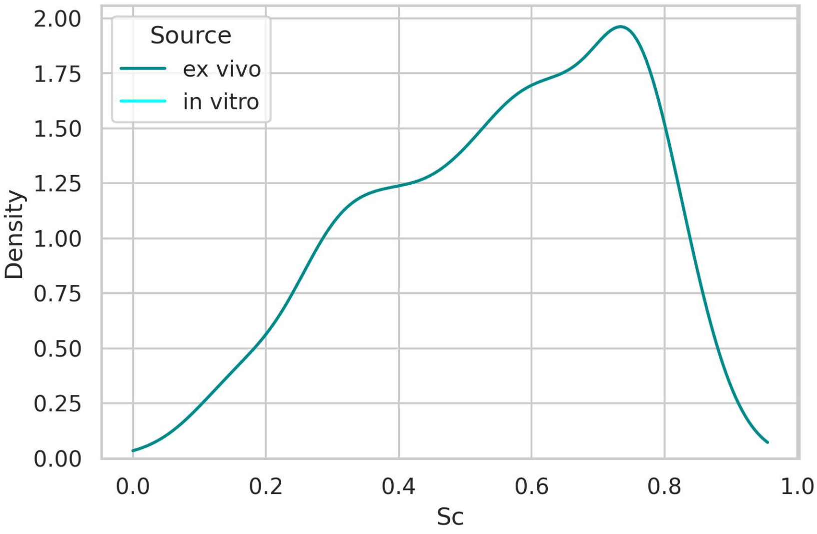

Ab

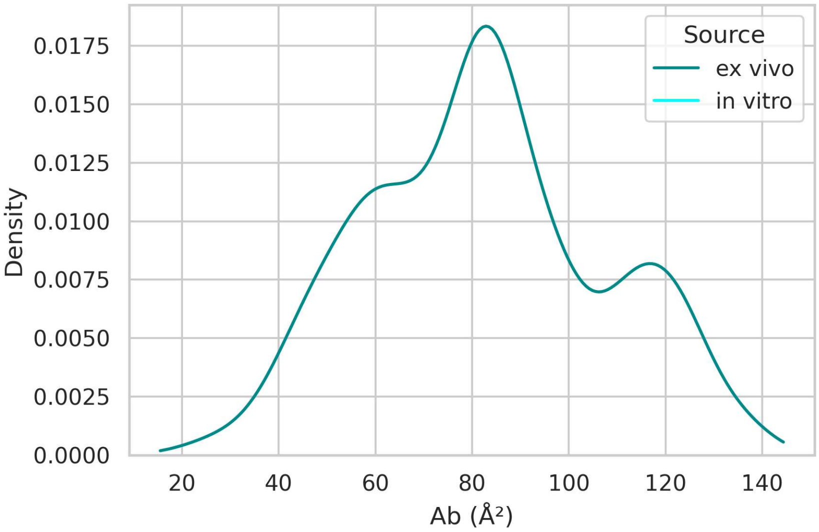

SDi

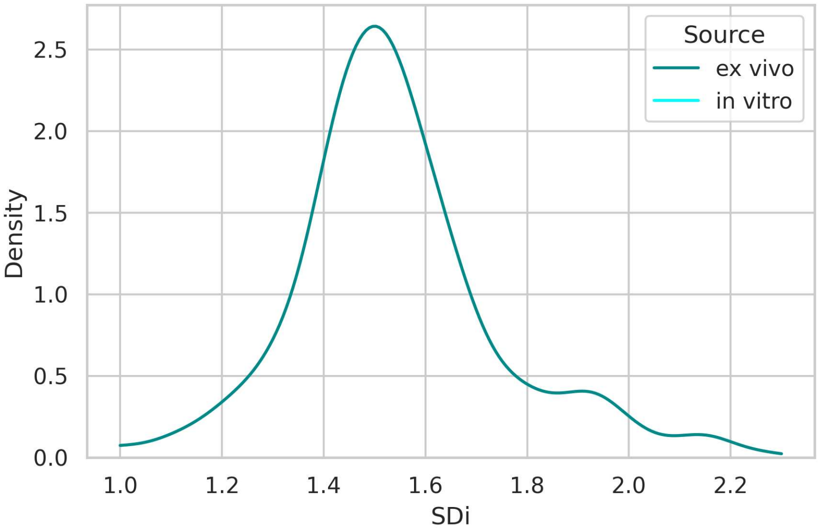

Heatmap of signature residues in hotspots along the polypeptide sequence of TTR. Signature residues were selected for precise steric fit and interface embedding (highest Sc and SDi values). Overall packing tightness is quantified by the number of residues with >90% of maximum Sc and SDi values. Residues with top 5 highest Sc values colored red, highest SDi values blue, purple for both.

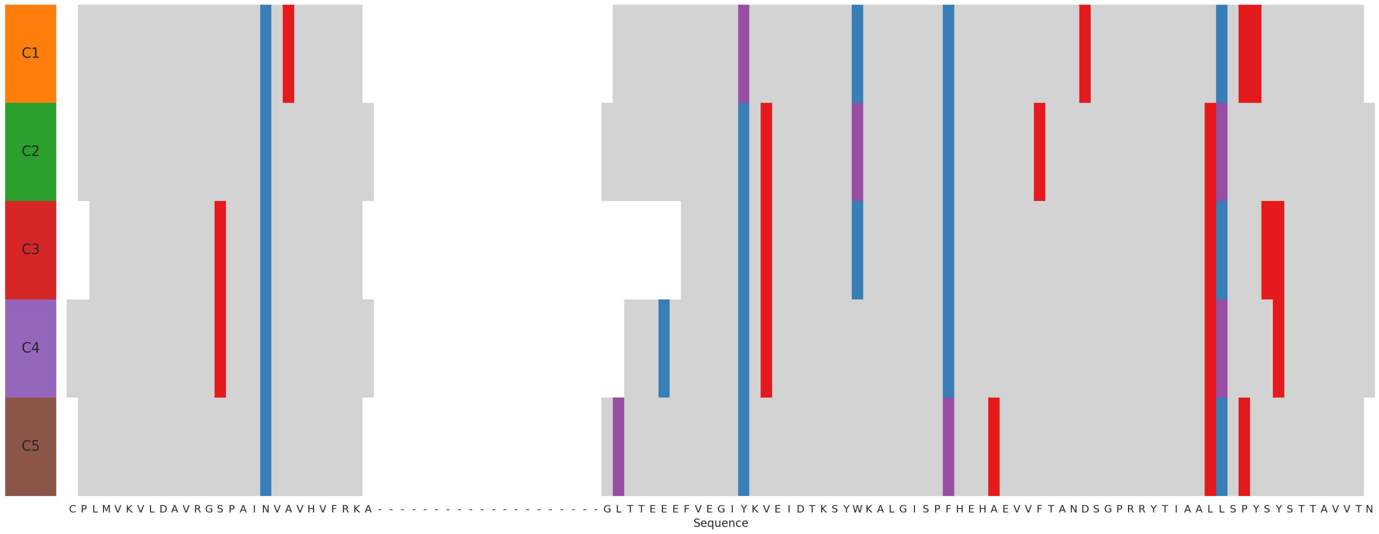

Supplement: Supplementary file 2 [file ci6c01376_si_001.zip › supplement-TTR.pdf]
